# Using xenon difluoride and 2 Li[Al{OC(CF_3_)_3_}_4_] as an oxidant: from organoxenonium intermediates to (fluoro-)biphenyl radical cations

**DOI:** 10.1039/d6sc01402j

**Published:** 2026-05-05

**Authors:** Konstantin Kloiber, Tim Heizmann, Benoît Lacombe, Philipp Thielert, Malte Sellin, Tim Schwandt, Sabine Richert, Stefan Weber, Ingo Krossing

**Affiliations:** a Institute of Inorganic and Analytical Chemistry, Freiburg Materials Research Center (FMF), University of Freiburg Albertstr. 21 79104 Freiburg Germany krossing@uni-freiburg.de; b Institute of Physical Chemistry, University of Freiburg Albertstr. 21 79104 Freiburg Germany; c Institute of Physical Chemistry II, University of Ulm Lise-Meitner-Str. 16 89081 Ulm Germany

## Abstract

The feasibility of employing the XeF_2_/2Li[Al(OR^F^)_4_] (OR^F^ = OC(CF_3_)_3_) system as a deelectronator, formally generating Xe_(g)_ + 2LiF_(s)_ + 2□^+^, was investigated. While this proved successful for substrates like ferrocene and 9,10-dichlorooctafluoroanthracene, the system was found to react with the solvents benzene, fluorobenzene (FB) and 1,2-difluorobenzene (2FB). The reaction led to the formation of biphenyl radical cations as arene coupling products. In contrast, the reaction with 1,2,3-trifluorobenzene (3FB) allowed the observation of a persistent organoxenonium ion. Low-temperature NMR control reactions and quantum chemical calculations suggest a xenonium-mediated coupling reaction with organoxenonium ions as intermediates. As a reference for the organoxenonium ions proposed, the perfluorinated tight ion-pair (C_6_F_5_)Xe-F-Al(OR^F^)_3_ and the salt [C_6_F_5_Xe][F{Al(OR^F^)_3_}_2_] were synthesized to assess their stability in combination with the alkoxyaluminate anions used in this work. Oxidation studies showed pronounced solvent effects, with strong oxidation observed in pentafluorobenzene, while in MeCN, ferrocene was found to be oxidized already by XeF_2_ alone. These findings not only expand the synthetic utility of XeF_2_-based oxidation systems but also provide new insight into the reactivity of organoxenonium ions, contributing to the growing understanding of the role of xenon chemistry in selective oxidative transformations.

## Abstract 2

The combination of XeF_2_ with Li[Al(OR^F^)_4_] enables the formation of (fluorinated) biphenyl radical cations *via* organoxenonium intermediates, even at low temperatures.

## Graphical abstract

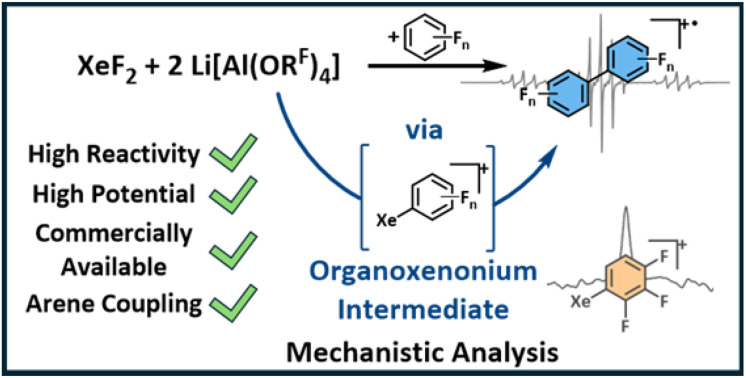

## Introduction

The development of high potential oxidants has experienced renewed interest over the past decade, particularly by combination of oxidant cations with weakly coordinating anions (WCAs).^1–8^ The availability of robust WCAs, *e.g.* [Al(OR^F^)_4_]^−^, [(R^F^O)_3_Al–F–Al(OR^F^)_3_]^−^ (OR^F^ = OC(CF_3_)_3_), [Al(OTeF_5_)_4_]^−^ or [(C_6_F_5_)_3_B-CN-B(C_6_F_5_)_3_]^−^, enables the stabilization of highly reactive cations and allows redox potentials to be shifted to increasingly positive values.^9–12^ Very recently, this field has been expanded by the development of new aluminum-based Lewis superacids and related weakly coordinating anions derived from organotellurium ligands.^13^ Two general strategies are commonly employed to access reactive cations stabilized by WCAs: (1) metathesis reactions by using for example Li^+^, Ag^+^ or [CPh_3_]^+^ salts for halide abstraction,^8,12,14,15^ and (2) direct oxidation of the substrate with a high potential WCA-oxidant salt (=Ox[WCA]).^4,7,14,16,17^ Here, inorganic reagents such as NO[Al(OR^F^)_4_], Cu[Al(OR^F^)_4_] or Ag[Al(OR^F^)_4_] are readily accessible through a simple metathesis reaction from the corresponding (even commercial)^18^ lithium salt and reach very high (solvent-dependent) oxidation potentials up to 1.52 V (NO^+^; 4FB = 1,2,3,4-F_4_C_6_H_2_) and 1.50 V (Cu^+^, Ag^+^, 5FB = C_6_F_5_H) *vs.* ferrocene/ferrocenium (Fc^+/0^).^15,19–21^ Notably, borate-WCAs such as [B(C_6_F_5_)_4_]^−^ or [B(3,5-C_6_H_3_(CF_3_)_2_)_4_]^−^ anodically oxidize already at moderate potentials to give fluorinated biphenyls electrosynthetically in good yields, showcasing their limited stability at higher potentials.^22^ In addition, the solvent-free Ag[B(C_6_F_5_)_4_] decomposes to AgC_6_F_5_ and B(C_6_F_5_)_3_.^23^ Furthermore, although classical WCAs such as [MF_6_]^−^ (P, Sb, As) have high electrochemical stability, they tend to induce low solubilities and anion coordination in organic media; the latter is often followed by fluoride ion abstraction.^24–26^

**Fig. 1:**
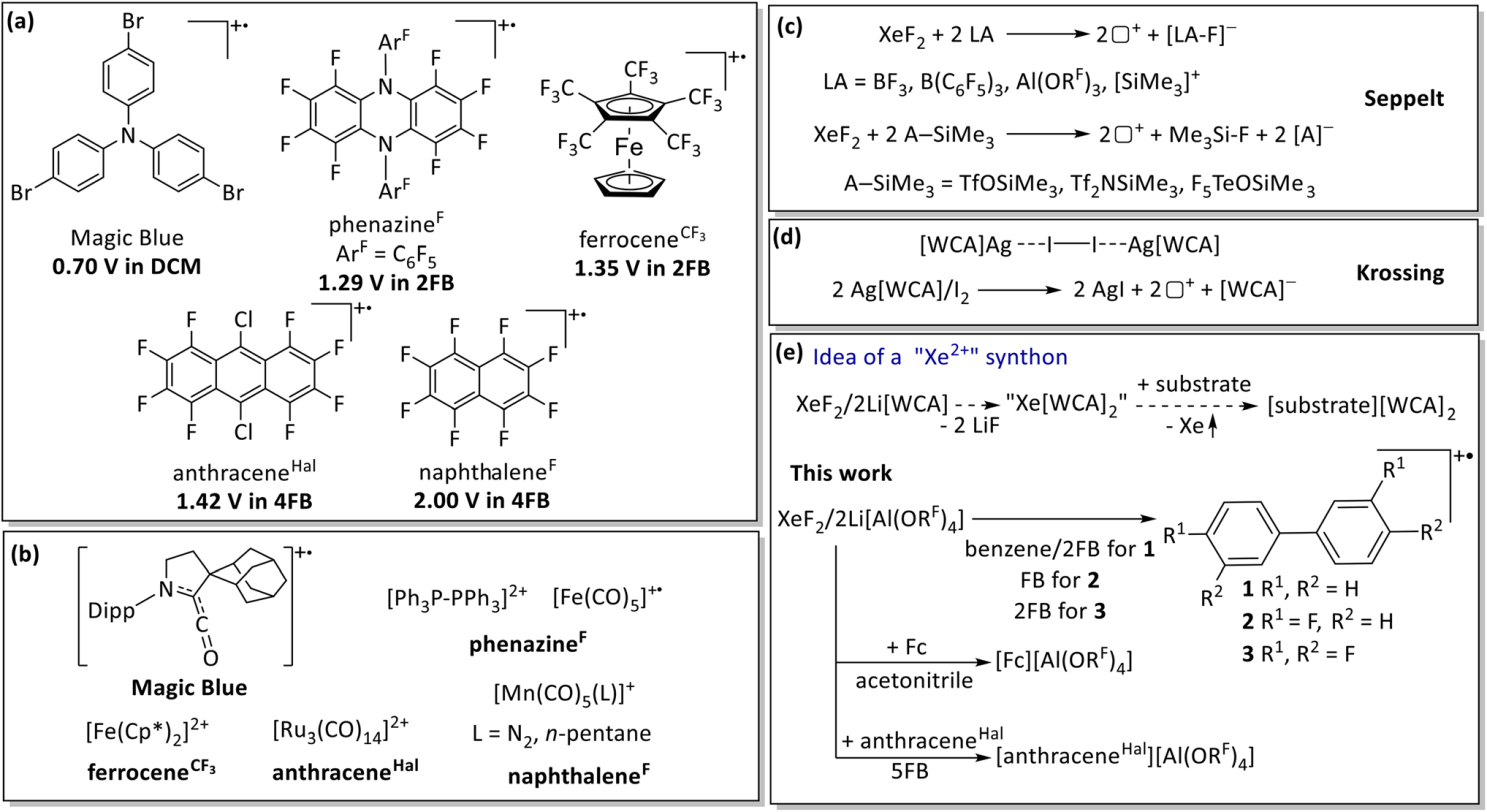
Selected examples for (a) selective deelectronators including their half wave potential given *vs.* Fc^+/0^ and (b) some selected applications to prepare unusual compounds in synthetic chemistry.^4,17,32–34,42–46^ The generated cation is shown in regular font with summary or structural formula and the deelectronator reagents in bold using the abbreviation defined in (a). Cp* = C_5_Me_5_; Dipp = 2,6-^*i*^Pr_2_-C_6_H_3_. (c) Application of XeF_2_ as an oxidative system in combination with Lewis acids (LA = BF_3_, B(C_6_F_5_)_3_, Al(OR^F^)_3_) or silylated derivatives of strong Brønsted acids (A-SiMe_3_ = TfOSiMe_3_, Tf_2_NSiMe_3_, F_5_TeOSiMe_3_).^3^ (d) The oxidative system 2Ag[Al(OR^F^)_4_]/I_2_.^47^ (e) This work: investigation of the oxidative system XeF_2_/2Li[Al(OR^F^)_4_] with different (fluorinated) arenes and evaluation of its oxidative potential in acetonitrile and 5FB (anthracene^Hal^ = 9,10-dichlorooctafluoroanthracene).

Yet, despite the fact that inorganic oxidants paired with excellent WCAs have enabled the isolation and study of novel reactive species and unusual oxidation states throughout the periodic system,^2,5,7,27–29^ reagents with NO^+^, Cu^+^, Ag^+^ or related active cations are limited by two major challenges:^30,31^ first, their redox potential is strongly influenced by the solvent and environment, and second, the cations tend to undergo side reactions by forming complexes with the substrate (*e.g.* Ag^+^ and Fe(CO)_5_) or undergoing ligand substitution (*e.g.*, NO^+^ and Fe(CO)_5_).^13,17,28,29^ Consequently, alternative oxidants have been developed in recent years to address these limitations. They act as selective deelectronators that do not react with the substrates through further bond-breaking and bond-making processes (Fig. 1a). With such reagents in hand, we and others were able to access new highly reactive species, *e.g.*, the [Fe(CO)_5_]^+^ metalloradical or the [Ph_3_P-PPh_3_]^2+^ dication (Fig. 1b).^4,17,32–34^

In this realm, XeF_2_ is known as a versatile reagent with strong oxidizing and fluorinating properties and, unlike higher xenon fluorides, is kinetically stable and comparatively easy to handle, which has made it a widely used reagent in synthesis.^35^ In the presence of strong fluoride acceptors or Lewis acids, XeF_2_ can be converted into highly reactive and strongly oxidizing systems that are commonly rationalized in terms of fluoride abstraction and [XeF]^+^-type reactivity.^36–38^ In particular, combinations of XeF_2_ with strong Lewis acids such as BF_3_ and B(C_6_F_5_)_3_, or Lewis superacids such as Al(OR^F^)_3_, have been shown to display markedly enhanced reactivity, ranging from electrophilic fluorination/dearomatization of fluoroarenes to oxidation of suitable substrates, depending on the reaction partner and medium (Fig. 1c).^3,37–41^

Building on this chemistry, the present study explores the “activation” of XeF_2_ by a combination of two equivalents of Li[Al(OR^F^)_4_] as an oxidizing system. In analogy to the known XeF_2_/2LA and the closely related 2 Ag[WCA]/I_2_ systems (Fig. 1c and d), our approach aims to exploit the formally possible 2e^−^ redox capabilities of the system XeF_2_ + 2Li^+^ → “Xe^2+^” + 2LiF → Xe_(g)_ + 2LiF_(s)_ + 2□^+^ (□^+^ = positive hole) by utilizing the thermodynamic driving forces of the formation of Xe gas and stable solid LiF (Δ_Latt_*H* = 1030 kJ mol^−1^, Fig. 1e).^48^ Here, we found the XeF_2_/2Li[Al(OR^F^)_4_] system to act as a deelectronator for simple substrates like Fc^0^, but also delocalized high potential systems like anthracene^Hal^ (1.42 V *vs.* Fc^+/0^) in MeCN or 5FB solution (Fig. 1a). This already underpins that the system reacts with the somewhat less oxidation stable solvents fluorobenzene (FB) and 1,2-difluorobenzene (2FB) to form biphenyl radical cations as arene coupling products. In contrast, 1,2,3-trifluorobenzene (3FB) does not yield radical cations but instead allows observation of a persistent organoxenonium ion. The mechanism of arene coupling and the stability of the involved organoxenonium ions were investigated.

## Results and discussion

### Initial observations

**Fig. 2:**
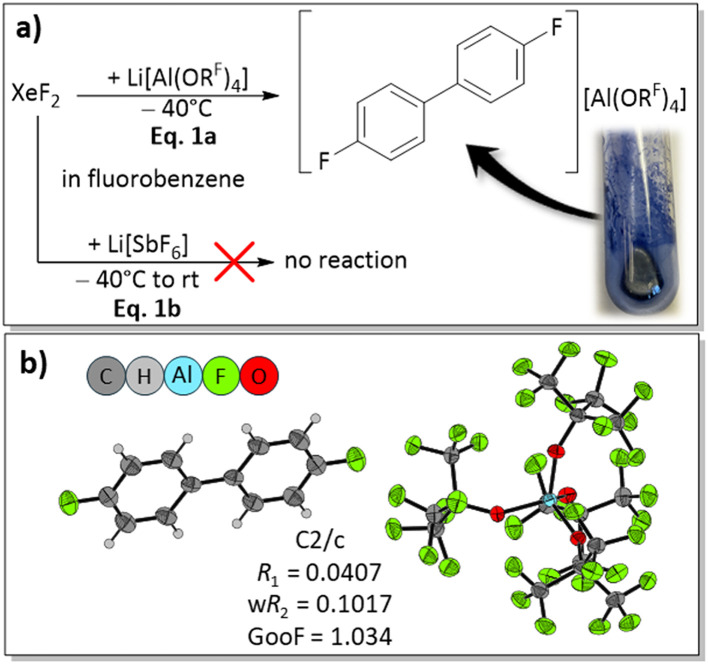
(a) Overall reaction scheme for the reaction of XeF_2_ with Li[Al(OR^F^)_4_] in fluorobenzene leading to blue crystals of [4,4′-difluorobiphenyl][Al(OR^F^)_4_]. In contrast, no reaction was observed when Li[SbF_6_] was used under these conditions, including a room temperature reaction. (b) Molecular structure of [4,4′-difluorobiphenyl][Al(OR^F^)_4_]. Thermal displacement ellipsoids are shown at a 50% probability level.

To investigate whether commercially available Li[Al(OR^F^)_4_] can serve to activate XeF_2_ as a potential two-electron oxidant, the reaction of XeF_2_ with two equivalents of Li[Al(OR^F^)_4_] in fluorobenzene (FB) was chosen as a starting point. Immediately after XeF_2_ was added to the Li[Al(OR^F^)_4_] solution, the reaction mixture turned intensely blue, potentially indicating the formation of radicals (Fig. 2a, eqn (1a)).^49^ The remarkable speed in color change suggested that the lithium salt of the alkoxyaluminate is a promising source for XeF_2_ activation. In contrast, the analogous reaction employing Li[SbF_6_] resulted in no observable changes, neither at −40 °C nor at room temperature (Fig. 2a). This is probably caused by the high lattice energy of Li[SbF_6_] and therefore its poor solubility in FB. When the deep blue reaction mixture resulting from reaction with Li[Al(OR^F^)_4_] was layered with *n*-pentane and stored at −35 °C, some blue crystals of [4,4′-difluorobiphenyl][Al(OR^F^)_4_] suitable for scXRD were isolated (scXRD = single crystal X-ray diffraction; Fig. 2b). Triggered by this observation, the reaction was investigated in more depth.

## Scope of the reaction and characterization

To investigate the generality of the reaction in Fig. 2a, the system XeF_2_/2Li[Al(OR^F^)_4_] was also used in combination with a mixture of benzene in 1,2-difluorobenzene (2FB) and in pure 2FB. The reaction of XeF_2_/2Li[Al(OR^F^)_4_] with benzene/2FB led to an intensely yellow colored solution, while the system in 2FB also had an intense blue color. After quenching these reactions by adding H_2_O and stirring under atmospheric conditions, followed by work up including purification by column chromatography (Silica 60/*n*-pentane), the neutral species biphenyl (1, bb), 4,4′-difluorobiphenyl (2, fbfb) and 3,3′,4,4′-tetrafluorobiphenyl (3, 2fb2fb) were isolated and characterized by NMR spectroscopy.

**Fig. 3:**
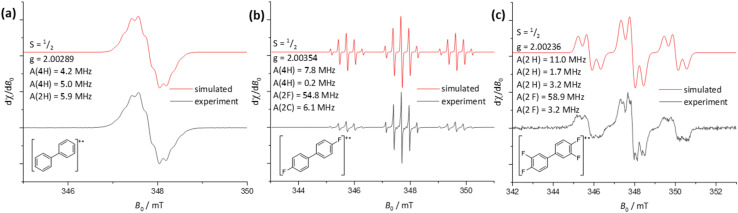
Continuous wave EPR spectra of the reaction solutions of XeF_2_/2Li[Al(OR^F^)_4_] with (a) benzene in 2FB, (b) FB and (c) 2FB. All reactions were performed at −40 °C. The resulting deep yellow (a) or deep blue (b + c) reaction solutions were transferred to EPR tubes under an inert atmosphere and subsequently measured at room temperature.

**Scheme 1:**
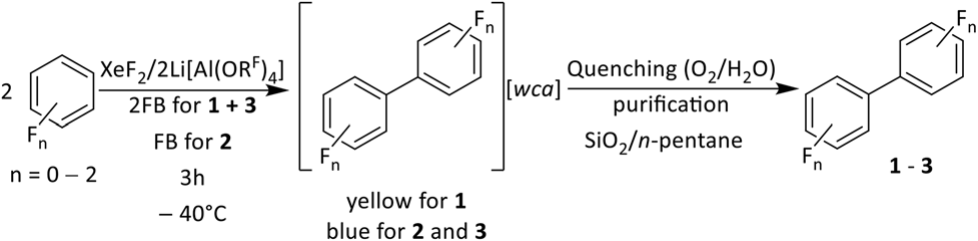
Reaction of XeF_2_/2Li[Al(OR^F^)_4_] with benzene, fluorobenzene (FB) and 1,2-difluorobenzene (2FB) yielding the homocoupling products biphenyl (1, bb), 4,4′-difluorobiphenyl (2, fbfb) and 3,3′,4,4′-tetrafluorobiphenyl (3, 2fb2fb), respectively. In the initial step, one equivalent of XeF_2_/2Li[Al(OR^F^)_4_] leads to the neutral coupling product and another equivalent of XeF_2_/2Li[Al(OR^F^)_4_] generates the radical cations of the coupling product. The work up includes stirring the reaction mixtures under atmospheric conditions and purification of 1, 2 and 3 by column chromatography.

EPR analyses were performed on the intensely colored solutions, leading after work up to 1–3. The simulations of the EPR spectra agree with their assignment as the radical cations [1]˙^+^, [2]˙^+^ and [3]˙^+^ (see Fig. 3) and prove the formation of the radical cations in Scheme 1.

**Fig. 4:**
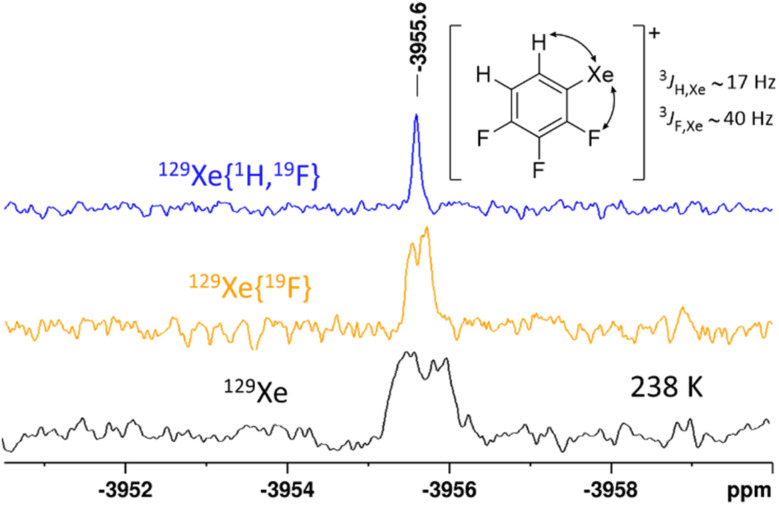
^129^Xe NMR spectra (111.29 MHz) of the reaction XeF_2_/2Li[Al(OR^F^)_4_] in a fluorinated benzene solvent mixture (3FB/5FB) at 238 K. The signal of the observed organoxenonium ion [C_6_F_3_H_2_Xe]^+^ is shown in three different decoupling schemes: ^129^Xe in black, ^129^Xe{^19^F} in yellow and ^129^Xe{^1^H, ^19^F} in blue. All coupling constants shown here have an uncertainty of 2–3 Hz.

In contrast to the three reactions of benzene, FB and 2FB, the reaction of 1,2,3-trifluorobenzene (3FB) in 5FB proceeds more slowly and is accompanied by a color change of the solution to brown. In the EPR spectrum of the reaction mixture, no paramagnetic species were detected. Low-temperature NMR analysis of the reaction mixture revealed several fluorinated arenes in the ^19^F NMR spectrum, including a species identified as the organoxenonium ion [2,3,4-C_6_H_2_F_3_Xe]^+^ (see Fig. 4). Remarkably, this species could also be observed at room temperature. Note that two factors likely contribute to the stability of xenonium ions in the present case: (1) stabilization by the electron-withdrawing effect of the fluorine substituents in the *ortho*- and *para*-position and (2) for *ortho*-fluorinated substrates, an additional fluorine-xenon interaction/chelate effect.^49^ Both likely account for the stability of this xenonium ion. Apparently, the enhanced stability of the observed xenonium ion and the low reactivity of the π-system of 3FB are the reasons why the reaction stops at this stage (see below, Scheme 5, proposed mechanism, step C). However, common side reactions of excess XeF_2_, such as the fluorination of arenes, predominate under these conditions.^50^

## Stability of XeF_2_/2Li[Al(OR^F^)_4_] and its potential

**Scheme 2:**
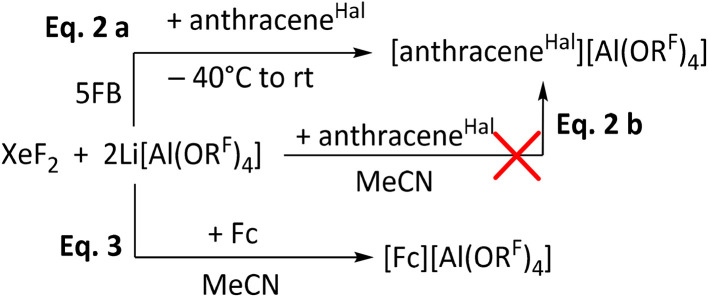
Tests on the oxidation potential of XeF_2_/2Li[Al(OR^F^)_4_]: oxidation of anthracene^Hal^ (=9,10-dichlorooctafluoro-anthracene) to [anthracene^Hal^][Al(OR^F^)_4_] in 5FB, but not in acetonitrile. In contrast, ferrocene is already oxidized in MeCN by XeF_2_ alone and Li[Al(OR^F^)_4_] acts as a metathesis reagent affording Fc[Al(OR^F^)_4_].

After establishing that XeF_2_/2Li[Al(OR^F^)_4_] induces coupling of lower fluorinated arenes, like FB and 2FB, the system was investigated using 5FB as solvent, which has the highest published positive electrochemical solvent limit of all fluorobenzenes of +2.67 V *vs.* Fc^+/0^.^19^ XeF_2_ alone was found to be stable at room temperature for up to two hours, after which fluorination of 5FB was observed (SI 4.1). Subsequently, the mixture of XeF_2_ and 2Li[Al(OR^F^)_4_] was analyzed by NMR spectroscopy at −35 °C. This analysis revealed that similar fluorination reactions occur rapidly in this mixture even at low temperatures. Therefore, the addition of XeF_2_ to a solution of Li[Al(OR^F^)_4_] in the presence of a redox active substrate appeared as a logical consequence to estimate its potential. For this purpose, 9,10-dichlorooctafluoroanthracene (anthracene^Hal^) was selected. Upon adding XeF_2_ and gradual warming to rt, a color change to deep green took place, characteristic of the radical cation [anthracene^Hal^]˙^+^ (Scheme 2). This indicates that XeF_2_/2Li[Al(OR^F^)_4_] in 5FB reaches a potential of at least 1.42 V *vs.* Fc^+/0^.^32^ In addition to 5FB, acetonitrile was also considered as a potential solvent. The observed stability of XeF_2_ in acetonitrile under the present conditions is consistent with previous literature reports, and XeF_2_ was found to remain stable for several days in this solvent (SI 4.2).^51^ While XeF_2_/2Li[Al(OR^F^)_4_] can oxidize Fc in acetonitrile, anthracene^Hal^ could not be oxidized in this solvent (Scheme 2). Notably, control experiments demonstrated that Fc can already be oxidized by XeF_2_ alone in MeCN, suggesting that Li[Al(OR^F^)_4_] primarily acts as a metathesis reagent to afford Fc[Al(OR^F^)_4_]. These findings indicate that the enhanced reactivity of the Xe/2Li[Al(OR^F^)_4_] system is suppressed in MeCN, likely due to the increased solvation and consequently reduced reactivity of the Li^+^ ion in this system.

## Synthesis of [C_6_F_5_Xe]^+^-compounds and stability with aluminate WCAs

Various counterions have hitherto been used to synthesize and stabilize organoxenonium ions, including tetrafluoroborate and related fluoroborate-derived anions, and more recent work has further broadened the synthetic access to arylxenonium(II) salts.^49,52–61^ However, to date, no examples are reported utilizing perfluorinated alkoxyaluminate anions as WCAs. In combination with the above assignment of the [2,3,4-C_6_H_2_F_3_Xe]^+^ intermediate, this raised the question of whether organoxenonium species are compatible with aluminate anions, or if the observed decomposition of the anion is a result of such incompatibility. To address this, we aimed to synthesize salts with the well-established [C_6_F_5_Xe]^+^ cation and aluminate-WCAs as the counterion to directly assess their compatibility.

### A modified route to [C_6_F_5_Xe]^+^ salts

**Scheme 3:**
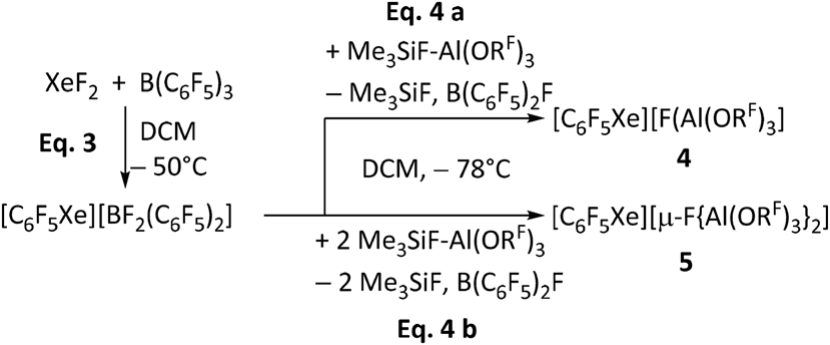
Synthesis of the compounds [C_6_F_5_Xe][WCA] ([WCA]^−^ = [FAl(OR^F^)_3_]^−^ (4), [µ-F{Al(OR^F^)_3_}_2_]^−^ (5)) starting from the established synthesis of [C_6_F_5_Xe][BF_2_(C_6_F_5_)_2_] by Frohn *et al.*^53^

The established synthesis of the organoxenonium ion [C_6_F_5_Xe][AsF_6_] with the intermediate [C_6_F_5_Xe][BF_2_(C_6_F_5_)_2_] served as the starting point for our route (see Scheme 3).^53^ To obtain the xenonium compounds, our route uses the “ion-like” trimethylfluorosilane adduct (CH_3_)_3_SiF–Al(OR^F^)_3_ as a strong Lewis acid (FIA = 459 kJ mol^−1^)^62,63^ to abstract a fluoride ion from [BF_2_(C_6_F_5_)_2_]^−^. Depending on the number of equivalents of Me_3_SiF–Al(OR^F^)_3_ employed, we successfully synthesized [C_6_F_5_Xe][WCA] ([WCA]^−^ = [FAl(OR^F^)_3_]^−^ (4), [µ-F{Al(OR^F^)_3_}_2_]^−^ (5), see Scheme 3). Both compounds 4 and 5 were characterized by NMR, IR and scXRD (SI 5.7, 5.8, 8.2 and 9).

**Fig. 5:**
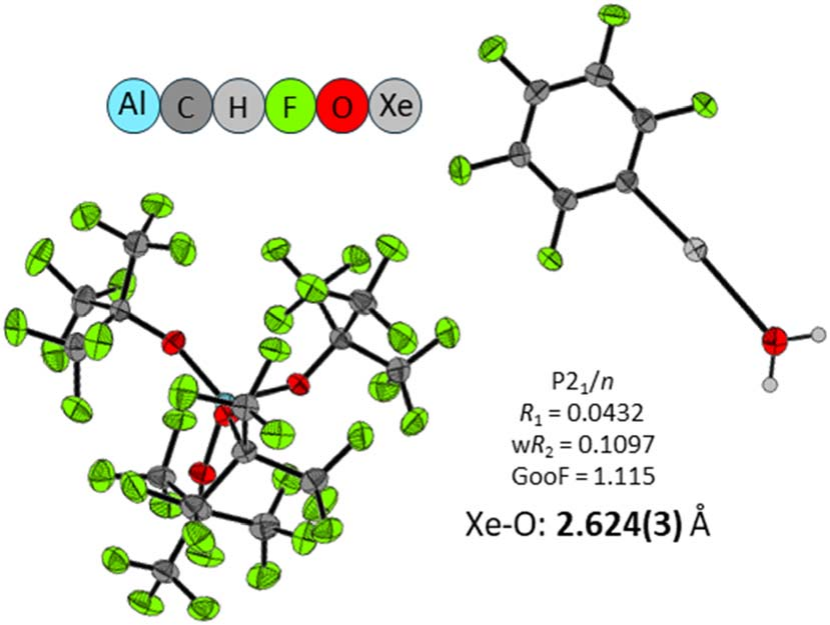
Molecular structures of [C_6_F_5_Xe(H_2_O)][Al(OR^F^)_4_]. Selected bond length: Xe⋯O(H_2_O) = 2.624(3) Å. Thermal displacement ellipsoids are shown at the 50% probability level.

During these investigations, crystals of [C_6_F_5_Xe(OH_2_)][Al(OR^F^)_4_] were obtained accidentally when Li[Al(OR^F^)_4_] was used instead of Me_3_SiF–Al(OR^F^)_3_ (SI 8.3). While characterized only by scXRD, this species constitutes a rare structural example of a xenonium water complex (Fig. 5). The short Xe⋯O(H_2_O) contact of 2.624(3) Å supports coordination of water to the xenonium center. For comparison, recently characterized Xe(vi) hydrate complexes exhibit similarly short Xe⋯O(H_2_O) contacts, *e.g.* in [(18-crown-6)(H_2_O)XeO_3_]·H_2_O, the corresponding Xe⋯O(H_2_O) distance is 2.702(10) Å. However, the bonding situation in the present organoxenonium species is clearly different from that in XeO_3_.^64^

### Molecular structures

The molecular structure of the xenonium ion [C_6_F_5_Xe]^+^ shows distinct interactions depending on the counterion. With the anion [F-Al(OR^F^)_3_]^−^, the compound crystallizes in the orthorhombic space group *Pbca*, forming a contact ion pair. The shortest Xe⋯F(Al) contact is 2.595 Å, notably shorter than the 2.714 Å observed in [C_6_F_5_Xe][AsF_6_],^53^ indicating a stronger cation–anion interaction in the tetrahedral, monodentate anion [F-Al(OR^F^)_3_]^−^. By contrast, when the least coordinating WCA [µ-F(Al(OR^F^)_3_)_2_]^−^ is employed,^12^ the salt [C_6_F_5_Xe][µ-F{Al(OR^F^)_3_}_2_] crystallizes in the monoclinic space group *P*2_1_/*n*, and the closest Xe⋯F contact increases to 2.981 Å. This expanded distance reflects the much weaker interaction, yielding a nearly “naked” xenonium species that better represents the intrinsic properties of the isolated [C_6_F_5_Xe]^+^ cation. These structural differences demonstrate how the anion's coordination behavior and fluorine bridging capacity modulate cation–anion interactions.

**Fig. 6:**
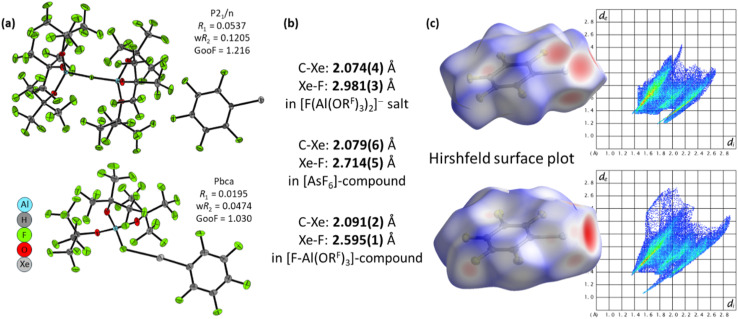
Overview of (a) molecular structures of [C_6_F_5_Xe][F{Al(OR^F^)_3_}_2_] and [C_6_F_5_Xe–µ-F-Al(OR^F^)_3_], (b) selected bond lengths with corresponding values in the [AsF_6_]^−^ compound for reference, and (c) Hirshfeld surface plot illustrating the interaction with the counterion. Thermal displacement ellipsoids are shown at the 50% probability level. Hirshfeld surfaces were calculated and mapped with *d*_norm_ for visualizing close contacts and intermolecular interactions (−0.57 (red)–1.24 (blue)), including their corresponding fingerprint plots.

The Hirshfeld surface analysis of the scXRD structures of [C_6_F_5_Xe][F{Al(OR^F^)_3_}_2_] and C_6_F_5_Xe–F-Al(OR^F^)_3_ clearly demonstrates the stronger interaction of the [FAl(OR^F^)_3_]^−^ anion with the cation (see Fig. 6c). On the Hirshfeld surface, the red regions correspond to contacts that are shorter than the sum of the van der Waals radii, indicating enhanced intermolecular interactions.

### Stability of arene-xenonium ions

**Scheme 4:**
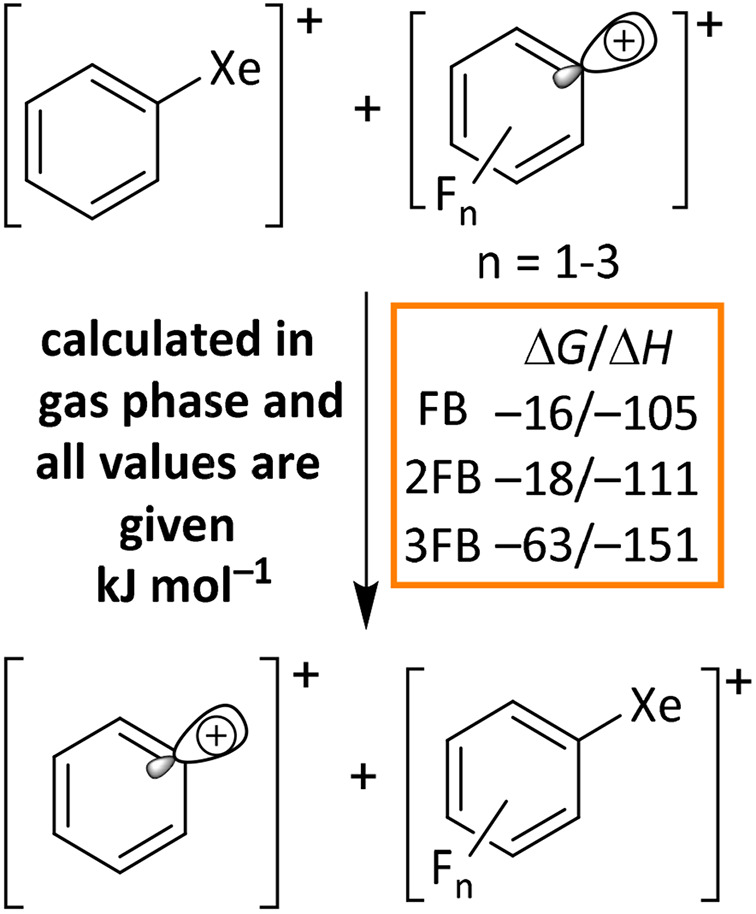
Calculated Δ*G* and Δ*H* values (kJ mol^−1^) for a Xe-atom exchange between [C_6_H_5_Xe]^+^ and fluorinated phenyl cations in the gas phase using the r^2^scan-3c functional with D4 dispersion correction in combination with the def2-mTZVPP basis set.

To revisit the literature statement that xenonium species derived from highly fluorinated arenes exhibit increased stability with rising degrees of fluorination^65^ and to support the stability of the observed 2,3,4-trifluorophenylxenonium ion, this trend was quantified by density functional theory (DFT) calculations (RI-r^2^scan-3c(D4)/def2-mTZVPP) by investigating the isodesmic transfer of a xenon atom from a phenyl xenonium ion onto a fluorinated phenyl cation: Scheme 4 shows that the reaction turns increasingly exergonic as the degree of fluorination increases.

## Proposed mechanism for biphenyl-formation and quantum chemical calculations

**Scheme 5:**
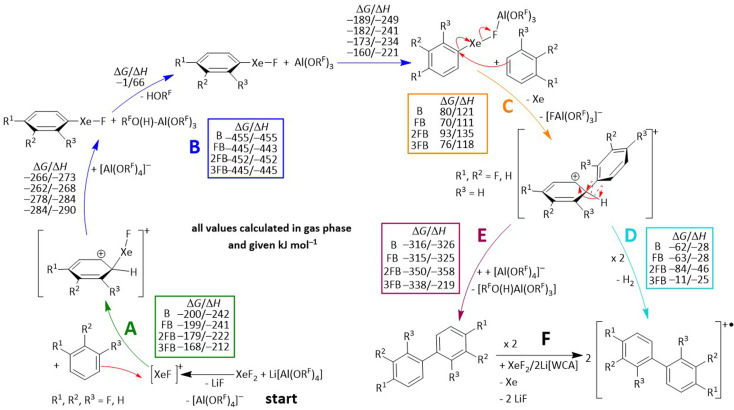
Proposed mechanism of the coupling reaction towards the observed biphenyl derivatives. Quantum chemical calculations for each step were performed for the gas phase using the r^2^scan-3c functional with D4 dispersion correction in combination with the def2-mTZVPP basis set. Reaction steps (A, B, D and E) are highly exergonic, which is consistent with the fast reaction observed at −40 °C. Moderately endergonic step (C) produces two ions from a neutral molecule and hence will profit from the polar reaction medium, presumably tipping it to exergonic reaction progress. For the final step, deprotonation and rearomatization (E) are more likely than formal H-radical and then H_2_ formation (D), which would directly generate the observed radical cation of the coupling product.

With this knowledge in mind, we now turn to the question of what mechanism leads to the observed radical cations of the coupling products [1]˙^+^, [2]˙^+^ and [3]˙^+^. Filler *et al.* proposed that the coupling of arenes induced by XeF_2_ in the presence of HF proceeds *via* a radical mechanism, involving the formation of the phenyl radical as a key intermediate, since no other intermediates were observed that could support an electrophilic mechanism.^66^ However, in the present XeF_2_/2Li[Al(OR^F^)_4_] system, we observe the concomitant formation of Li[FAl(OR^F^)_3_] and HOR^F^, as well as in the low-temperature NMR studies the development of the 2,3,4-trifluorophenylxenonium ion. Therefore, we propose the electrophilic aromatic substitution mechanism shown in Scheme 5, based on experimental evidence that is further supported by DFT calculations in the subsequent section.

### Organoxenonium-ion formation

The hypothetical mechanism begins with the formation of LiF and [XeF][Al(OR^F^)_4_] in a medium that weakly solvates Li^+^. The xenonium species generated then acts as a strong electrophile and forms Wheland intermediates with the arenes benzene, FB, 2FB and 3FB (step A in Scheme 5). The +M-effect of the fluorine substituent accounts for the observed substitution patterns in the products. While formal elimination of HF from this Wheland intermediate could in principle yield the organoxenonium species directly, we presume instead that this intermediate reacts as a very strong Brønsted acid by protonation of one of the oxygen atoms of a second equivalent of the counterion [Al(OR^F^)_4_]^−^ yielding the known, but weakly bound alcohol adduct R^F^O(H)–Al(OR^F^)_3_. The latter dissociates into the corresponding alcohol as well as the known Lewis super acid Al(OR^F^)_3_.^67^ The Lewis super acid then abstracts in step B a fluoride ion from the Xe-compound to probably generate a contact ion pair, related to the independently prepared [C_6_F_5_Xe–µ-F-Al(OR^F^)_3_]. Subsequently, the organoxenonium species acts as a [CH_5−*n*_F_*n*_]^+^ (*n* = 0–3) phenyl cation synthon and undergoes coupling with a second equivalent of the arene, accompanied by liberation of neutral xenon gas, to yield the coupling product in step C, again viewed as being formally a protonated (fluorinated) biphenyl [H(CH_5−*n*_F_*n*_)_2_]^+^ and thus a very strong Brønsted acid. For the final step, two mechanistic pathways are conceivable (D and E). Pathway D would directly lead to the observed radical cation coupling products *via* the formal elimination of a hydrogen atom (resulting in H_2_ evolution). Although this step is calculated to be exergonic, such a reaction is highly unusual. Notably, literature examples exist for protonated arenes, *e.g.* [H(mesitylene)]^+^,^68^ yet in those cases the formation of the corresponding radical cation was never reported. Instead, we assume that the deprotonation of this species, followed by rearomatization, takes place, which yields the neutral coupling products in step E. As Poleschner *et al.* have demonstrated, the combination of XeF_2_ with the Lewis acid Al(OR^F^)_3_ acts as a strongly oxidizing system.^3^ However, the identity of the final oxidant remains ambiguous, as both XeF_2_ in combination with the Lewis acid and Li[Al(OR^F^)_4_] could account for the formation of the observed radical cations of the coupling products in step G.

### DFT calculations

To support the proposed mechanism, we performed DFT calculations to assess the underlying thermodynamics in the gas phase, employing the cost-efficient but accurate D4 dispersion corrected r^2^scan-3c functional with the def2-mTZVPP basis set (Scheme 5).^69^ The first two reaction steps A and B are highly exergonic, which is in line with the experimental observation that the reactions even take place at −40 °C. Step C is moderately endergonic for all calculated arenes. Yet, this unfavorable thermodynamics is probably caused by the formation of two charged species from a neutral molecule. Bond heterolysis will be favored by polar solvents and should also be favorable in solution. By comparing steps D and E, the deprotonation and rearomatization are clearly thermodynamically favored, which underlines the assumption of forming the neutral coupling product followed by oxidation of another equivalent of XeF_2_/2Li[Al(OR^F^)_4_]. For benchmarking the protonation steps B and D, we also calculated the reaction [H(mesitylene)]^+^ + [Al(OR^F^)_4_]^−^ → mesitylene + R^F^O(H)–Al(OR^F^)_3_, which exhibits gaseous Δ*G*/Δ*H* values of −216/–223 kJ mol^−1^ (RI-r^2^scan-3c(D4)/def2-mTZVPP). Note that [H(mesitylene)]^+^[Al(OR^F^)_4_]^−^ is experimentally known to be stable in DCM solution at −20 °C, but decomposes slowly over hours at RT.^67^ This comparison is particularly relevant, since both reactants are charged and the products are neutral, like steps B and D in the proposed mechanism. However, both steps are considerably more exergonic than this reference reaction, further supporting the conclusion that anion protonation is also favorable in solution.

Overall, experiments and calculations support the conclusion that the XeF_2_/2Li[Al(OR^F^)_4_] system mediates the coupling of arenes, followed by oxidation of the resulting biphenyl derivatives.

## Conclusion

Through the combination of XeF_2_ and Li[Al(OR^F^)_4_] in polar but almost non-coordinating fluorobenzene solvents, activation of XeF_2_ was achieved at low temperature. With benzene (dissolved in 2FB), FB and 2FB, this led to coupling reactions forming the biphenyl derivatives 1–3 and their oxidation to the corresponding radical cations. A weakly solvated Li^+^ source is essential, as Li[SbF_6_] showed no reactivity under analogous conditions and acetonitrile blocked reactions in separate experiments. The observation of the 2,3,4-trifluorophenylxenonium ion using 3FB suggests an electrophilic mechanism, which was further explored through DFT studies. The successful synthesis of compounds 4 and 5 demonstrates the compatibility of organoxenonium species with alkoxy aluminates as anions. Stability studies of XeF_2_ at room temperature revealed that it remains stable for 2 h in 5FB before fluorination of 5FB occurs, whereas in acetonitrile XeF_2_ is stable for several days.

By choosing suitable reagents, we showed that the XeF_2_/2Li^+^ system can react as an oxidant in suitable and compatible solvents. Upon reaction with anthracene^Hal^ in 5FB, the characteristic intensely green colored solutions of [anthracene^Hal^]˙^+^ were formed, indicating a redox potential of the system of at least 1.42 V *vs.* Fc^+/0^ in 5FB. In contrast, no oxidation of anthracene^Hal^ was observed in MeCN. Control experiments further showed that Fc can already be oxidized by XeF_2_ alone, whereas addition of Li[Al(OR^F^)_4_] affords [Fc][Al(OR^F^)_4_]. Thus, in acetonitrile, Li[Al(OR^F^)_4_] appears to mainly act as a metathesis reagent and yields a stable counterion for the ferrocenium cation. This led to the conclusion that the “price” for the higher stability of XeF_2_ in acetonitrile is the well solvated and hence less active Li^+^ in solution.

In summary, the XeF_2_/2Li[Al(OR^F^)_4_] system exhibits high reactivity even at low temperatures but is accompanied by challenges due to side reactions. In 5FB, it enables access to high potentials up to 1.42 V and potentially even higher, whereas acetonitrile offers better XeF_2_ stability but suppresses the enhanced reactivity of the combined XeF_2_/2Li^+^ system. Consequently, side reactions such as arene fluorination or coupling reactions and restricted solvent choice (favoring 5FB) represent key limitations. The isolated xenonium salts expand the field of xenon chemistry by demonstrating that readily available alkoxyaluminates can serve as anions. In addition, the observation of [C_6_F_5_Xe(OH_2_)][Al(OR^F^)_4_] provides a rare example of a xenonium water complex. The observed radical cations of fluorinated biphenyls exhibit significant potential as strong oxidants and are the subject of intense ongoing studies.

## Author contributions

KK performed the majority of the syntheses and characterizations, carried out the DFT calculations and analyses, and co-wrote the manuscript together with IK. TH synthesized the [C_6_F_5_Xe][WCA] species together with MS. BL investigated the coupling products under the supervision of KK. TS synthesized the [C_6_F_5_Xe(OH_2_)]^+^ species under the supervision of KK. PT and SR performed the EPR measurements and simulations, and wrote and reviewed the corresponding section of the manuscript. IK conceived and supervised the project.

## Conflicts of interest

The authors declare no conflict of interest.

## Supplementary Material

SC-017-D6SC01402J-s001

SC-017-D6SC01402J-s002

## Data Availability

CCDC 2529884–2529886 and 2531335 contain the supplementary crystallographic data for this paper.^70*a*–*d*^ Experimental details, procedures, weights, and 1D- and 2D-NMR spectra of the reactions are provided in the supplementary information (SI). Supplementary information: the general synthetic methods and characterization techniques used for this work together with the experimental procedures. Additional figures such as NMR, IR and EPR spectra are presented, as well as the crystallographic data of the isolated salts and details of the quantum chemical calculations. The authors have cited additional references within the SI. See DOI: https://doi.org/10.1039/d6sc01402j.
