# Supplementary material for: Using xenon difluoride and 2 Li[Al{OC(CF3)3}4] as an oxidant: from organoxenonium intermediates to (fluoro-)biphenyl radical cations
Source: Chem Sci. 2026 May 5;17(25):12385–93. doi: 10.1039/d6sc01402j (PMC13174956; doi:10.1039/d6sc01402j)
Supplement: SC-017-D6SC01402J-s001 [file SC-017-D6SC01402J-s001.pdf]

Supplementary Information (SI) for Chemical Science.
This journal is © The Royal Society of Chemistry 2026

Electronic Supplementary Information

Using xenon difluoride and 2 Li[Al{OC(CF_3)$_3$}$_4$] as an oxidant: from organoxenonium intermediates to (fluoro-)biphenyl radical cations

Konstantin Kloiber,[a] Tim Heizmann,[a] Benoît Lacombe,[a] Philipp Thielert,[b,c] Malte Sellin,[a] Tim Schwandt,[a] Sabine Richert,[b,c] Stefan Weber[b] and Ingo Krossing[a]*

[a] M.Sc. Chem. Konstantin Kloiber, M.Sc. Chem. Tim Heizmann, B.Sc. Chem. Benoît Lacombe, and Prof. Dr. Ingo Krossing, Institute of Inorganic and Analytical Chemistry and Freiburg Materials Research Center (FMF), University of Freiburg, Albertstr. 21, 79104 Freiburg, Germany.
[b] M.Sc. Chem. Philipp Thielert, Prof. Dr. Sabine Richert and Prof. Dr. Stefan Weber, Institute of Physical Chemistry, University of Freiburg, Albertstr. 21, 79104 Freiburg, Germany.
[c] M.Sc. Chem. Philipp Thielert, Prof. Dr. Sabine Richert, Institute of Physical Chemistry II, University of Ulm, Lise-Meitner-Str. 16, 89081 Ulm, Germany.

* Correspondence to krossing@uni-freiburg.de.

1. Compounds

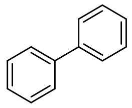

1
154.21 g mol^{-1}

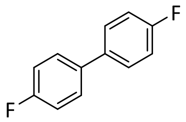

2
190.19 g mol^{-1}

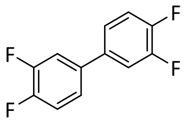

3
226.17 g mol^{-1}

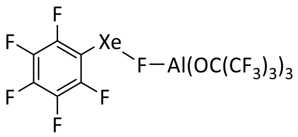

4
1049.35 g mol^{-1}

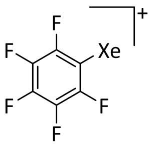

[F(Al(OC(CF_3)$_3$)$_3$)$_2$]$^-$
5
1781.48 g mol^{-1}

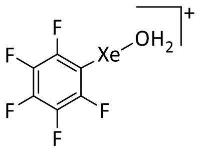

[Al(OC(CF_3)$_3$)$_4$]$^-$
6
1283.44 g mol^{-1}

2. General Remarks

Due to the high oxidation and hydrolysis sensitivity of the reagents, all work was carried out using standard Schlenk technique or in a glove box (MBraun, filled with argon (O_2/H_2O < 1 ppm)). All glassware used in reactions were stored overnight in an oven at 130 °C or dried under reduced pressure (10^{-3} mbar) by using a heat gun. Some reactions were performed in a special double Schlenk vessel. This vessel consists of two Schlenk tubes connected by a G3/G4 frit and equipped with grease-free PTFE or glass valves (see Figure S 1).

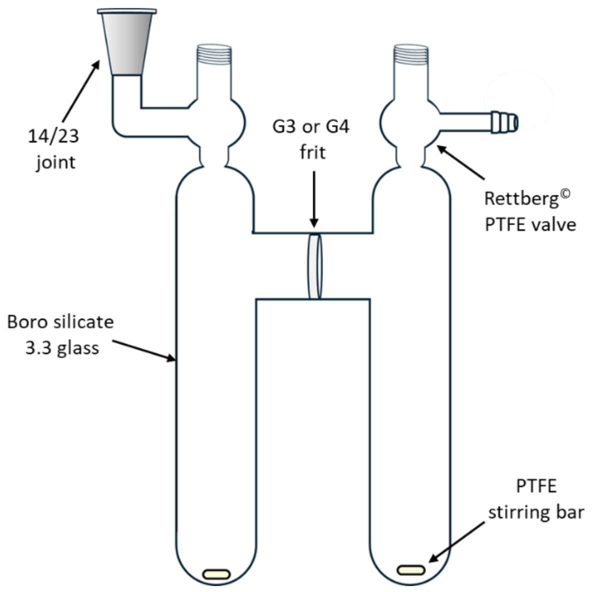

Figure S 1: Sketch of a standard double Schlenk vessel.

Li[Al(OR^F)$_4$][1] and Me_3SiF-Al(OR^F)$_3$[2] were prepared according to literature protocols. The fluorinated benzene derivatives were purchased from Apollo Scientific Ltd and stirred over CaH_2 for three days. They were then condensed onto activated molecular sieves (3 Å). To remove traces of less fluorinated arenes from the more highly fluorinated arenes, Ag[Al(OR^F)$_4$] was added prior to distillation, as the less fluorinated arenes (FB, 2FB and 3FB) form stable arene complexes with the silver salt. After this purification step, the purified fluorinated benzene derivative was condensed into a new vessel.

$B(C_6F_5)_3$ and XeF_2 were available from in-house stocks, and their identity and purity were confirmed by NMR spectroscopy.

2.1. Single Crystal X-Ray Diffraction

Crystals were obtained by laying the reaction solution with *n*-pentane, slowly evaporation of the solvent in a glove box or by concentrating the reaction solution and storing it at low temperature. Single crystal X-ray diffraction data were collected from shock-cooled single crystals at 100(2) K on a Bruker D8 VENTURE three-circle diffractometer with a microfocus sealed X-ray tube using mirror optics as monochromator and a Bruker PHOTON III detector. All crystals were selected under perfluoropolyether oil (PFPE, JC 1800), mounted on 0.1 to 0.2 mm diameter CryoLoops quench-cooled using an *Oxford Cryostream 800* open flow N_2 cooling device.[3] Data were collected at 100 K using monochromated Mo K_α radiation (λ = 0.71073 Å).

All data were integrated with SAINT and a multi-scan absorption correction using SADABS was applied.[4,5] The structure was solved by direct methods using SHELXT and refined by full-matrix least-squares methods against F^2 by SHELXL-2019/1[6] employing shelXle.[7] All non-hydrogen atoms were refined with anisotropic displacement parameters. The hydrogen atoms were refined isotropically on calculated positions using a riding model with their U_{iso} values constrained to 1.5 times the U_{eq} of their pivot atoms for terminal sp^3 carbon atoms and 1.2

times for all other carbon atoms. Disordered moieties were refined using bond lengths restraints and displacement parameter restraints. Some parts of the disorder model were introduced by the program DSR.[8] Graphical representations were prepared using Olex2-1.5.[9]

Crystallographic data for the structure reported in this paper have been deposited at the Cambridge Crystallographic Data Centre.[10] CCDC contains the supplementary crystallographic data for this paper. Copies of the data can be obtained free of charge from the Cambridge Crystallographic Data Centre via www.ccdc.cam.ac.uk/structures. This report and the CIF file were generated using FinalCif.[11]

2.2. Hirshfeld Surface Plot

The Hirshfeld surface plots of $[C_6F_5Xe][F(Al(OR^F)_3)_2]$ and $[C_6F_5Xe\text{-}F\text{-}Al(OR^F)_3]$ were generated by using the free software CrystalExplorer.[12]

2.3. NMR Spectroscopy

^{1}H-, ^{19}F-, ^{27}Al-, ^{129}Xe- and 2D-NMR spectra were collected on an *Avance DPX 200* (200 MHz), *Avance III HD* (300 MHz) and *Avance II+ 400* (400 MHz) NMR spectrometers from *Bruker*. NMR spectra were analyzed using *Bruker TopSpin 4.1.4*. Chemical shifts in ^{1}H-NMR spectra are referenced to $SiMe_4$. ^{1}H-NMR spectra were also calibrated using the solvent signal as an internal reference. Chemical shifts for FB (^{1}H: δ = 6.88 ppm), 2FB (^{1}H: δ = 6.96 ppm), 3FB (^{1}H: δ = 6.86 ppm) and 5FB (^{1}H: δ = 7.01 ppm) were determined experimentally by adding $SiMe_4$ to the aromatic solvent. In FB, one of the two most intense signals of the downfield multiplet appears at δ = 6.88 ppm; in 2FB, the most intense signal appears at δ = 6.96 ppm; in 3FB, one of the two most intense signals of the downfield multiplet appears at δ = 6.86 ppm; and in 5FB, one of the two most intense signals of the downfield multiplet appears at δ = 7.01 ppm, with the $SiMe_4$ signal referenced at 0.00 ppm (Figure S 2 – 5).

Heteroatomic spectra were calibrated according to the IUPAC χ-table.[13] ^{19}F chemical shifts were given with respect to $CFCl_3$, ^{27}Al chemical shifts with respect to 1.1 M $Al(NO_3)_3$ in D_2O and ^{129}Xe chemical shifts with respect to $XeOF_4$ (neat liquid).

The ^{19}F and ^{27}Al NMR signals of the $[Al(OR^F)_4]^-$ anion appear at –75 ppm and 35 ppm, respectively. The broad resonance at ca. 60 ppm in the ^{27}Al NMR spectra is caused by the probe head.

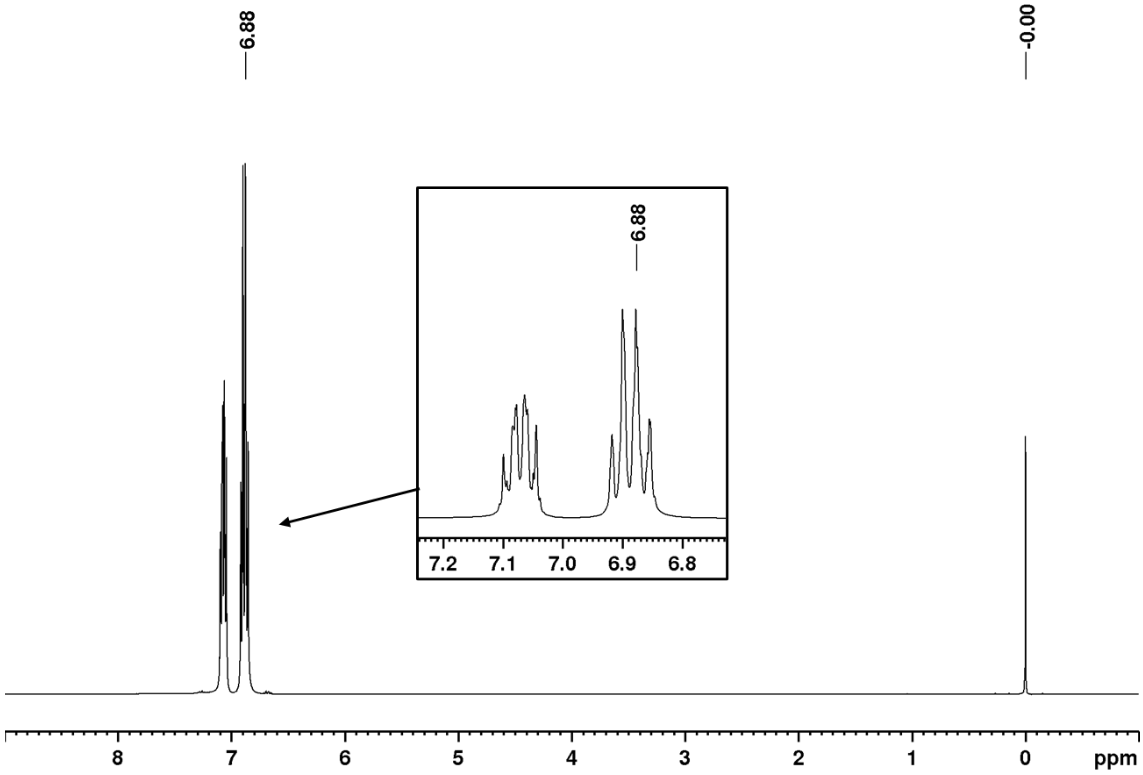

Figure S 2: ^{1}H NMR spectrum of FB with $SiMe_4$ as internal reference (400.17 MHz, FB, 298 K).

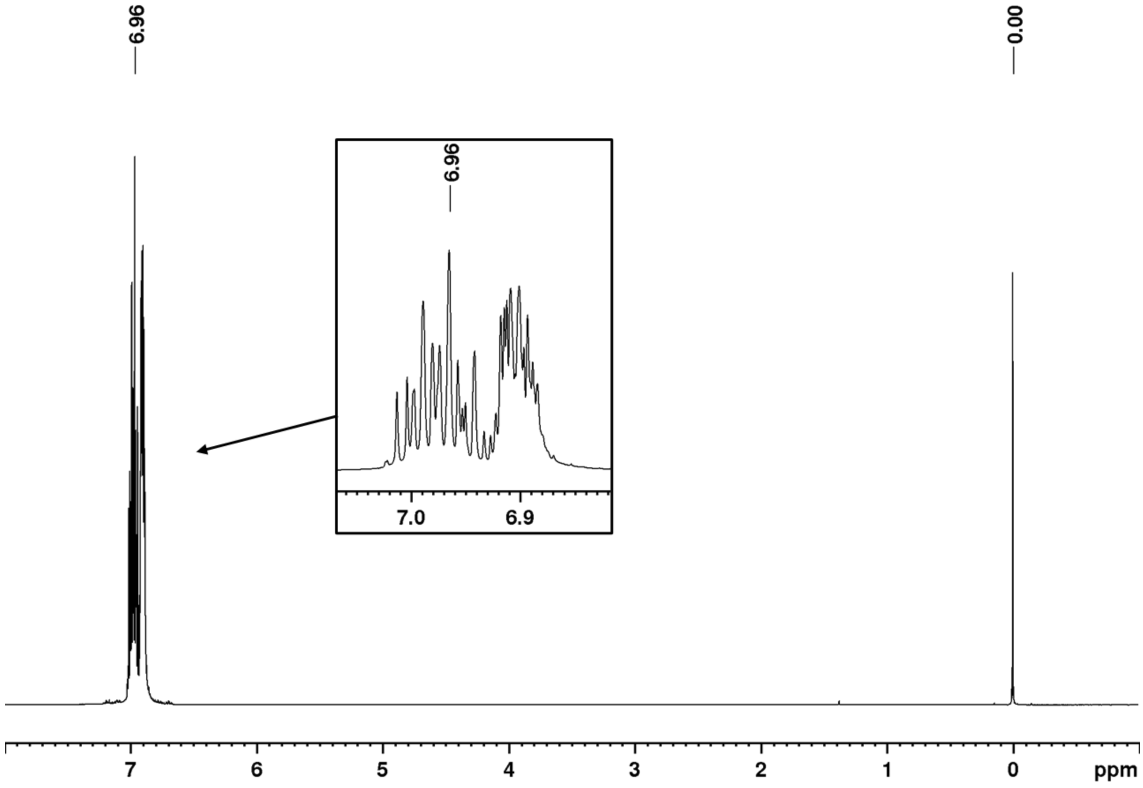

Figure S 3: ^{1}H NMR spectrum of 2FB with $SiMe_4$ as internal reference (400.17 MHz, 2FB, 298 K).

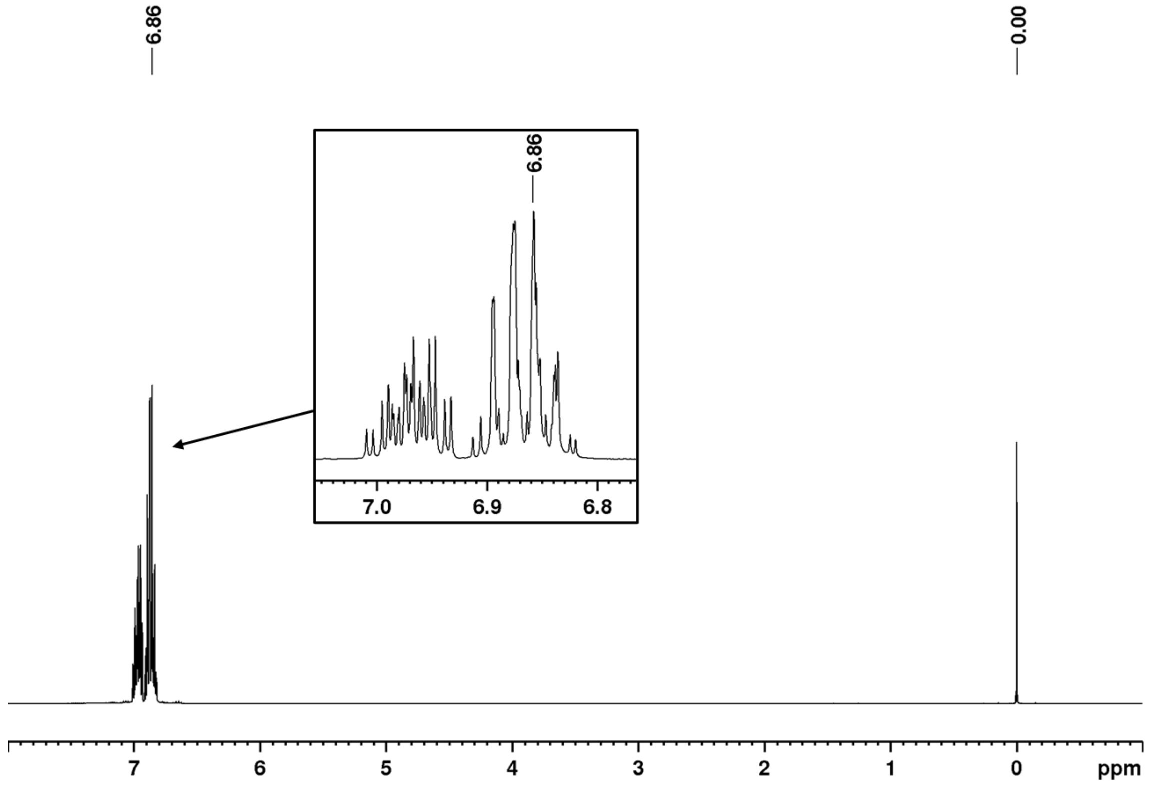

Figure S 4: ^{1}H NMR spectrum of 3FB with $SiMe_4$ as internal reference (400.17 MHz, 3FB, 298 K).

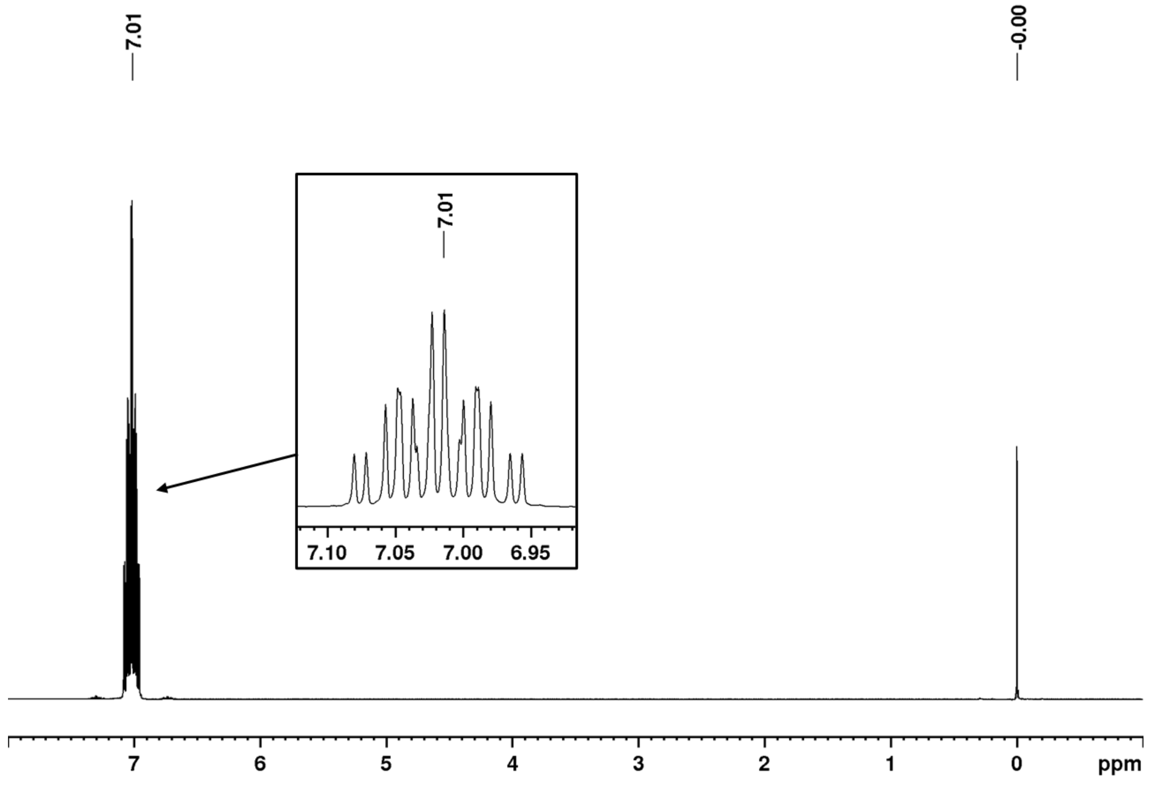

Figure S 5: ^{1}H NMR spectrum of 5FB with $SiMe_4$ as internal reference (400.17 MHz, 5FB, 298 K).

2.4. EPR Spectroscopy

Continuous wave (cw) EPR spectra of the reaction solutions obtained from XeF_2/2Li[Al(OR^F)$_4$] with benzene, fluorobenzene or 1,2-difluorobenzene, containing the radical cations **[1]**$^{\cdot+}$, **[2]**$^{\cdot+}$ and **[3]**$^{\cdot+}$ were recorded at the X-band (9.75 GHz) on a Bruker EMXnano benchtop EPR spectrometer at room temperature. The modulation frequency was set to 100 kHz and the modulation amplitude to 0.1 mT ([1]$^{\cdot+}$), 0.03 mT ([2]$^{\cdot+}$) or 0.2 mT ([2]$^{\cdot+}$ and [3]$^{\cdot+}$) at a microwave power of 1.000 mW (20 dB, [1]$^{\cdot+}$), 0.2512 mW (26 dB, [2]$^{\cdot+}$) or 1.000 mW (20 dB, [3]$^{\cdot+}$). After data acquisition, the spectra were baseline-corrected, frequency-corrected to 9.75 GHz and field-corrected using a carbon fiber standard with g = 2.002644.[14] The numerical simulation and fitting of the experimental spectrum was performed using EasySpin[15] functions garlic and esfit in MATLAB.

2.5. IR Spectroscopy

FT-IR spectra were recorded on a *FT-IR Bruker Alpha*, equipped with a *Quick Snap Platinum ATR* (diamond or ZnSe crystal) unit inside a glove box under an atmosphere of nitrogen at rt. The spectra were recorded with a resolution of 2 cm^{-1} and 64 scans. For measurements and data processing, the software *OPUS 7.5* (Bruker Optic GmbH) was employed. All spectra were ATR corrected, and a base line correction (5 cycles) were employed. By setting the most intense peak to 100 %, the other IR bands were given the following intensity assignment: very weak (vw) < 20 %, weak (w) < 40 %; medium (m) < 60 %, strong (s) < 80 % and very strong (vs) for ≥ 80 %.

2.6. Quantum Chemical Calculations

Geometry optimizations were performed for standard conditions (T = 298 K and p = 1.00 atm) with ORCA 5.0[16] using the cost-efficient r2SCAN-3c[17] density functional and the def2-mTZVPP[17] basis set. These calculations employed geometric counterpoise correction (gCP)[18] and the atom-pairwise D4 dispersion correction[19,20], which utilizes tight-binding partial charges.

The Gibbs free energy can be calculated using Equation S1.

$$G^0 = H^0 - T \cdot S^0 \qquad \text{(S 1)}$$

$$H^0 = E_{\mathrm{el}} + E_{\mathrm{vrt}} + \mathrm{R} \cdot T \qquad \text{(S 2)}$$

$$S = \mathrm{k}_B N \left[\ln\left(\frac{V}{N\Lambda^3}\right) + \frac{5}{2} \right] \qquad \text{(S 3)}$$

$$\Lambda = \frac{h}{\sqrt{2\pi m \mathrm{k_B} T}}$$

For atoms in the gas phase, the ORCA output does not provide the Gibbs free energy G^0 directly. In these cases, G^0 was calculated using Equations S1 to S3. In Equation S 2, E_{el} represents the electronic energy, corresponding to the final single-point energy reported in the ORCA output. The term E_{vrt} is the sum of the translation, rotation and vibrational energies including zero-point energy. Single atoms contribute only translation energy, resulting in $E_{vrt} = \frac{3}{2}RT$. Equation S 3 represents the Sackur-Tetrode Equation[21], which contains Planck's constant *h*, Avogadro's number N, the volume *V* and the temperature *T*.

3. Stability of XeF_2 in 5FB and MeCN

3.1. XeF_2 in 5FB

XeF_2 (25.5 mg, 151 µmol) was dissolved in 5FB at room temperature. The solution was directly analyzed by NMR spectroscopy.

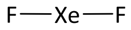

NMR

^{19}F NMR [282.45 MHz, 5FB, 298 K]: δ = –179.5 (s, 2 F, Xe–F) ppm.

^{129}Xe NMR [83.48 MHz, 5FB, 298 K, calibrated to $XeOF_4$]: δ = –1933.9 (t, 1 Xe, Xe, $^{1}J_{Xe,F}$ = 5634 Hz) ppm.

XeF_2 is initially stable in 5FB at room temperature for about one hour. After that time, a second XeF_2 resonance appears in the ^{19}F NMR spectrum very close to the first, suggesting that XeF_2 begins to experience two chemical environments, likely due to weak interactions or partial complex formation with the solvent. By the following day, XeF_2 signal disappears completely, and multiple new fluorine resonances are observed (see ^{19}F NMR spectra). Additionally, SiF_4 is detected at –1246 ppm in the ^{19}F NMR, which is a typical reaction of XeF_2 with silicate glass. These observations demonstrate that XeF_2 is stable for 2 h at room temperature in 5FB.

3.2. XeF_2 in Acetonitrile

XeF_2 (25.5 mg, 151 µmol) was dissolved in MeCN at room temperature. The solution was analyzed directly by NMR spectroscopy.

NMR

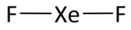

^{19}F NMR [376.54 MHz, MeCN, 298 K]: δ = –180.0 (s, 2 F, ^{129}Xe–F) ppm.

^{129}Xe NMR [111.29 MHz, MeCN, 298 K, calibrated to $XeOF_4$]: δ = –1933.9 (t, 1 Xe, Xe, $^{1}J_{Xe,F}$ = 5642 Hz) ppm.

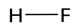

^{1}H NMR [400.18 MHz, MeCN, 298 K]: δ = 6.66 (d, 1 H, **H**–F, $^{1}J_{H,F}$ = 482.6 Hz) ppm.

^{19}F NMR [376.54 MHz, MeCN, 298 K]: δ = –183.7 (d, 1 F, H–**F**, $^{1}J_{H,F}$ = 482.6 Hz) ppm.

XeF_2 shows good stability in acetonitrile for several days under the herein used conditions, which is consistent with previous literature reports.[22] After three days, small amounts of HF could be observed at 6.66 ppm in the ^{1}H NMR spectrum and at –183.7 ppm in the ^{19}F NMR spectrum, likely resulting from reaction with the glass vessel.

4. Syntheses

4.1. Biphenyl – Reaction of XeF_2/2Li[Al(OR^F)$_4$] with Benzene

Li[Al{OC(CF_3)$_3$}$_4$] (150 mg, 154 µmol) was dissolved in benzene/2FB (0.5 mL/3.0 mL) and cooled to – 40 °C. XeF_2 (10.1 mg, 77.0 µmol, 0.50 equiv.) was added, whereupon the reaction mixture turned yellow. The mixture was stirred for 3 h and allowed to warm to room temperature. The reaction was quenched by the addition of water (0.5 mL) and stirring under atmospheric conditions. The resulting mixture was extracted with dichloromethane (3 x 3 mL). The combined organic layers were dried over Na_2SO_4, and the solvent was removed under reduced pressure. Purification by column chromatography using *n*-pentane as eluent afforded the title compound (4.21 mg, 27.3 µmol, 35 %*) as a white solid.

* The yield was calculated based on the proposed reaction mechanism, according to which one equivalent of XeF_2 is required for formation of the coupling product and a second equivalent oxidizes the biphenyl (derivative) to the corresponding radical cation.

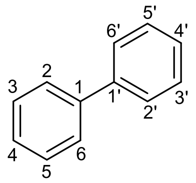

NMR

^{1}H NMR [400.17 MHz, $CDCl_3$, 298 K]: δ = 7.60 (m, 4 H, C^{2}H, $C^{2'}$H, C^{6}H, $C^{6'}$H), 7.44 (m, 4 H, C^{3}H, $C^{3'}$H, C^{5}H, $C^{5'}$H), 7.35 (m, 2 H, C^{4}H, $C^{4'}$H) ppm.

4.2. 4,4’-Difluorobiphenyl – Reaction of XeF_2/2Li[Al(OR^F)$_4$] with Fluorobenzene

Li[Al{OC(CF_3)$_3$}$_4$] (150 mg, 154 µmol) was dissolved in FB (3.0 mL) and cooled to – 40 °C. XeF_2 (10.1 mg, 77.0 µmol, 0.50 equiv.) was added, whereupon the reaction mixture turned blue. The mixture was stirred 3 h and allowed to warm to room temperature. The reaction was quenched by the addition of water (0.5 mL) and stirring under atmospheric conditions. The resulting mixture was extracted with dichloromethane (3 x 3 mL). The

combined organic layers were dried over Na_2SO_4, and the solvent was removed under reduced pressure. Purification by column chromatography using *n*-pentane as eluent afforded the title compound (5.93 mg, 31.2 µmol, 41 %*) as a white solid.

* The yield was calculated based on the proposed reaction mechanism, according to which one equivalent of XeF_2 is required for formation of the coupling product and a second equivalent oxidizes the biphenyl (derivative) to the corresponding radical cation.

NMR

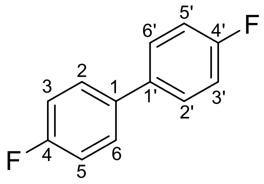

^{1}H NMR [400.17 MHz, $CDCl_3$, 298 K]: δ = 7.49 (m, 4 H, C^2H, $C^{2'}$H, C^6H, $C^{6'}$H), 7.12 (m, 4 H, C^3H, $C^{3'}$H, C^5H, $C^{5'}$H) ppm.

^{19}F NMR [376.5 MHz, $CDCl_3$, 298 K]: δ = –115.8 (m, 2 F, C^4F, $C^{4'}$F) ppm.

4.3. 3,3’,4,4’-Tetrafluorobiphenyl – Reaction of XeF_2/2Li[Al(OR^F)$_4$] with 1,2-Difluorobenzene

Li[Al{OC(CF_3)$_3$}$_4$] (150 mg, 154 µmol) was dissolved in 2FB (3.0 mL) and cooled to – 40 °C. XeF_2 (10.1 mg, 77.0 µmol, 0.50 equiv.) was added, whereupon the reaction mixture turned blue. The mixture was stirred 3 h and allowed to warm to room temperature. The reaction was quenched by the addition of water (0.5 mL) and stirring under atmospheric conditions. The resulting mixture was extracted with dichloromethane (3 x 3 mL). The combined organic layers were dried over Na_2SO_4, and the solvent was removed under reduced pressure. Purification by column chromatography using *n*-pentane as eluent afforded the title compound (7.51 mg, 33.2 µmol, 43 %*) as a white solid.

NMR

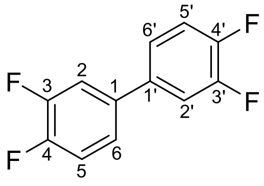

^{1}H NMR [300.18 MHz, $CDCl_3$, 298 K]: δ = 7.34 (m, 2 H, C^2H, $C^{2'}$H), 7.25 (m, 4 H, C^5H, $C^{5'}$H, C^6H, $C^{6'}$H) ppm.

^{19}F NMR [282.45 MHz, $CDCl_3$, 298 K]: δ = –136.9 (m, 2 F, C^3F, $C^{3'}$F), –139.1 (m, 2 F, C^4F, $C^{4'}$F) ppm.

4.4. Reaction of XeF_2/2Li[Al(OR^F)$_4$] with 1,2,3-Trifluorobenzene

Li[Al{OC(CF_3)$_3$}$_4$] (150 mg, 154 µmol) was dissolved in 3FB/5FB (0.5 mL/2.5 mL) and cooled to – 35 °C. XeF_2 (10.1 mg, 77.0 µmol, 0.50 equiv.) was added, whereupon the reaction mixture turned slowly to orange/brown. The reaction mixture was analyzed by NMR at 235 K and rt. The [2,3,4-C_6F_3Xe]$^+$ cation was observed as one of the minor products.

<u>NMR</u>

For clarity, only the xenonium species is discussed in detail. This species was observed as a minor product, while additional signals in the ^{19}F NMR spectrum indicate the presence of further fluorinated aromatic species.

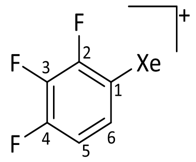

<u>[2,3,4-C_6F_3Xe]$^+$ (minor product)</u>

^{1}H NMR [400.17 MHz, 3FB/5FB (1:4), 243 K]: δ = 8.67 (m, 1 H, C^6H), 8.03 (m, 1 H, C^5H) ppm.

^{19}F NMR [376.53 MHz, 3FB/5FB (1:4), 243 K]: δ = –116.6 (m, 1 F, C^2F), –118.8 (m, 2 F, C^4F), –143.7 (m, 1 F, C^3F) ppm.

^{129}Xe NMR [111.29 MHz, 3FB/5FB (1:4), 243 K]: δ = –3955.7 (m, 1 Xe, C^1Xe) ppm.

^{1}H NMR [400.17 MHz, 3FB/5FB (1:4), 298 K]: δ = 8.59 (m, 1 H, C^6H), 7.99 (m, 1 H, C^5H) ppm.

^{19}F NMR [376.53 MHz, 3FB/5FB (1:4), 298 K]: δ = –116.5 (m, 1 F, C^2F), –118.0 (m, 2 F, C^4F), –143.0 (m, 1 F, C^3F) ppm.

^{129}Xe NMR [111.29 MHz, 3FB/5FB (1:4), 298 K]: δ = –3962.1 (br, 1 Xe, C^1Xe) ppm.

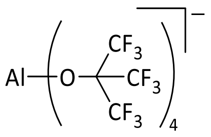

<u>[Al{OC(CF_3)$_3$}$_4$]$^-$</u>

^{19}F NMR [376.53 MHz, 3FB/5FB (1:4), 243 K]: δ = –76.5 (s, 36 F, OC-C**F**$_3$) ppm.

^{27}Al NMR [104.27 MHz, 3FB/5FB (1:4), 243 K]: no signal observed.

^{19}F NMR [376.53 MHz, 3FB/5FB (1:4), 298 K]: δ = –76.2 (s, 36 F, OC-C**F**$_3$) ppm.

^{27}Al NMR [104.27 MHz, 3FB/5FB (1:4), 298 K]: δ = 38.0 (br s, 1 Al, **Al**-O) ppm.

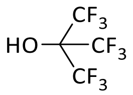

<u>$HOC(CF_3)_3$</u>

^{1}H NMR [400.17 MHz, 3FB/5FB (1:4), 298 K]: δ = 3.66 (s, 1 H, O**H**)

^{19}F NMR [376.53 MHz, 3FB/5FB (1:4), 298 K]: δ = –76.2 (m, 9 F, OC-C**F**3) ppm.

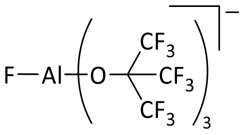

<u>$[FAl\{OC(CF_3)_3\}_3]^-$</u>

^{19}F NMR [376.53 MHz, 3FB/5FB (1:4), 298 K]: δ = –76.8 (s, 27 F, OC-C**F**3) ppm.

^{27}Al NMR [104.27 MHz, 3FB/5FB (1:4), 298 K]: no signal observed (probably under the bs of $[Al\{OC(CF_3)_3\}_4]^-$).

4.5. Reaction of XeF_2/2Li[Al(OR^F)$_4$] with anthracene^{Hal} in 5FB and MeCN

<u>5FB</u>

$Li[Al\{OC(CF_3)_3\}_4]$ (150 mg, 154 µmol) and anthracene^{Hal} (30.1 mg, 77.0 µmol) were dissolved in 5FB (2.5 mL) and cooled to – 35°C. XeF_2 (10.1 mg, 77.0 µmol, 0.50 equiv.) was added and allowed to warm to room temperature, while a color change to deep green took place, consistent with the formation of [anthracene^{Hal}]$^{\cdot+}$.[23]

<u>MeCN</u>

With an analogous procedure in NCMe instead of 5FB no color change to green could be observed, suggesting that formation of [anthracene^{Hal}]$^{\cdot+}$ did not occur under these conditions.

4.6. Reaction of XeF_2/2Li[Al(OR^F)$_4$] with Ferrocene in MeCN

$Li[Al\{OC(CF_3)_3\}_4]$ (150 mg, 154 µmol) and ferrocene (Fc) (14.3 mg, 77.0 µmol, 0.50 equiv.) were dissolved in acetonitrile (2.5 mL) and cooled to –35°C. XeF_2 (10.1 mg, 77.0 µmol, 0.50 equiv.) was added and allowed to warm to room temperature, while a color change to deep blue took place, consistent with the formation of $[Fc]^{\cdot+}$.

Notably, control experiments further showed that Fc can already be oxidized by XeF_2 alone in MeCN, whereas $Li[Al(OR^F)_4]$ primarily acts as a metathesis reagent to afford $Fc[Al(OR^F)_4]$.

4.7. $[C_6F_5Xe][FAl\{OC(CF_3)_3\}_3]$

$B(C_6F_5)_3$ (133 mg, 0.260 mmol) and $Me_3SiF-Al\{OC(CF_3)_3\}_3$ (214 mg, 0.260 mmol, 1.0 equiv.) were placed separately in two arms of a Y-shaped Schlenk tube. Dichloromethane (DCM, 5.0 mL) was added to $B(C_6F_5)_3$ and the mixture was stirred until complete dissolution. The solution was then cooled to –55 °C for 3 h, during which a colorless solid precipitated. The suspension was then cooled to –78 °C and the mother liquor was carefully decanted. DCM (3.3 mL) was added to solid residue at –78 °C, followed by addition of $Me_3SiF-Al(OR^F)_3$ at the same temperature. The reaction mixture was allowed to warm slowly to rt overnight while stirring, yielding a clear dark-brown solution. The mixture was slowly cooled to – 40 °C by storing it in a refrigerator inside a Dewar containing *iso*-propanol. The formed crystals were isolated and characterized by scXRD verified the formation of $[C_6F_5Xe][FAl(OR^F)_3]$. The crystals were dissolved in DCM and characterized by NMR spectroscopy. No yield was determined.

NMR

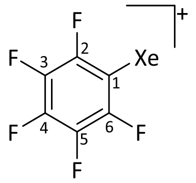

$[C_6F_5Xe]^+$

^{19}F NMR [282.45 MHz, CH_2Cl_2, 298 K]: δ = –123.2 (m, 2 F, C^2F, C^6F), –133.1 (m, 2 F, C^2F, C^6F), –147.8 (m, 1 F, C^4F) ppm.

^{129}Xe NMR [83.48 MHz, CH_2Cl_2, 298 K]: δ = –3948.1 (m, 1 Xe, C^1Xe) ppm.

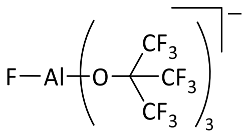

$[FAl\{OC(CF_3)_3\}_3]^-$

^{19}F NMR [282.45 MHz, CH_2Cl_2, 298 K]: δ = –76.0 (br, 27 F, OC-C**F**$_3$), –184.4 (br, 1 F, Al-**F**) ppm.

^{27}Al NMR [78.17 MHz, CH_2Cl_2, 298 K]: δ = –39.3 (br, 1 Al, **Al**-F) ppm.

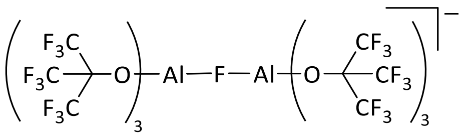

[F{Al(OC(CF$_3$)$_3$)$_3$}$_2$]$^–$

^{19}F NMR [282.45 MHz, CH$_2$Cl$_2$, 298 K]: δ = –76.0 (s, 56 F, OC-C**F**$_3$), –184.9 (br, 1 F, Al-**F**-Al) ppm.

^{27}Al NMR [78.17 MHz, CH$_2$Cl$_2$, 298 K]: δ = no signal observed

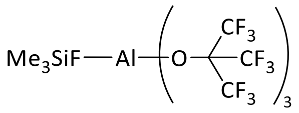

Me$_3$SiF-Al{OC(CF$_3$)$_3$}$_3$

^{1}H NMR [300.18 MHz, CH$_2$Cl$_2$, 298 K]: δ = 0.34 (br, 9 H, Si-C**H**$_3$) ppm.

^{19}F NMR [282.45 MHz, CH$_2$Cl$_2$, 298 K]: δ = –75.9 (s, 56 F, OC-C**F**$_3$), –184.9 (br, 1 F, Si-**F**-Al) ppm.

^{27}Al NMR [78.17 MHz, CH$_2$Cl$_2$, 298 K]: δ = no signal observed

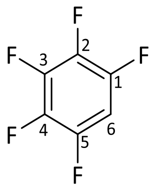

5FB

^{1}H NMR [300.18 MHz, CH$_2$Cl$_2$, 298 K]: δ = 6.93 (m, 1 H, C^{6}H) ppm.

^{19}F NMR [282.45 MHz, CH$_2$Cl$_2$, 298 K]: δ = –139.2 (m, 2 F, C^{1}F, C^{5}F), –154.6 (m, 1 F, C^{3}F), –162.8 (m, 2 F, C^{2}F, C^{4}F) ppm.

scXRD

Pbca, a = 17.8570(18) Å, b = 17.785(4) Å, c = 18.0695(17) Å, α = 90°, β = 90°, γ = 90°, V = 5738.5(15) Å^3, Z = 8

4.8. [C$_6$F$_5$Xe][F(Al(OR^F)$_3$)$_2$]

B(C$_6$F$_5$)$_3$ (267 mg, 0.521 mmol) and Me$_3$SiF-Al{OC(CF$_3$)$_3$}$_3$ (860 mg, 1.04 mmol, 2.0 equiv.) were placed separately in two arms of a Y-shaped Schlenk tube. Dichloromethane (DCM, 15 mL) was added to B(C$_6$F$_5$)$_3$ and the mixture was stirred until complete dissolution. The solution was then cooled to –55 °C, XeF$_2$ (88.3 mg, 0.521 mmol, 1.0 equiv.) was added and stirred for 3 h, during which a colorless solid precipitated. The suspension was then cooled to –78 °C and the mother liquor was carefully decanted. DCM (3.3 mL) was added to solid residue at –78 °C,

followed by addition of Me_3SiF-$Al(OR^F)_3$ at the same temperature. The reaction mixture was stirred for 30 min at – 78 °C and rt. The precipitate was filtered off, and the solution was slowly cooled to – 40 °C by storing it in a refrigerator inside a Dewar containing *iso*-propanol. The formed colorless crystals were isolated and characterized by scXRD and verified as unreacted Me_3SiF-$Al(OR^F)_3$. Yellow-brown crystals were also present but were not suitable for scXRD. The solvent was removed, and the remaining crystals dried in vacuum. Attempts were made to manually separate the colorless crystals from the colored ones. The resulting crystalline mixture was analyzed by IR and NMR spectroscopy. A portion of the crystals were dissolved in 5FB. By slow evaporation of the solvent in the glove box, crystals were formed suitable for scXRD, which verified them as $[C_6F_5Xe][F\{Al(OR^F)_3\}_2]$. The crystals were dissolved in $NCCD_3$ and characterized by NMR spectroscopy. No yield was determined.

NMR

The $[F(Al(OR^F)_2]^-$ anion is not stable in the presence of σ donor ligands, consequently, it decomposes in acetonitrile to give the $[FAl(OR^F)_3]^-$ anion and the stabilized Lewis acid-base adduct MeCN-$Al(OR^F)_3$.

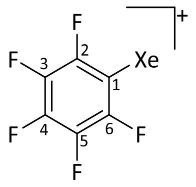

$[C_6F_5Xe]^+$

^{19}F NMR [376.53 MHz, $NCCD_3$, 298 K]: δ = –126.4 (m, 2 F, C^2F, C^6F), –142.6 (m, 2 F, C^2F, C^6F), –155.6 (m, 1 F, C^4F) ppm.

^{129}Xe NMR [111.29 MHz, $NCCD_3$, 298 K]: δ = –3796.3 (m, 1 Xe, C^1Xe) ppm.

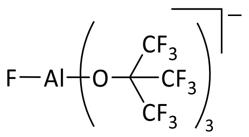

$[FAl\{OC(CF_3)_3\}_3]^-$

^{19}F NMR [376.53 MHz, $NCCD_3$, 298 K]: δ = –76.2 (d, 27 F, OC-C**F**$_3$, $^5J_{F,F}$ = 1.9 Hz), –185.3 (br, 1 F, Al-**F**,) ppm.

^{27}Al NMR [104.27 MHz, $NCCD_3$, 298 K]: δ = –41.4 (br, 1 Al, **Al**-F) ppm.

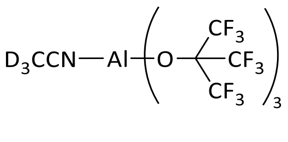

D$_3$CCN-Al{OC(CF$_3$)$_3$}$_3$

^{19}F NMR [376.53 MHz, NCCD$_3$, 298 K]: δ = –75.6 (s, 56 F, OC-C**F**$_3$) ppm.

^{27}Al NMR [104.27 MHz, NCCD$_3$, 298 K]: δ = no signal observed

Me$_3$SiF

Me$_3$SiF

^{1}H NMR [400.17 MHz, NCCD$_3$, 298 K]: δ = 0.24 (d, 9 H, Si-C**H**$_3$, $^3J_{H,F}$ = 7.5 Hz) ppm.

^{19}F NMR [376.53 MHz, NCCD$_3$, 298 K]: δ = –157.6 (s, 1 F, Me$_3$Si**F**, $^3J_{H,F}$ = 7.5 Hz) ppm.

scXRD

$P2_1/n$, a = 13.2484(7) Å, b = 16.7851(11) Å, c = 44.788(3) Å, α = 90°, β = 95.969°, γ = 90°, V = 9905.8(10) Å^3, Z = 8

5. NMR Spectroscopy

5.1. XeF$_2$ in 5FB

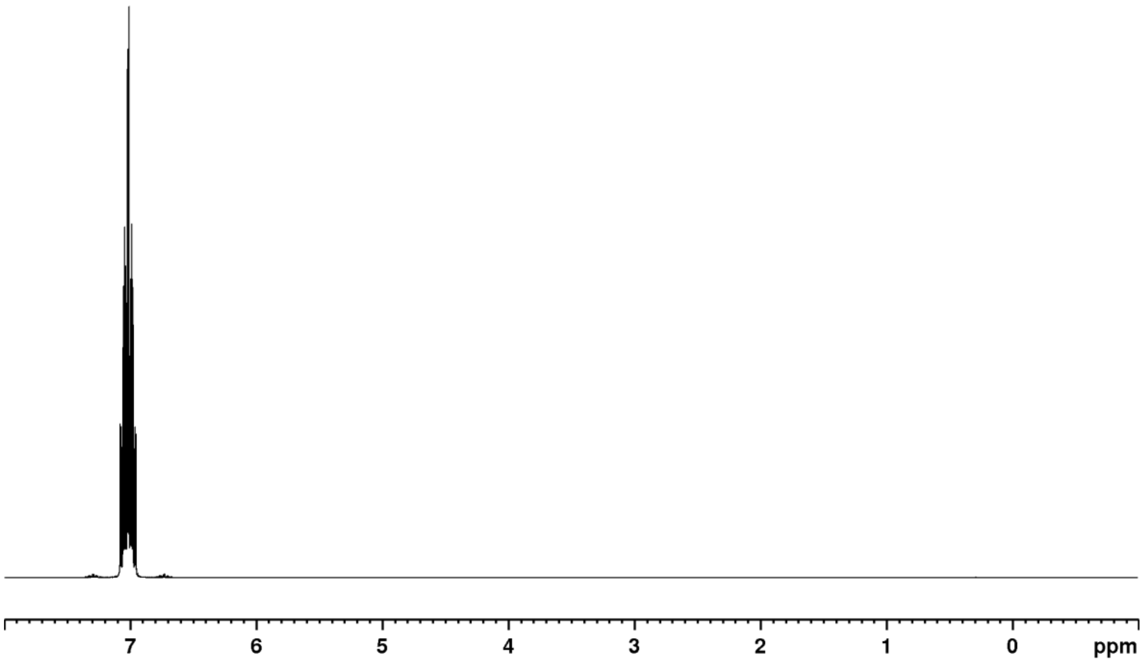

Figure S 6: ^{1}H NMR spectrum of XeF$_2$ in 5FB (300.18 MHz, 5FB, 298 K).

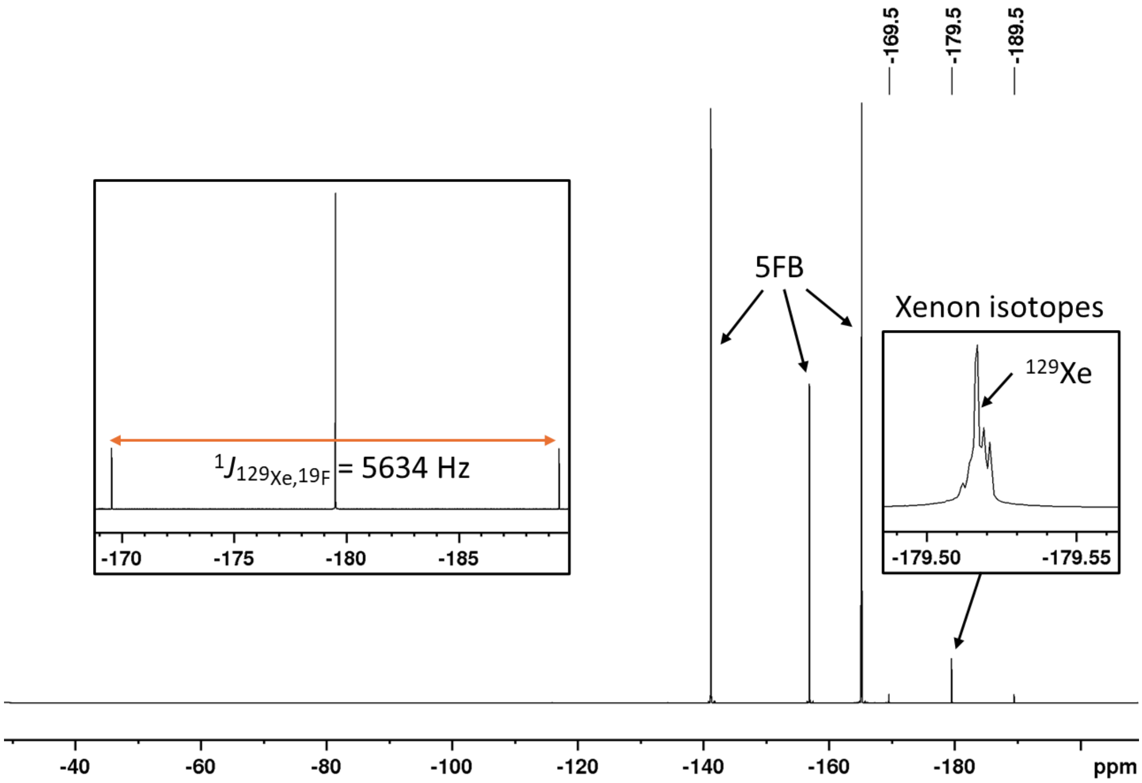

Figure S 7: ^{19}F NMR spectrum directly after dissolving XeF_2 in 5FB (282.45 MHz, 5FB, 298 K).

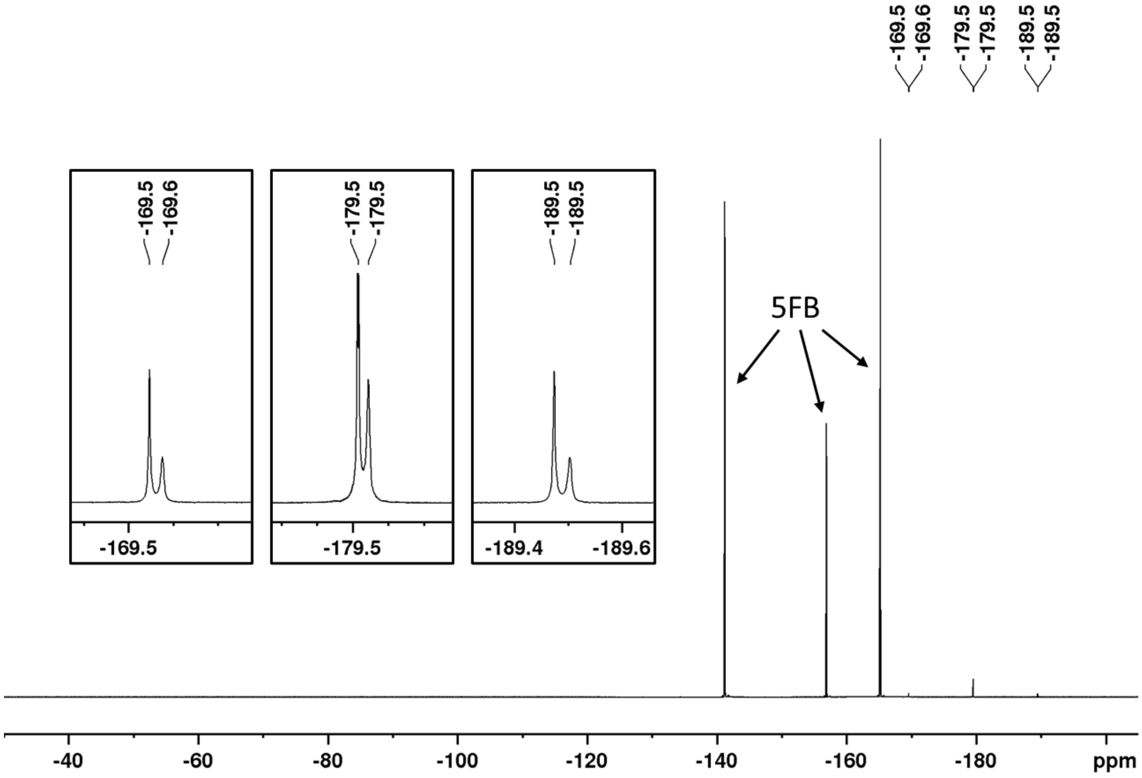

Figure S 8: ^{19}F NMR spectrum of XeF_2 in 5FB after 2h (282.45 MHz, 5FB, 298 K).

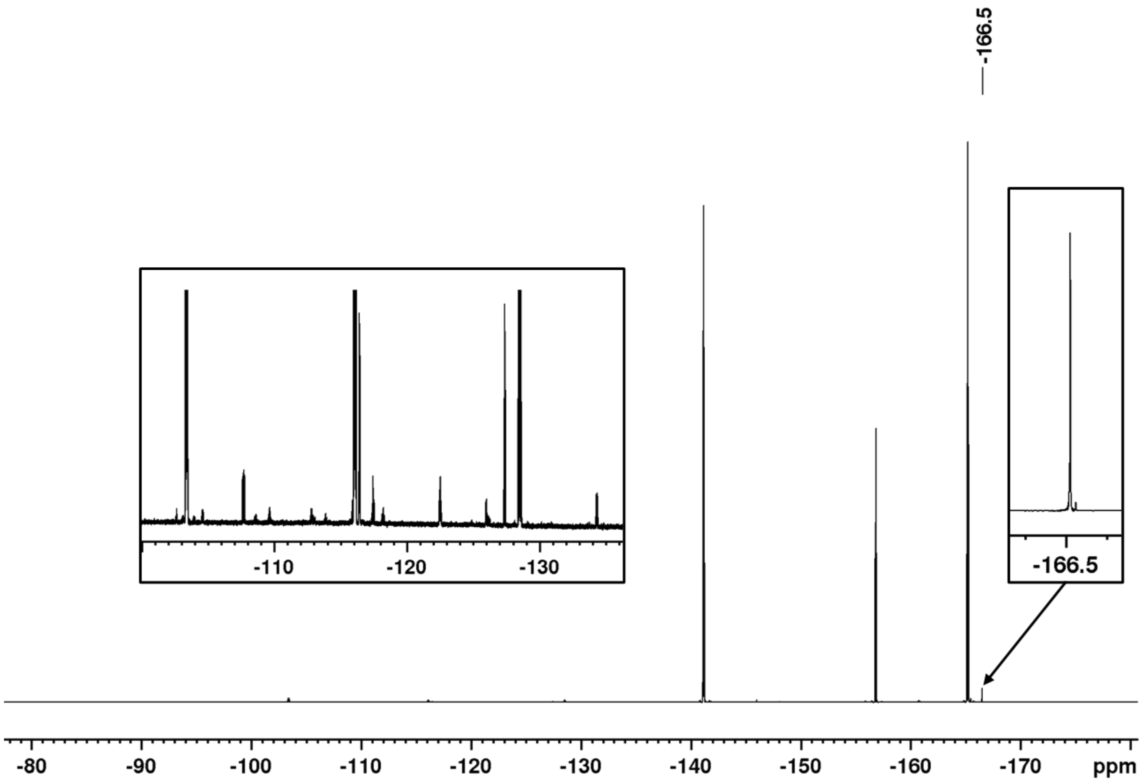

Figure S 9: ^{19}F NMR spectrum of XeF_2 in 5FB after 1d (282.45 MHz, 5FB, 298 K).

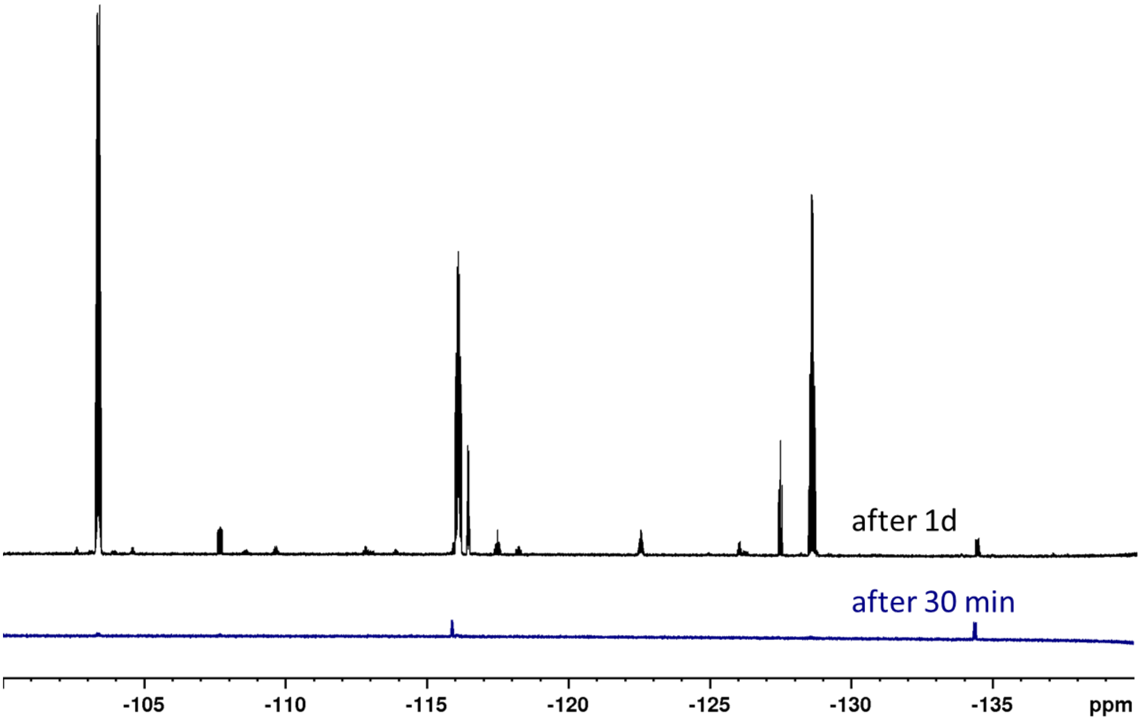

Figure S 10: ^{19}F NMR spectrum of XeF_2 in 5FB after 30 min (blue) and 1d (black) (282.45 MHz, 5FB, 298 K).

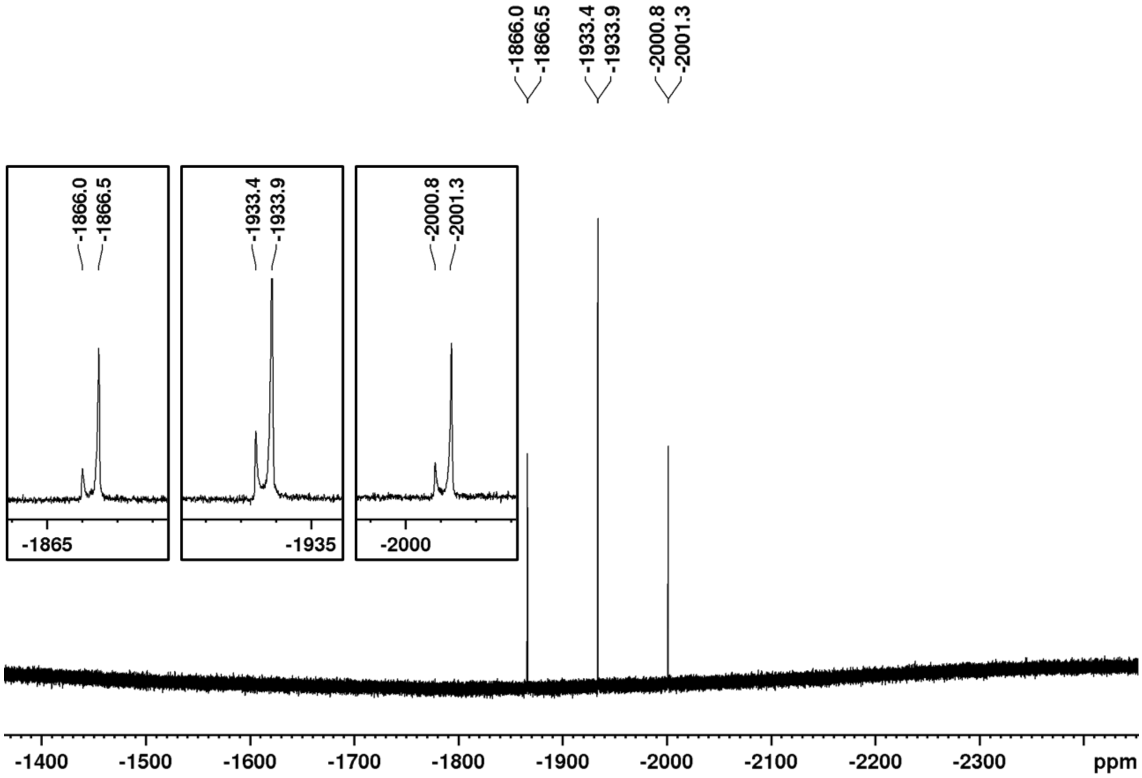

Figure S 11: ^{129}Xe NMR spectrum of XeF_2 in 5FB after 1h (83.48 MHz, 5FB, 298 K, calibrated to $XeOF_4$).

5.2. XeF_2 in Acetonitrile

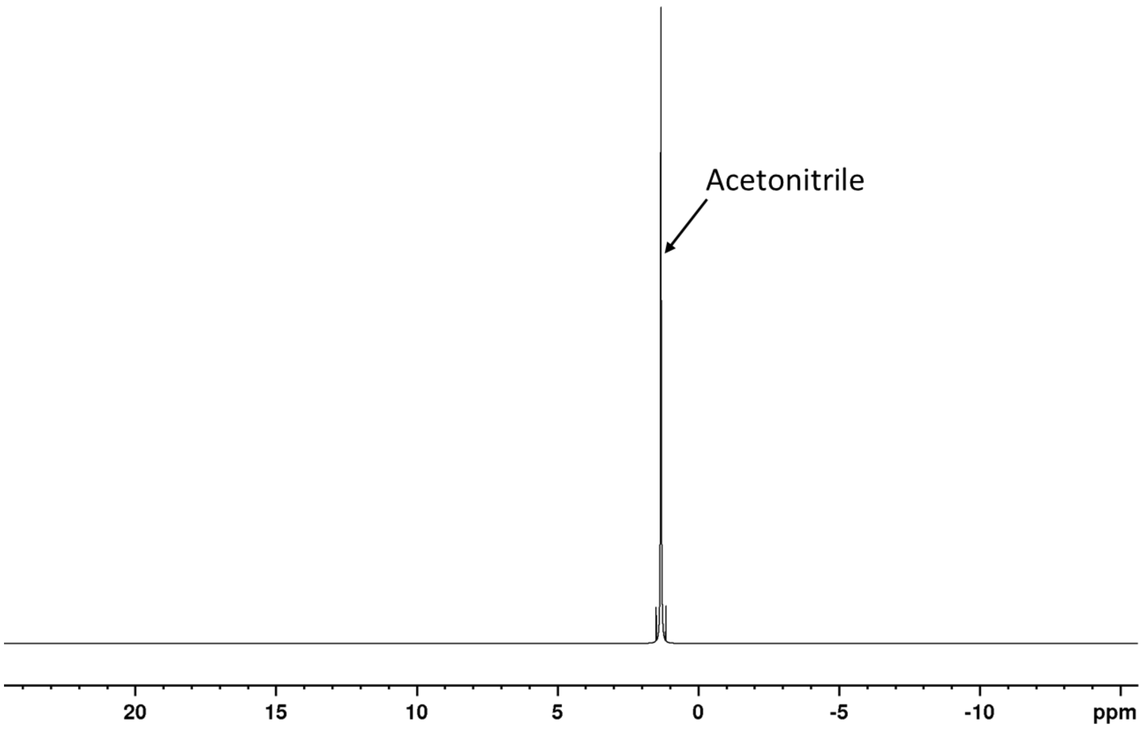

Figure S 12: ^{1}H NMR spectrum of XeF_2 in MeCN (400.18 MHz, MeCN, 298 K).

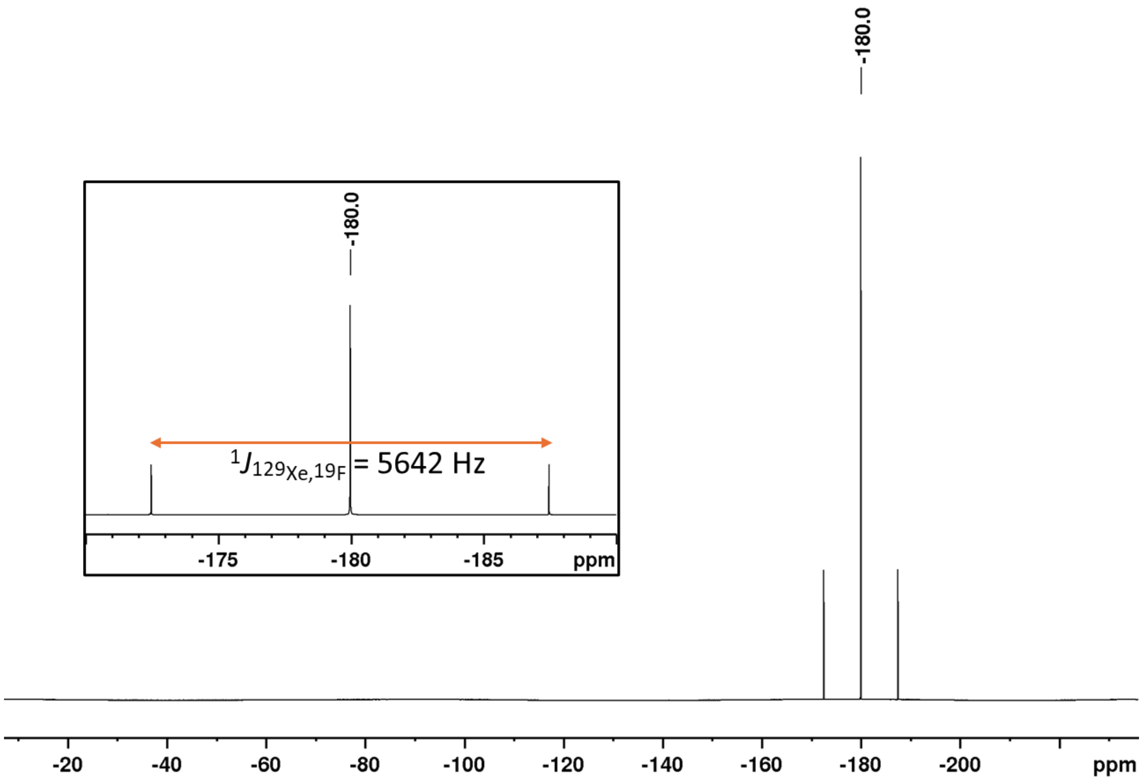

Figure S 13: ^{19}F NMR spectrum of XeF_2 in MeCN (376.54 MHz, MeCN, 298 K).

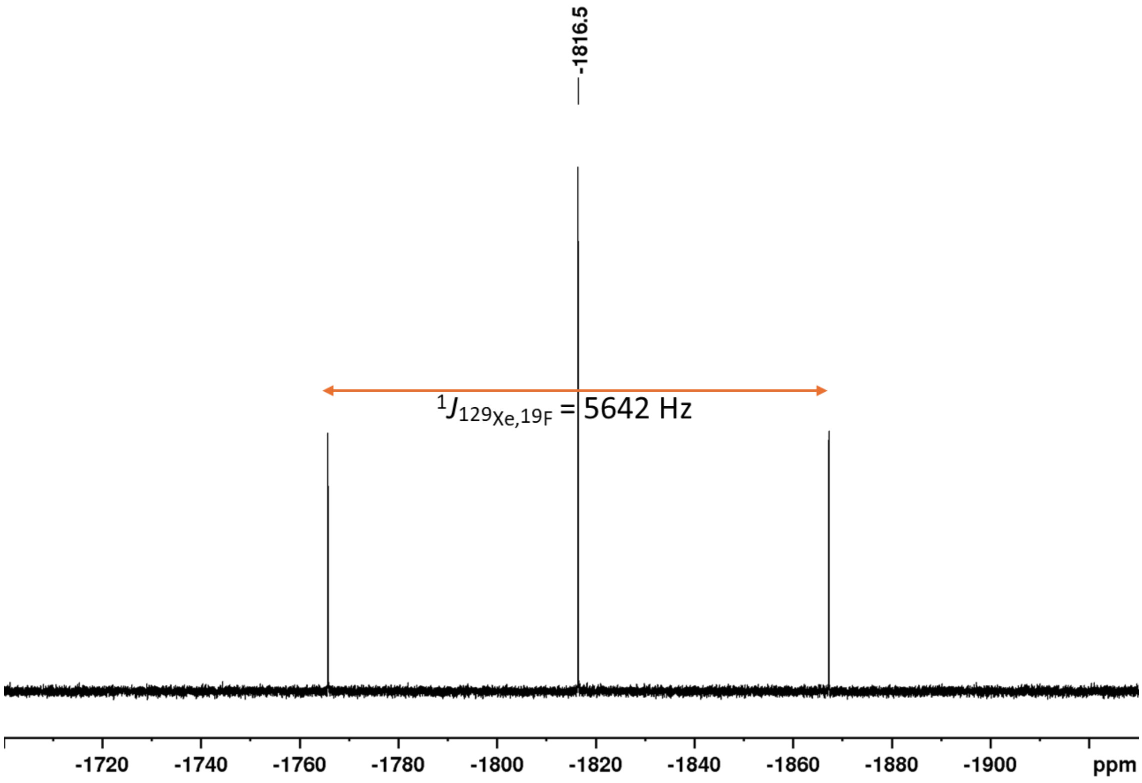

Figure S 14: ^{129}Xe NMR spectrum of XeF_2 in MeCN (111.29 MHz, MeCN, 298 K, calibrated to $XeOF_4$).

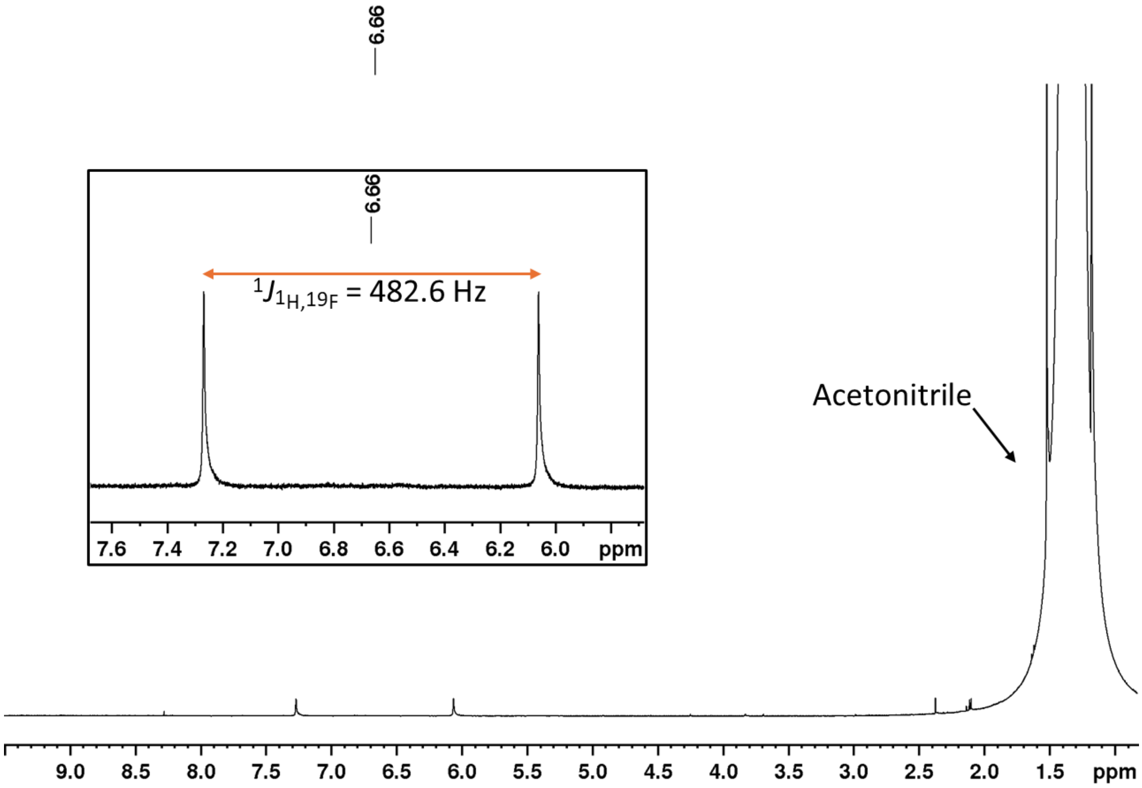

Figure S 15: ^{1}H NMR spectrum of XeF_2 in MeCN after 3d (400.18 MHz, MeCN, 298 K).

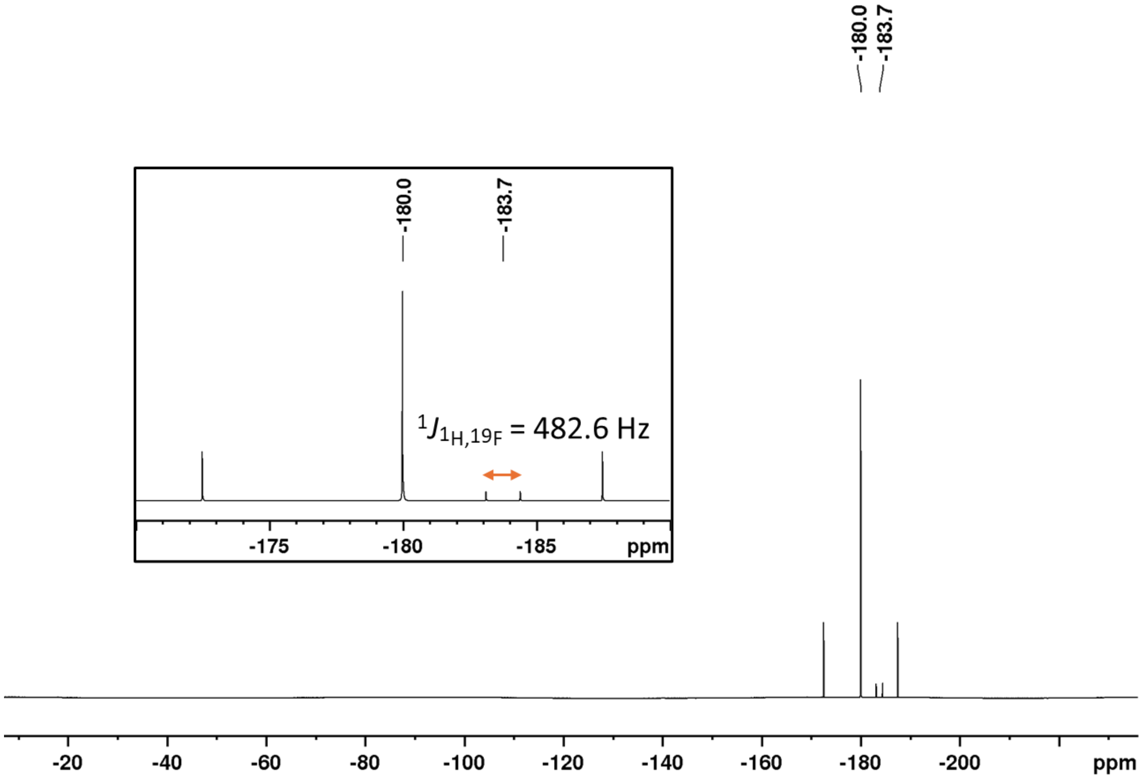

Figure S 16: ^{19}F NMR spectrum of XeF_2 in MeCN after 3d (376.54 MHz, MeCN, 298 K).

5.3. Biphenyl – Reaction of XeF_2/2Li[Al(OR^F)$_4$] with Benzene

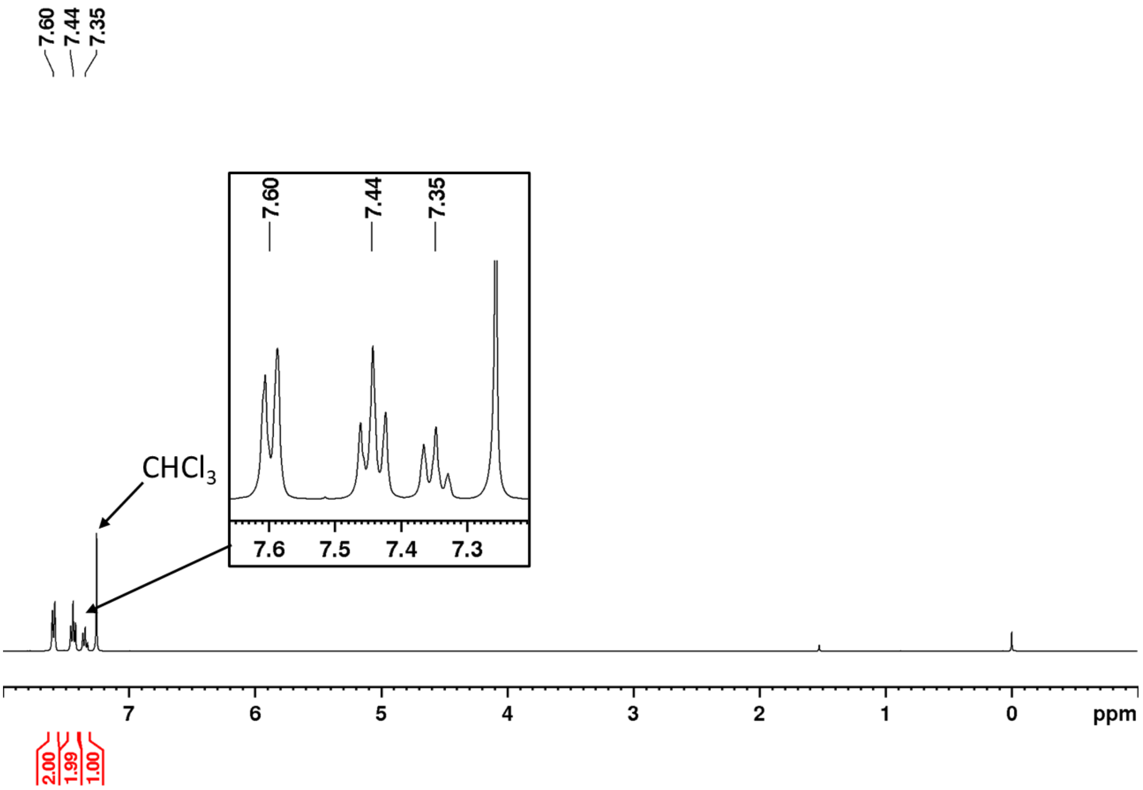

Figure S 17: ^{1}H NMR spectrum of biphenyl (400.17 MHz, $CDCl_3$, 298 K).

5.4. 4,4'-Difluorobiphenyl – Reaction of XeF_2/2Li[Al(OR^F)$_4$] with Fluorobenzene

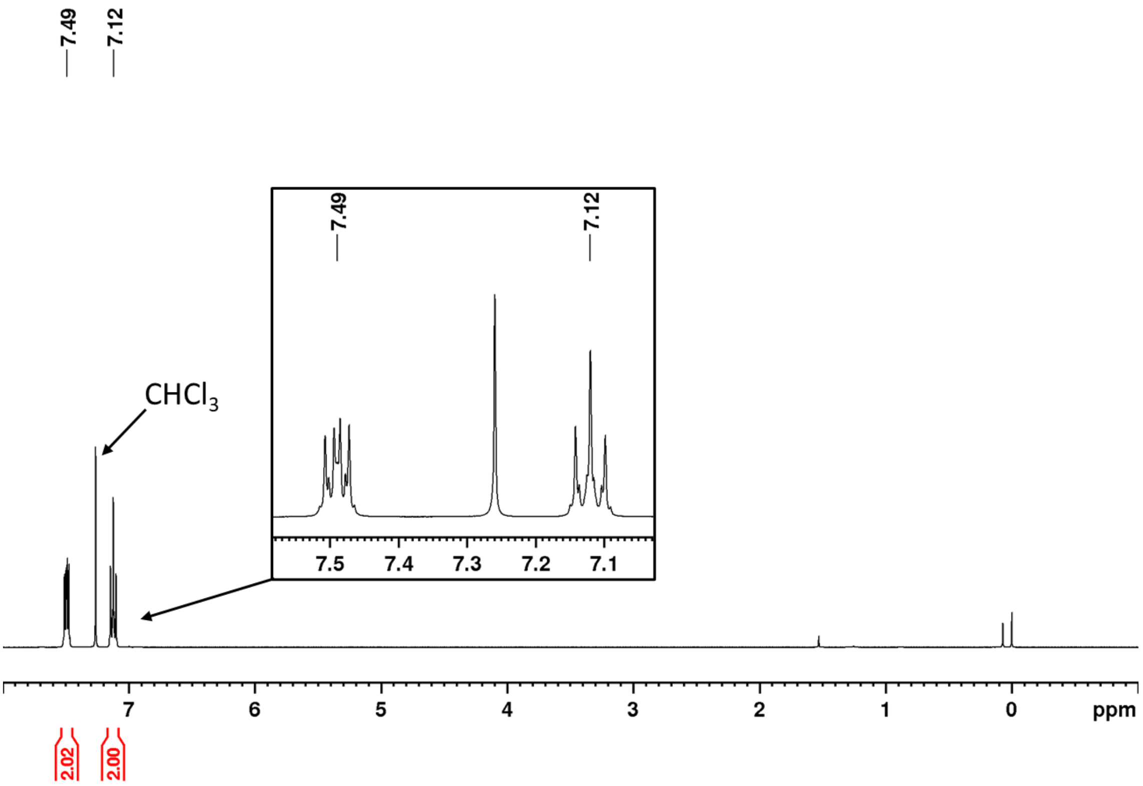

Figure S 18: ^{1}H NMR spectrum of 4,4'-difluorobiphenyl (400.17 MHz, $CDCl_3$, 298 K).

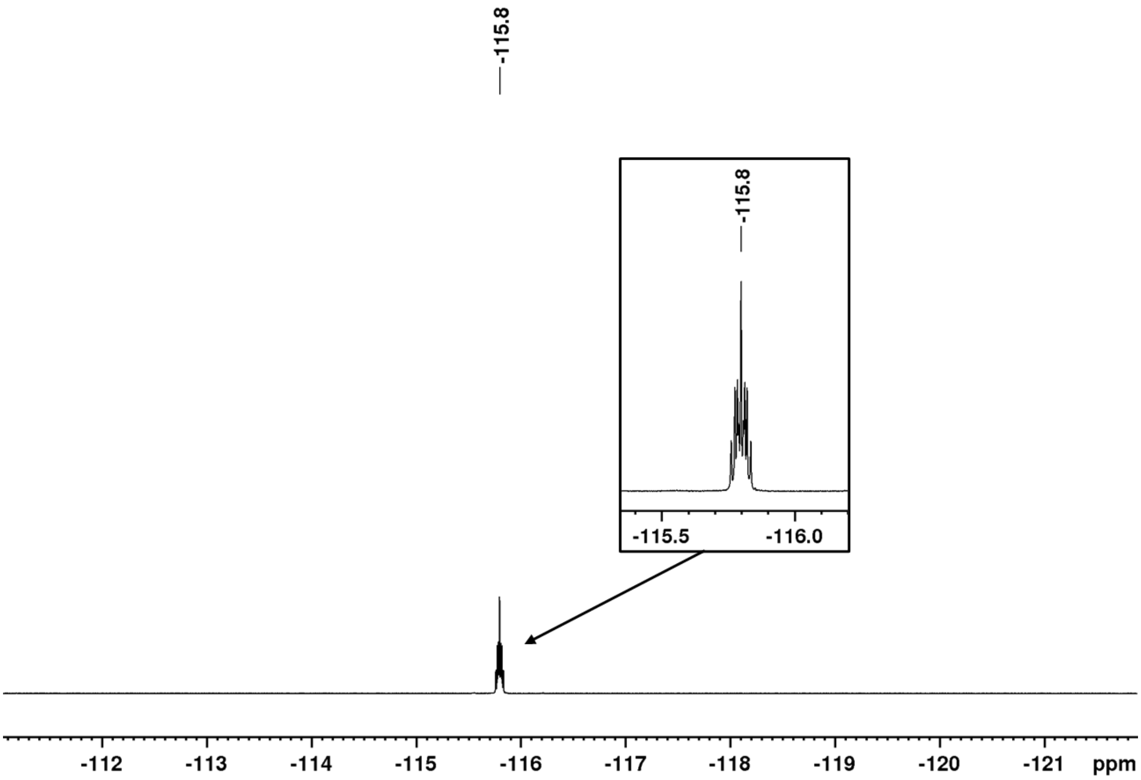

Figure S 19: ^{19}F NMR spectrum of 4,4'-difluorobiphenyl (376.53 MHz, $CDCl_3$, 298 K).

5.5. 3,3',4,4'-Tetrafluorobiphenyl – Reaction of XeF_2/2Li[Al(OR^F)$_4$] with 1,2-Difluorobenzene

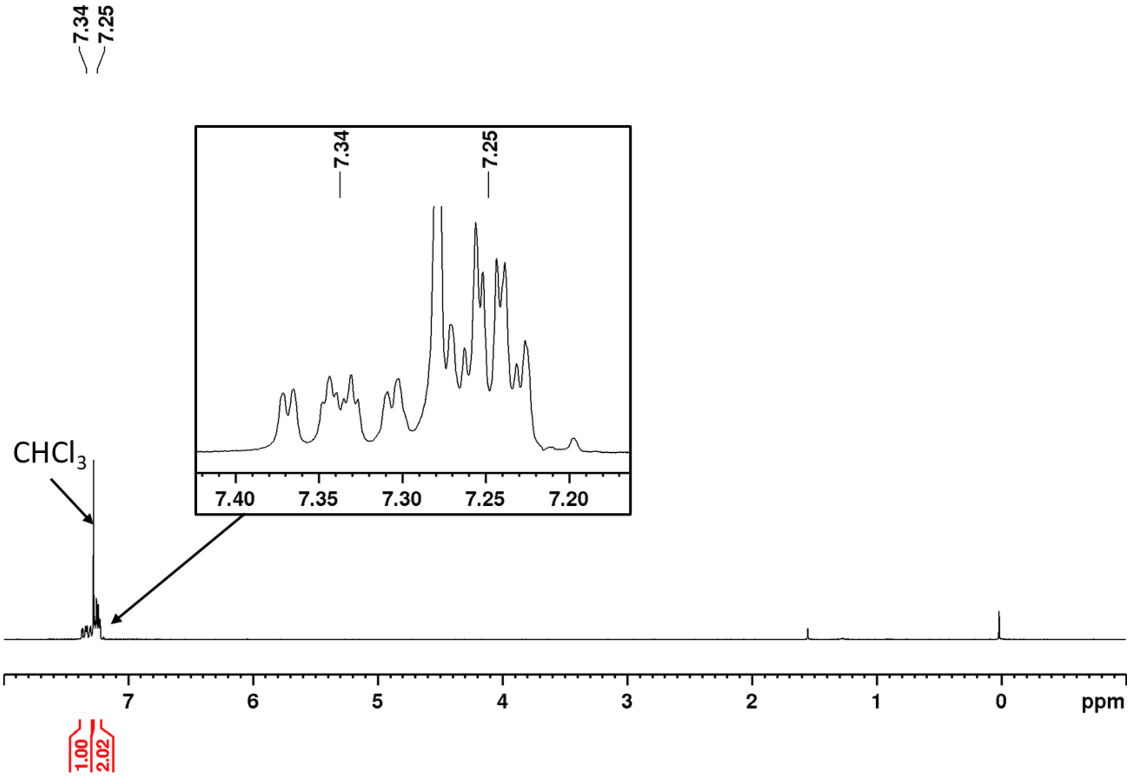

Figure S 20: ^{1}H NMR spectrum of 3,3',4,4'-tetrafluorobiphenyl (300.18 MHz, $CDCl_3$, 298 K).

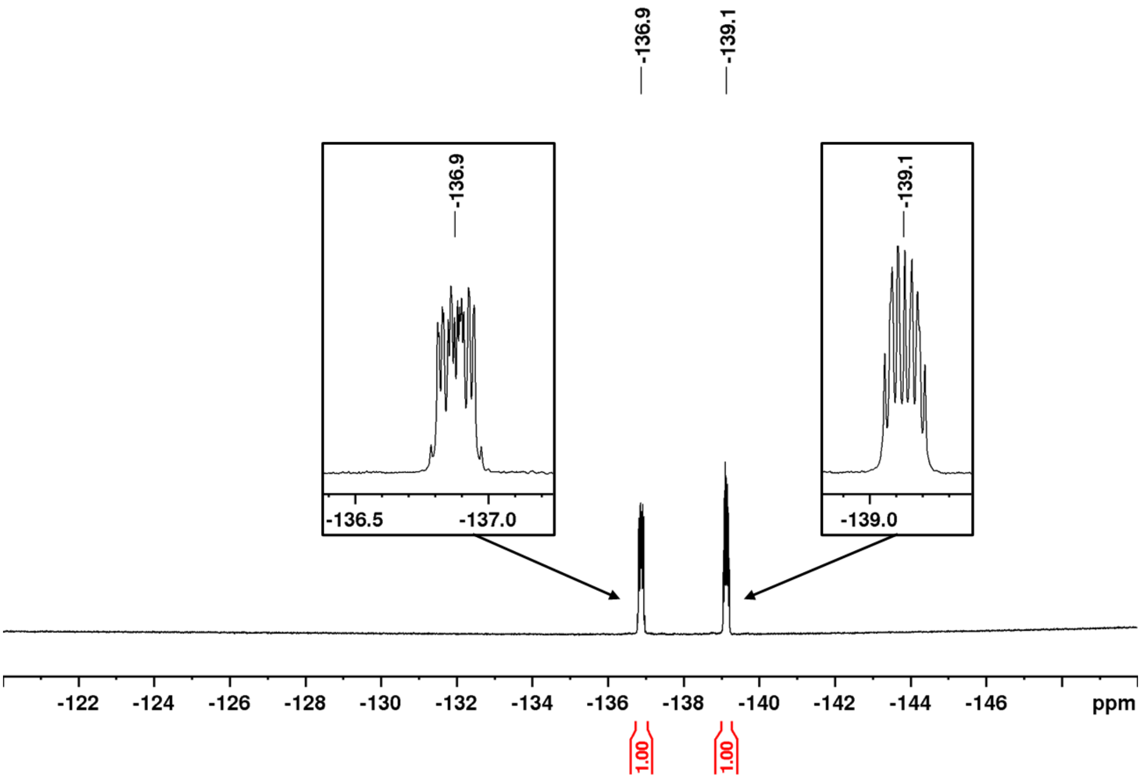

Figure S 21: ^{19}F NMR spectrum of 3,3',4,4'-tetrafluorobiphenyl (282.45 MHz, $CDCl_3$, 298 K).

5.6. $[2,3,4\text{-}C_6H_2F_3Xe]^+$ – Reaction of XeF_2/2Li[Al(OR^F)$_4$] with 1,2,3-Trifluorobenzene

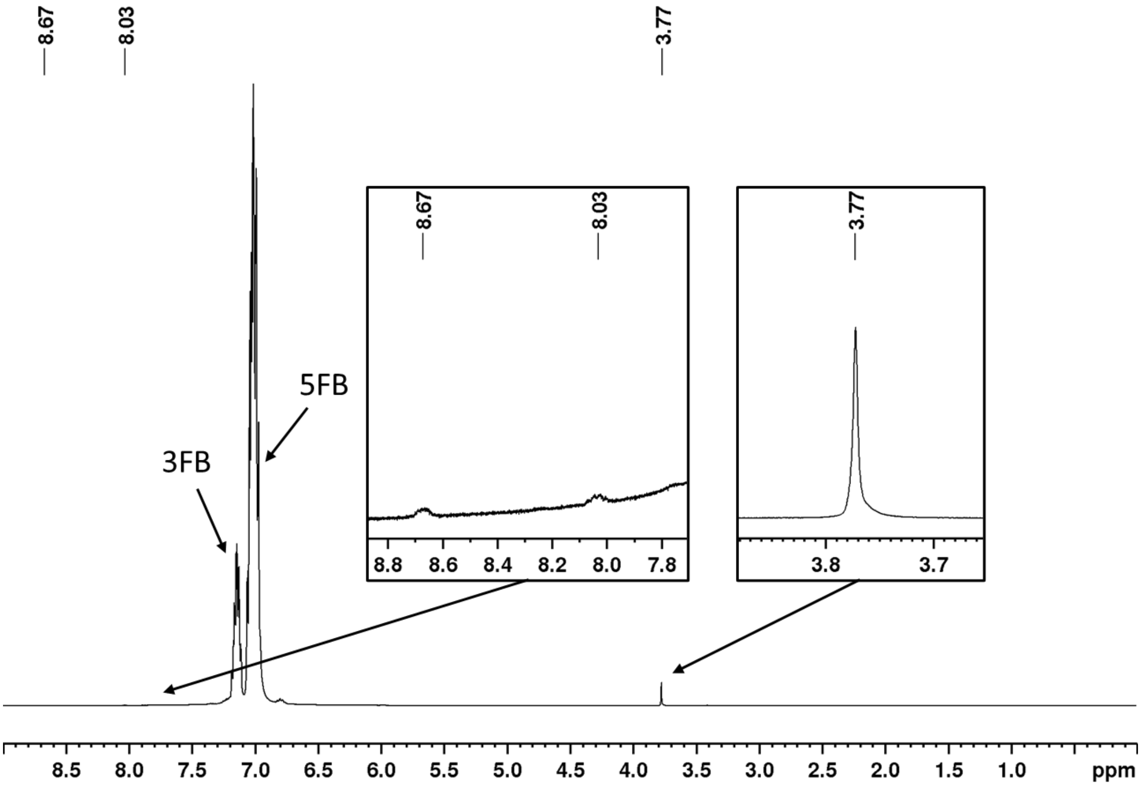

Figure S 22: ^{1}H NMR spectrum of the reaction of XeF_2/2Li[Al(OR^F)$_4$] with 3FB (400.17 MHz, 3FB/5FB (1:4), 243 K).

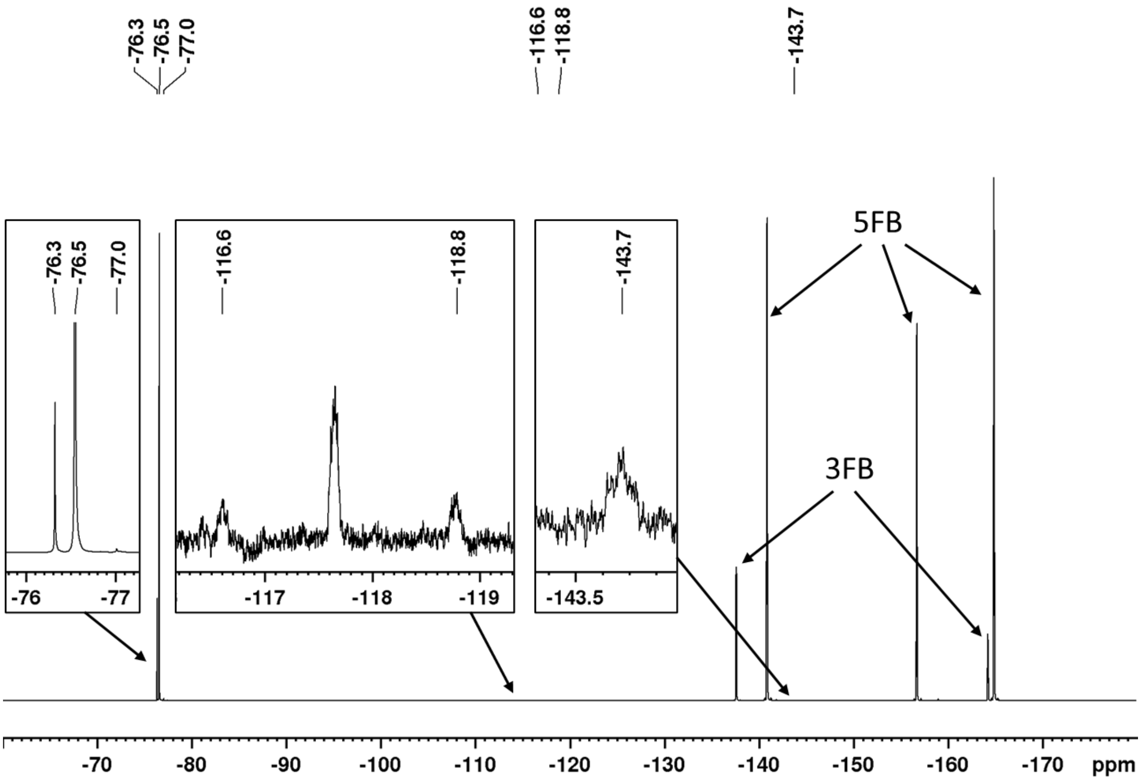

Figure S 23: ^{19}F NMR spectrum of the reaction of XeF_2/2Li[Al(OR^F)$_4$] with 3FB (376.54 MHz, 3FB/5FB (1:4), 243 K).

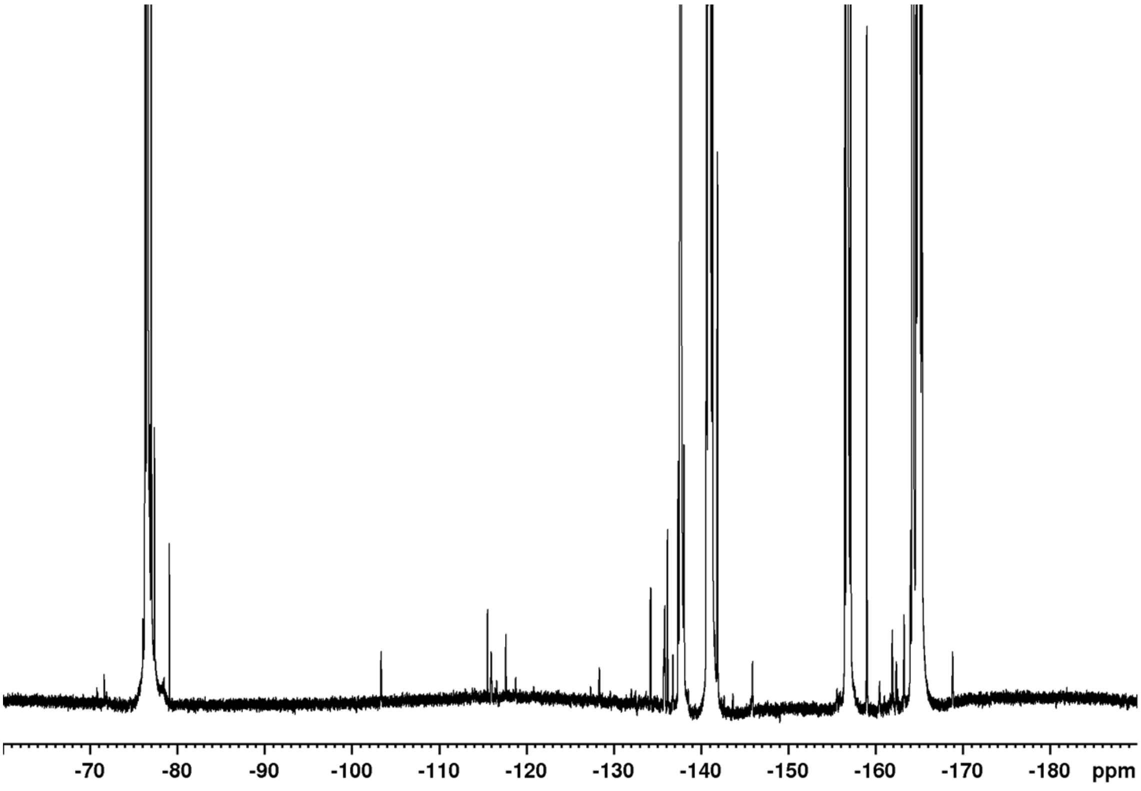

Figure S 24: ^{19}F NMR spectrum of the reaction of XeF_2/2Li[Al(OR^F)$_4$] with 3FB (376.54 MHz, 3FB/5FB (1:4), 243 K).

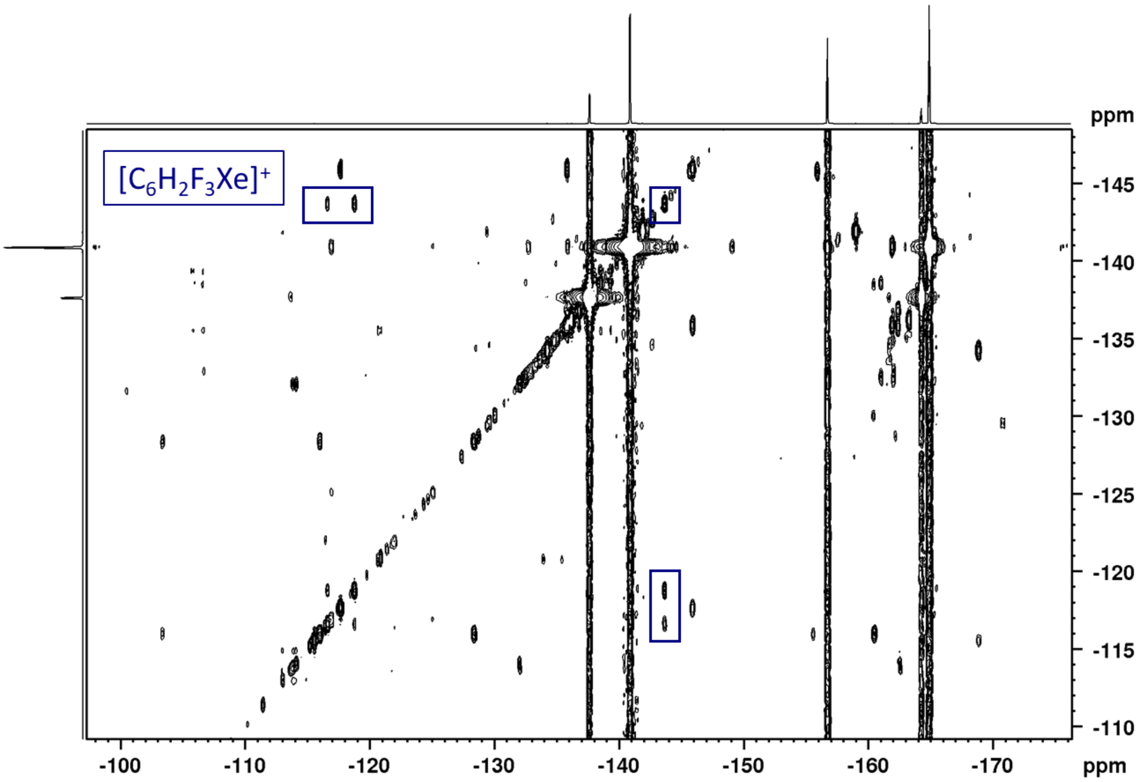

Figure S 25: ^{19}F-^{19}F COSY NMR spectrum of the reaction of XeF_2/2Li[Al(OR^F)$_4$] with 3FB (376.54 MHz, 3FB/5FB (1:4), 243 K, optimized for J = 15 Hz).

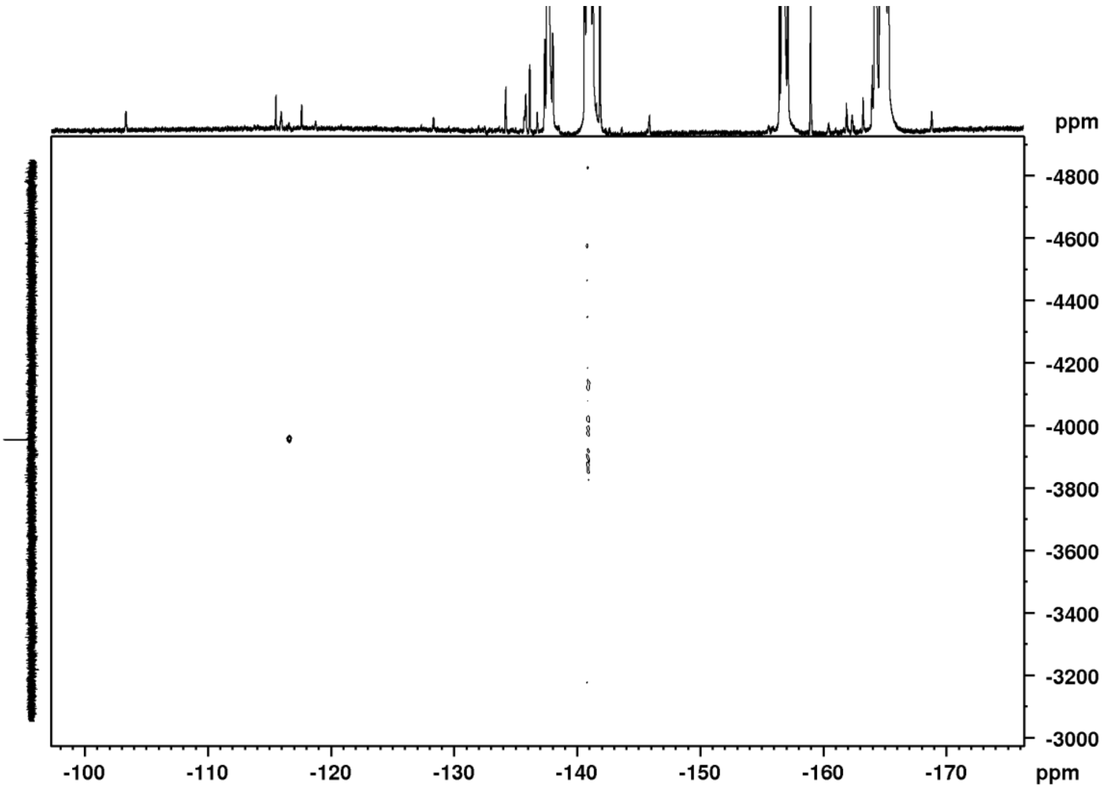

Figure S 26: ^{19}F-^{129}Xe HMBC NMR spectrum of the reaction of XeF_2/2Li[Al(OR^F)$_4$] with 3FB (376.54 MHz, 3FB/5FB (1:4), 243 K, optimized for J = 100 Hz).

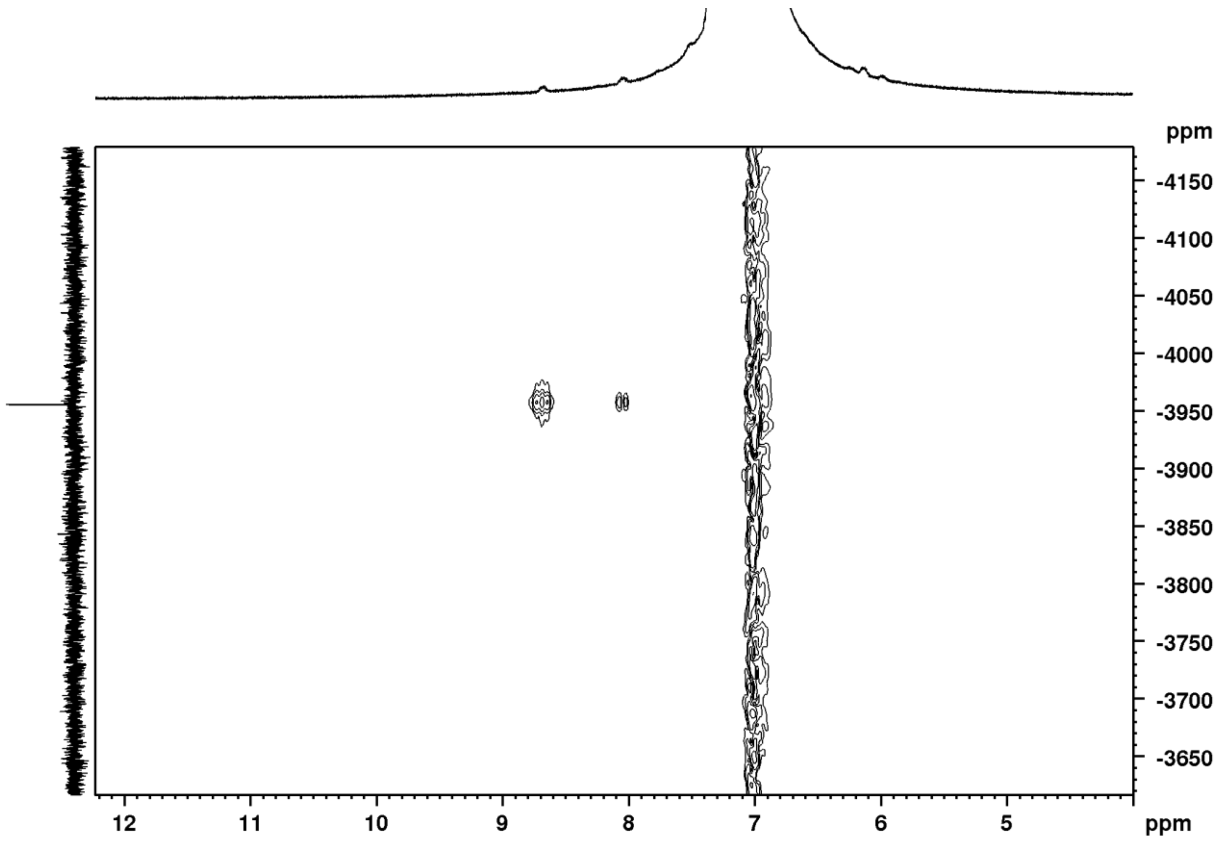

Figure S 27: ^{1}H-^{129}Xe HMBC NMR spectrum of the reaction of XeF_2/2Li[Al(OR^F)$_4$] with 3FB (400.17 MHz, 3FB/5FB (1:4), 243 K, optimized for J = 50 Hz).

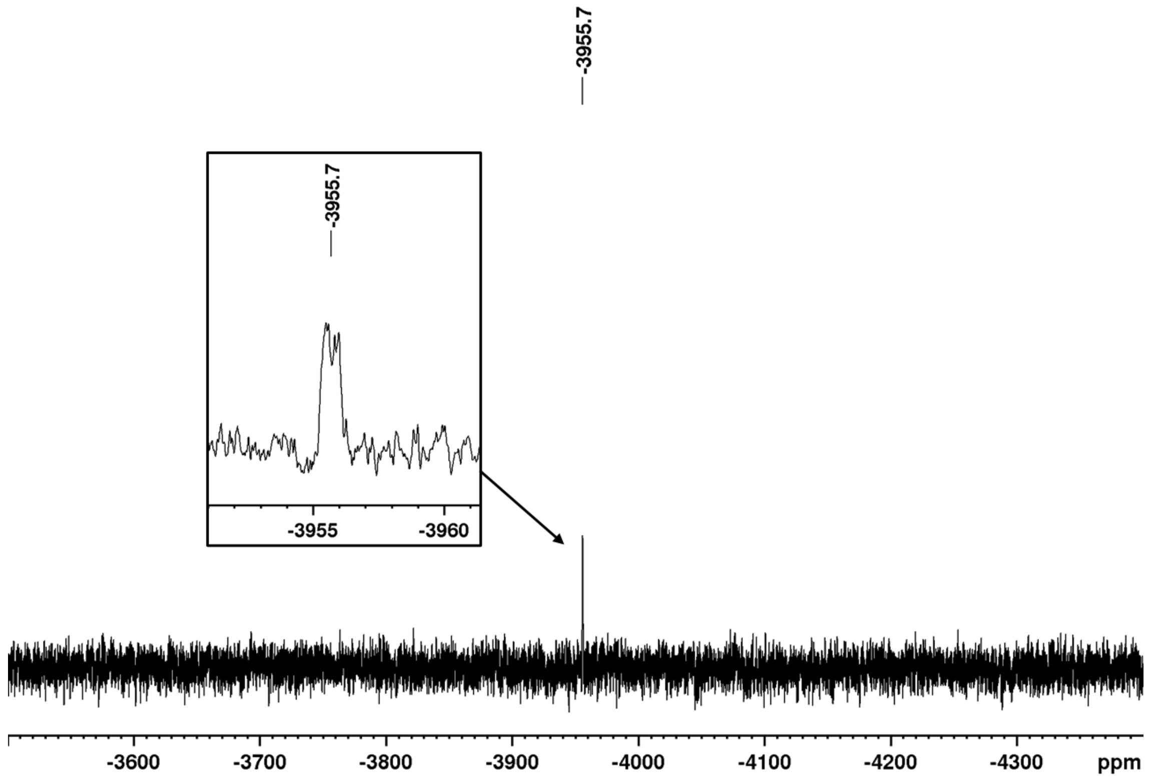

Figure S 28: ^{129}Xe NMR spectrum of the reaction of XeF_2/2Li[Al(OR^F)$_4$] with 3FB (111.29 MHz, 3FB/5FB (1:4), 243 K).

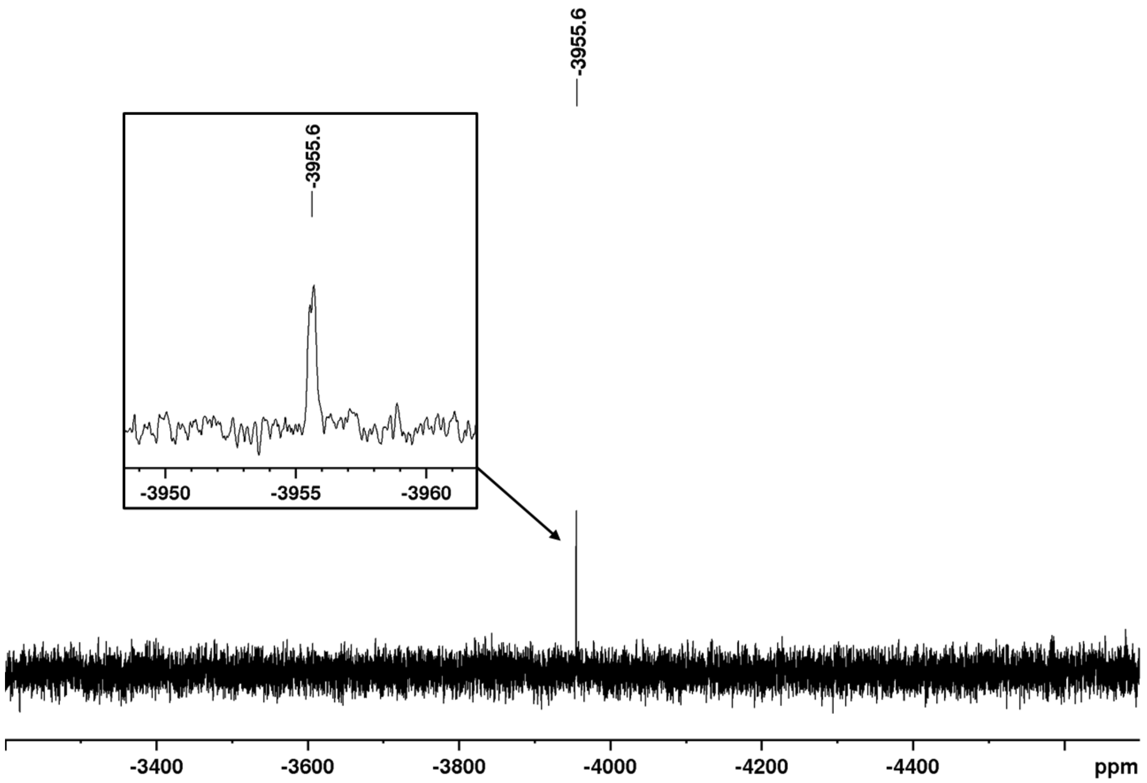

Figure S 29: $^{129}Xe\{^{19}F\}$ NMR spectrum of the reaction of XeF_2/2Li[Al(OR^F)$_4$] with 3FB (111.29 MHz, 3FB/5FB (1:4), 243 K).

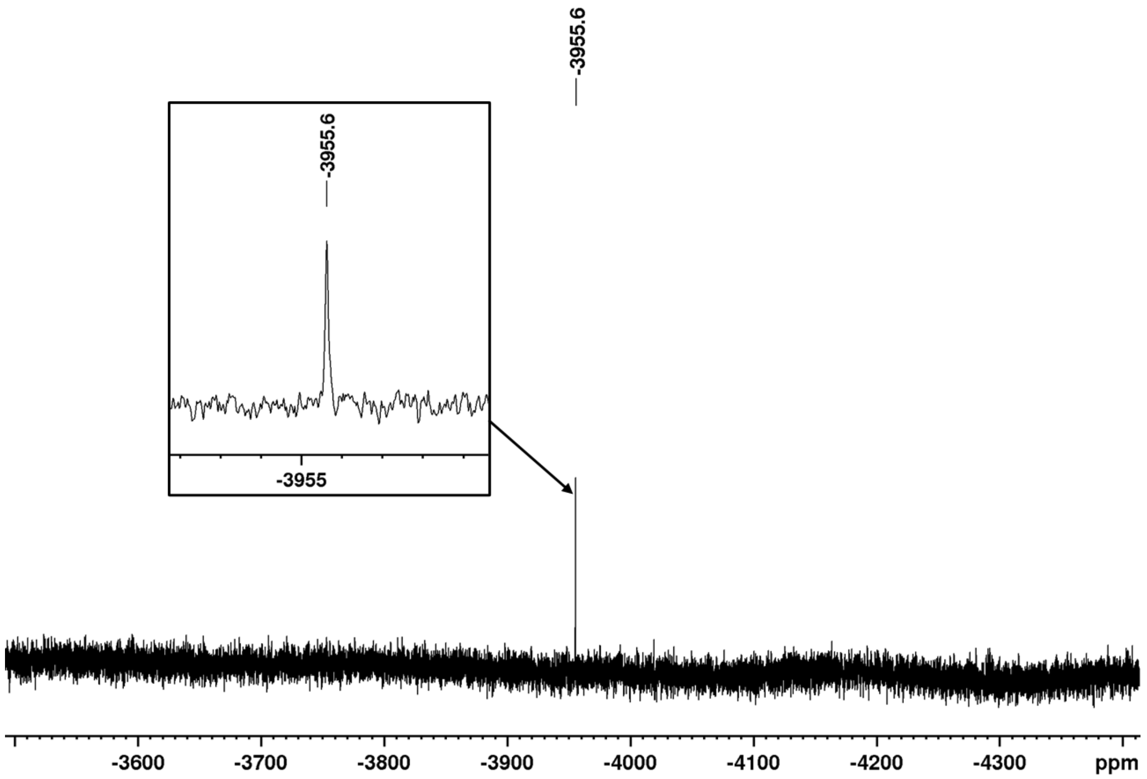

Figure S 30: $^{129}Xe\{^{1}H, ^{19}F\}$ NMR spectrum of the reaction of XeF_2/2Li[Al(OR^F)$_4$] with 3FB (111.29 MHz, 3FB/5FB (1:4), 243 K).

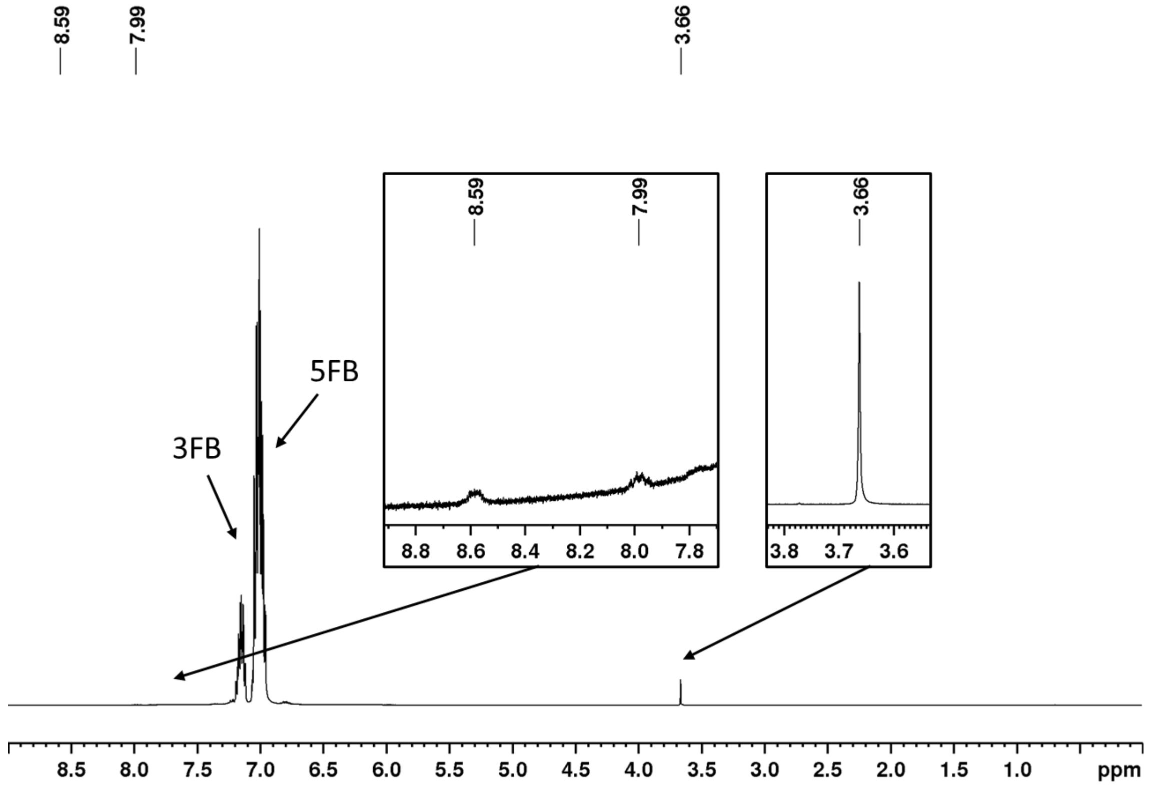

Figure S 31: ^{1}H NMR spectrum of the reaction of XeF_2/2Li[Al(OR^F)$_4$] with 3FB (400.17 MHz, 3FB/5FB (1:4), 298 K).

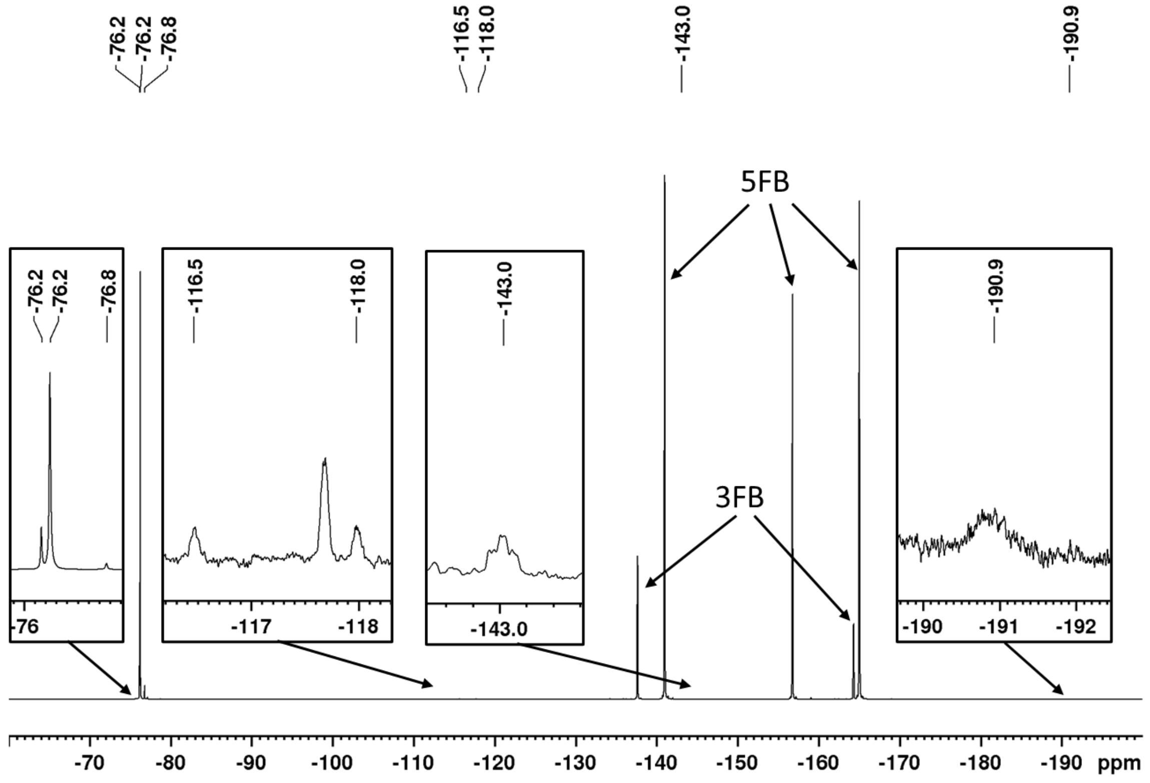

Figure S 32: ^{19}F NMR spectrum of the reaction of XeF_2/2Li[Al(OR^F)$_4$] with 3FB (376.54 MHz, 3FB/5FB (1:4), 298 K).

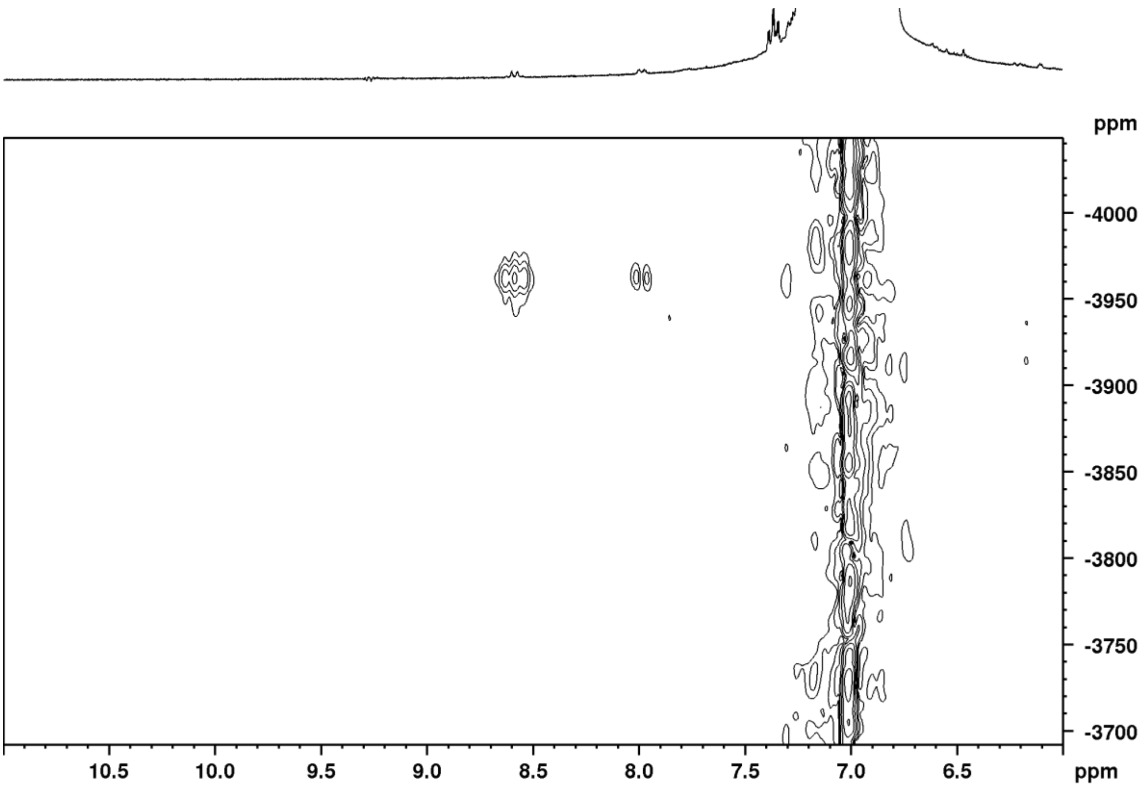

Figure S 33: ^{1}H-^{129}Xe HMBC NMR spectrum of the reaction of XeF_2/2Li[Al(OR^F)$_4$] with 3FB (400.17 MHz, 3FB/5FB (1:4), 298 K, optimized for J = 50 Hz).

5.7. [C_6F_5Xe][FAl(OR^F)$_3$]

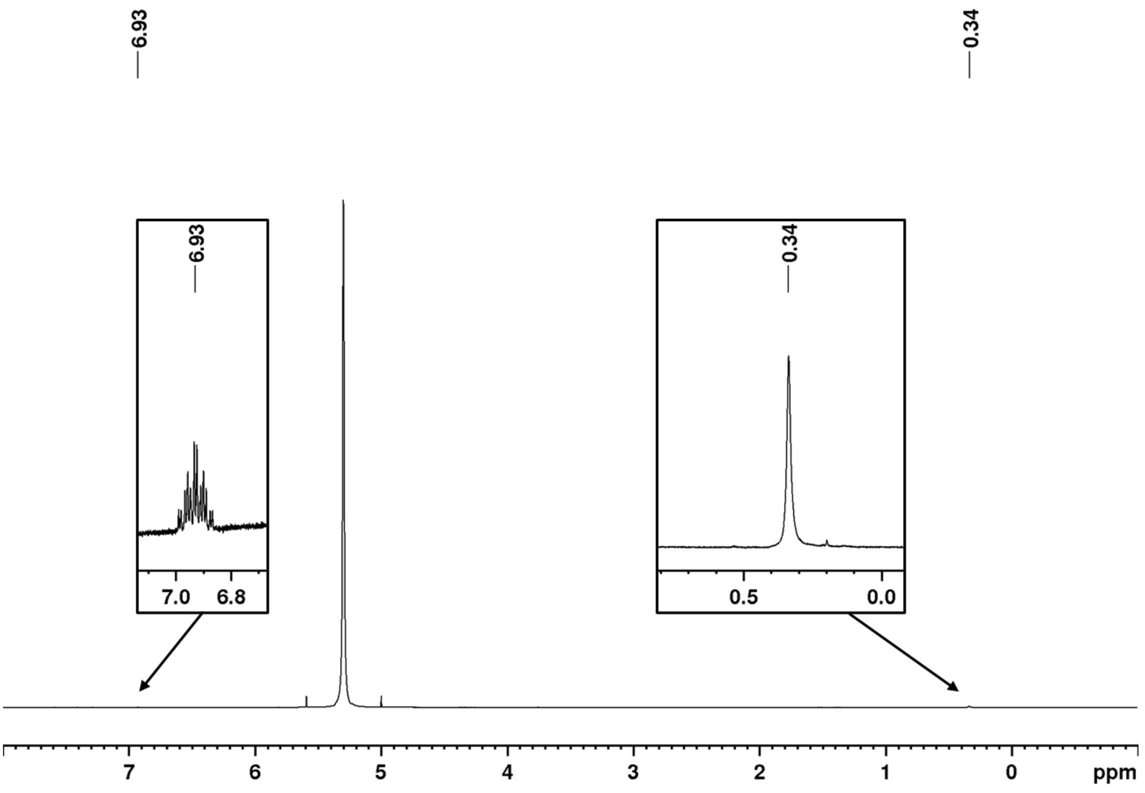

Figure S 34: ^{1}H NMR spectrum of the isolated crystals from the reaction XeF_2 + B(C_6F_5)$_3$ + Me_3SiF-Al(OR^F)$_3$ (300.18 MHz, CH_2Cl_2, 298 K).

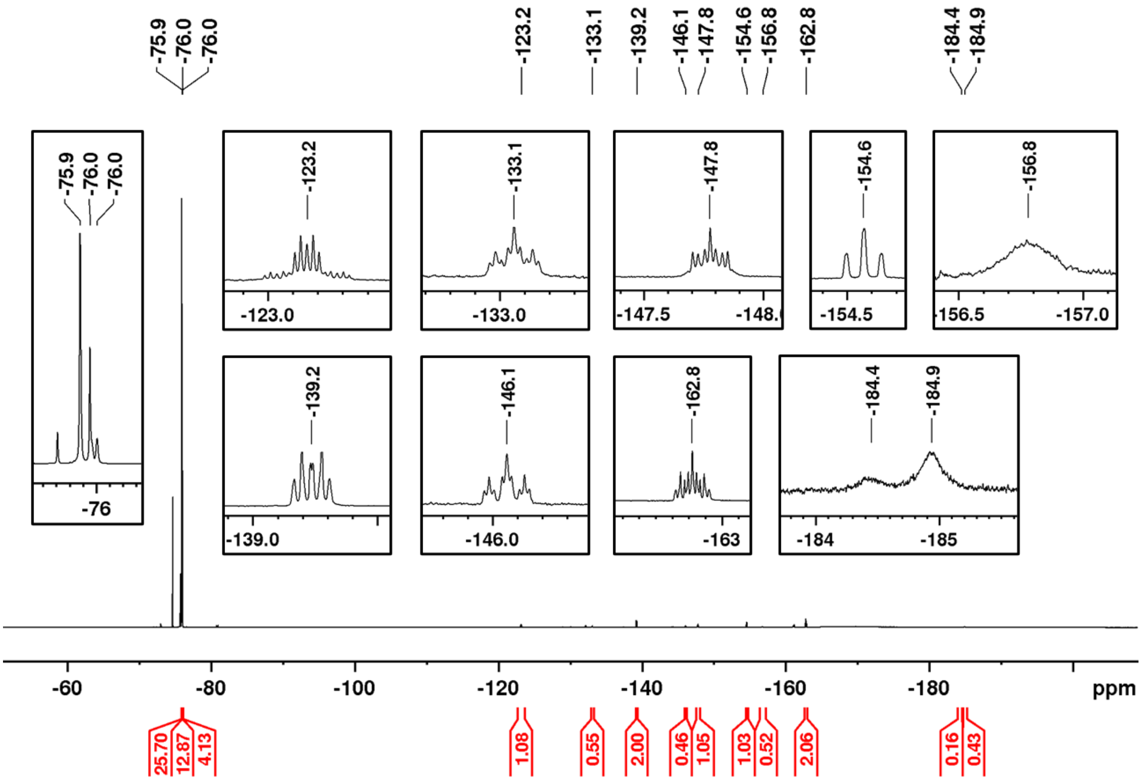

Figure S 35: ^{19}F NMR spectrum of the isolated crystals from the reaction $XeF_2 + B(C_6F_5)_3 + Me_3SiF\text{-}Al(OR^F)_3$ (282.45 MHz, CH_2Cl_2, 298 K).

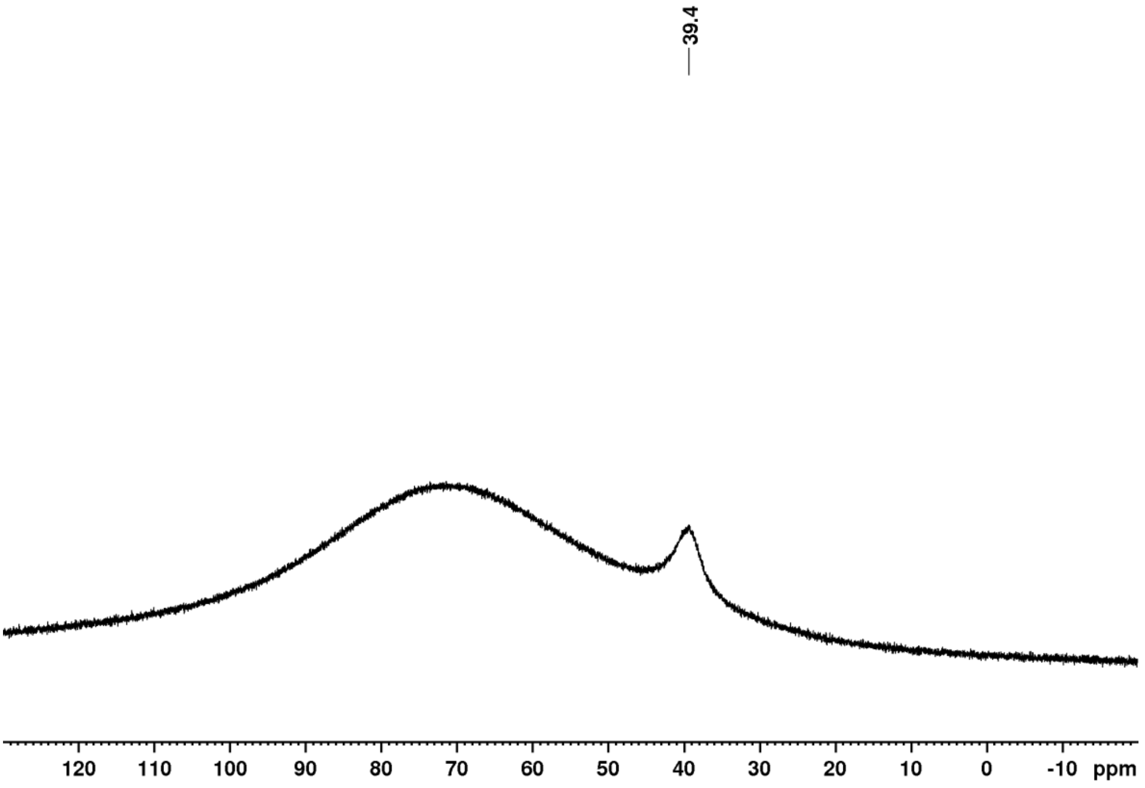

Figure S 36: ^{27}Al NMR spectrum of the isolated crystals from the reaction $XeF_2 + B(C_6F_5)_3 + Me_3SiF\text{-}Al(OR^F)_3$ (78.22 MHz, CH_2Cl_2, 298 K).

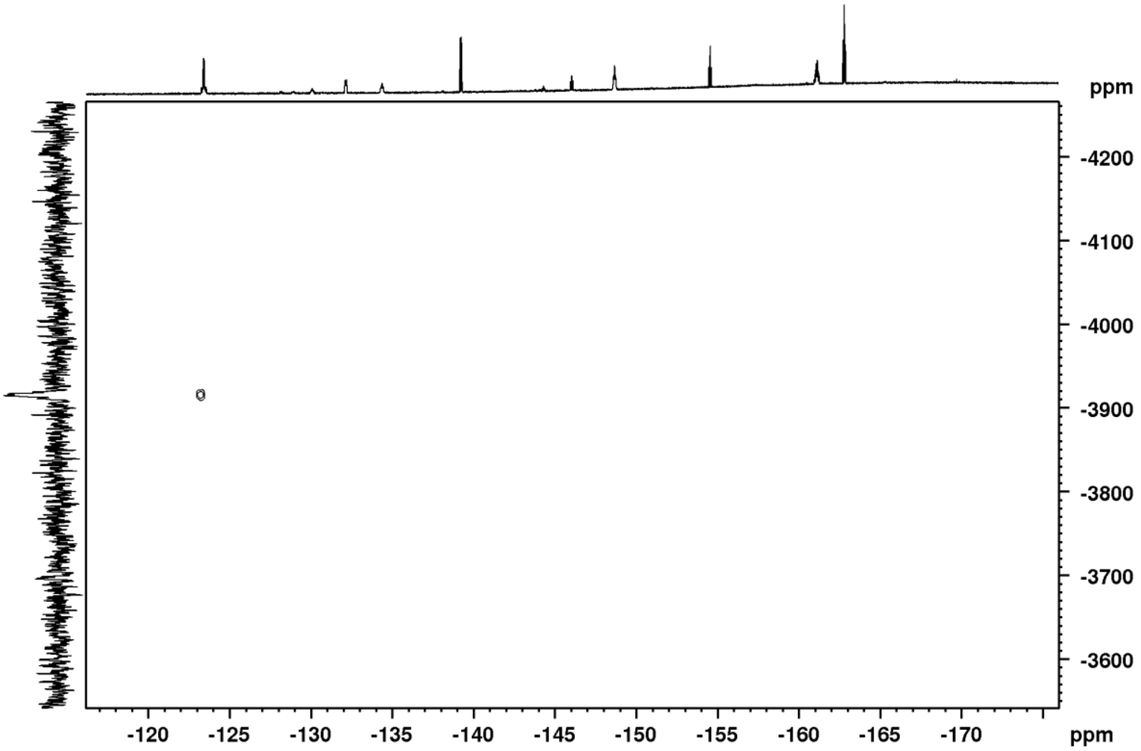

Figure S 37: ^{19}F-^{129}Xe HMBC NMR spectrum of the isolated crystals from the reaction XeF_2 + $B(C_6F_5)_3$ + Me_3SiF-$Al(OR^F)_3$ (282.45 MHz, CH_2Cl_2, 298 K, optimized for J = 70 Hz).

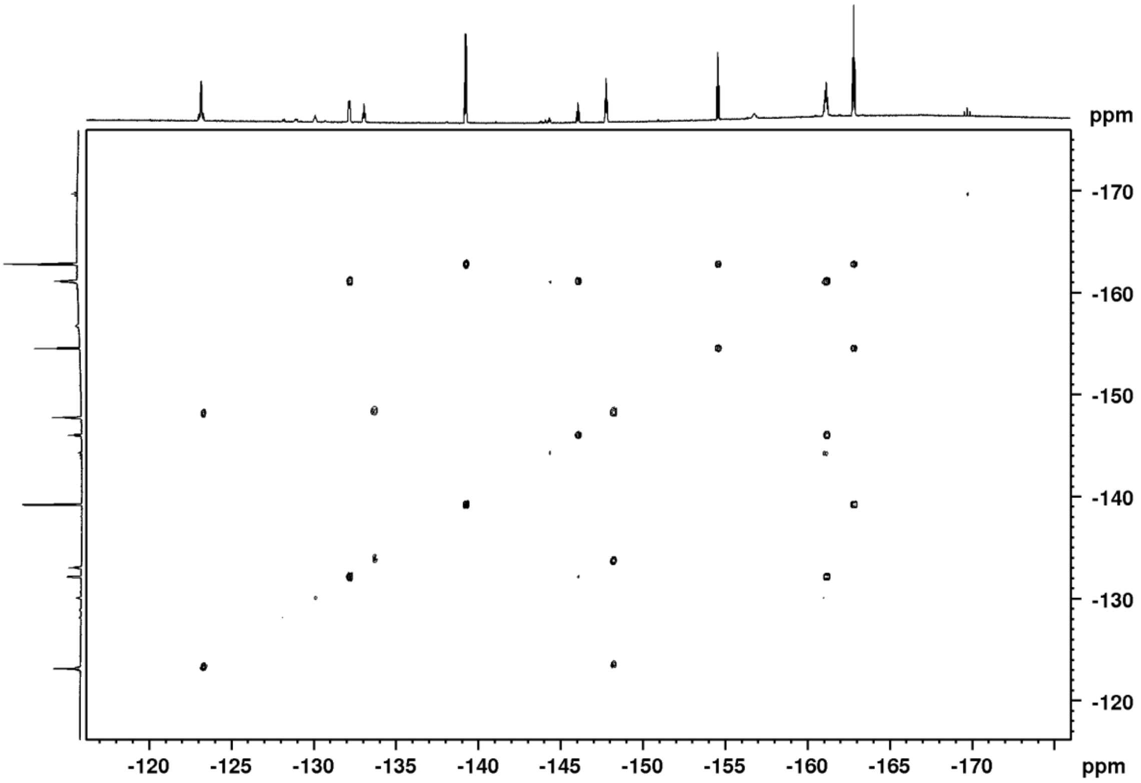

Figure S 38: ^{19}F-^{19}F COSY NMR spectrum of the isolated crystals from the reaction XeF_2 + $B(C_6F_5)_3$ + Me_3SiF-$Al(OR^F)_3$ (282.45 MHz, CH_2Cl_2, 298 K, optimized for J = 15 Hz).

5.8. $[C_6F_5Xe][F(Al(OR^F)_3)_2]$

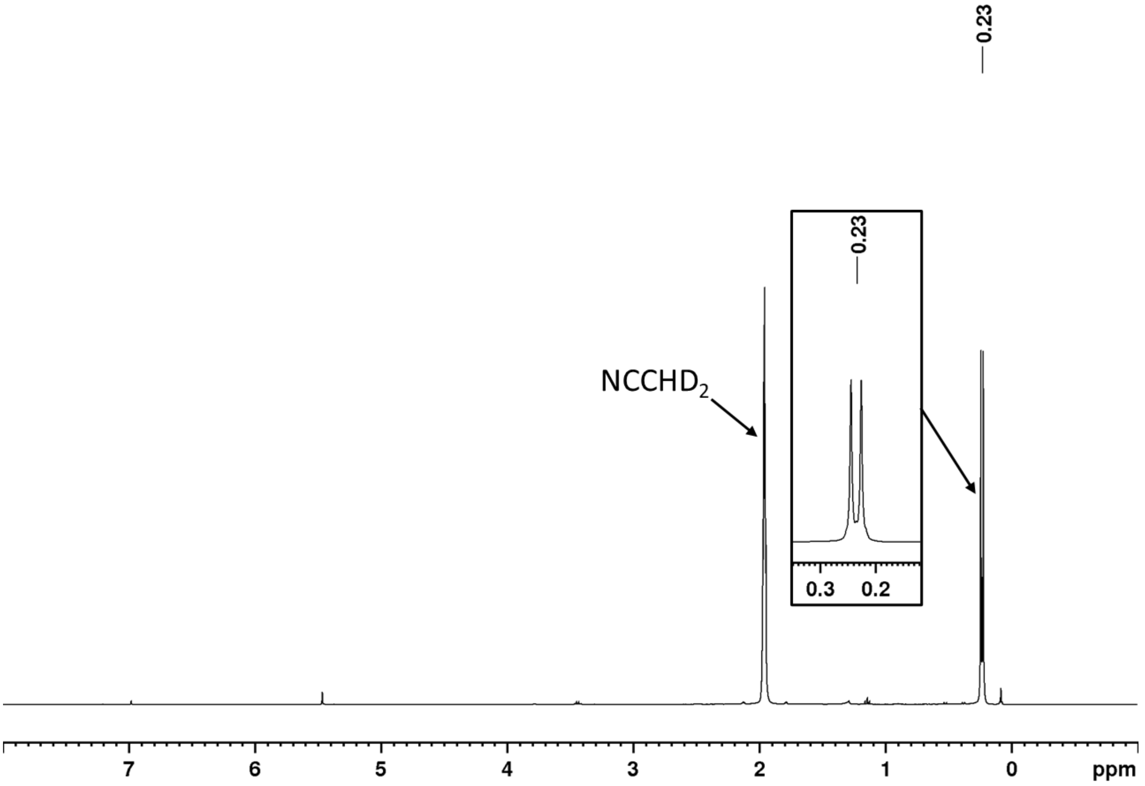

Figure S 39: ^1H NMR spectrum of the isolated crystals from the reaction XeF_2 + $B(C_6F_5)_3$ + 2 Me_3SiF-$Al(OR^F)_3$ (400.17 MHz, $NCCD_3$, 298 K).

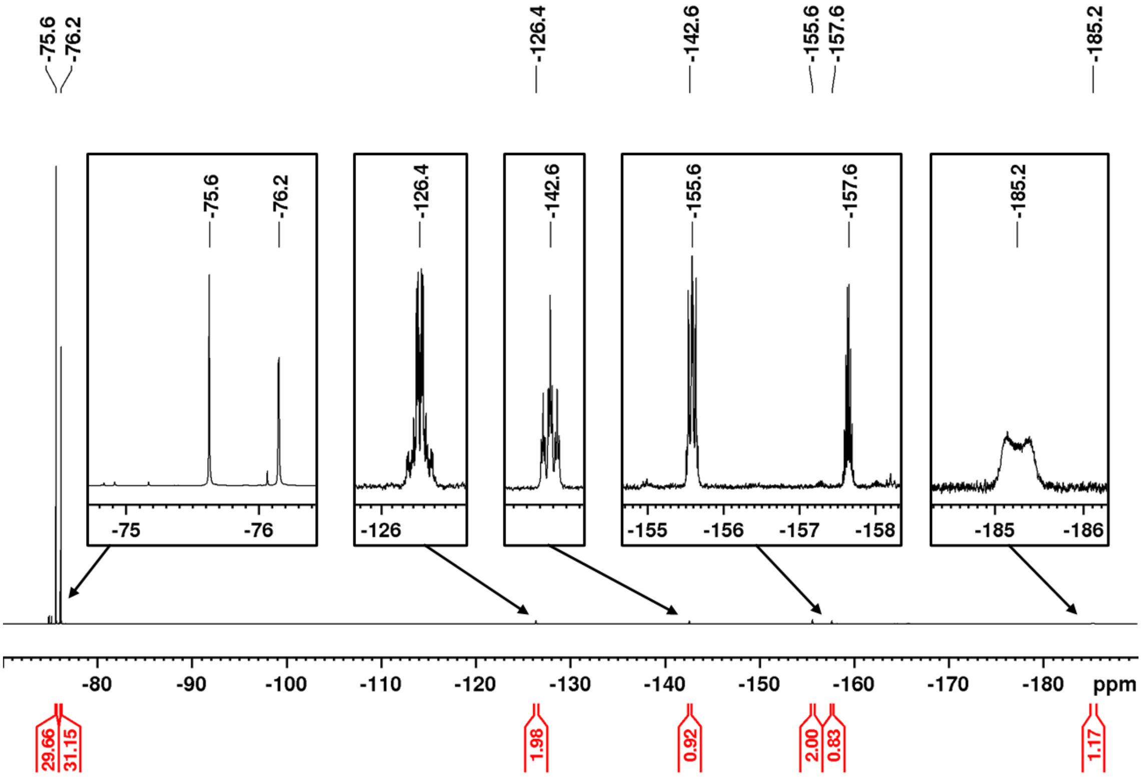

Figure S 40: ^{19}F NMR spectrum of the isolated crystals from the reaction XeF_2 + $B(C_6F_5)_3$ + 2 Me_3SiF-$Al(OR^F)_3$ (376.54 MHz, $NCCD_3$, 298 K).

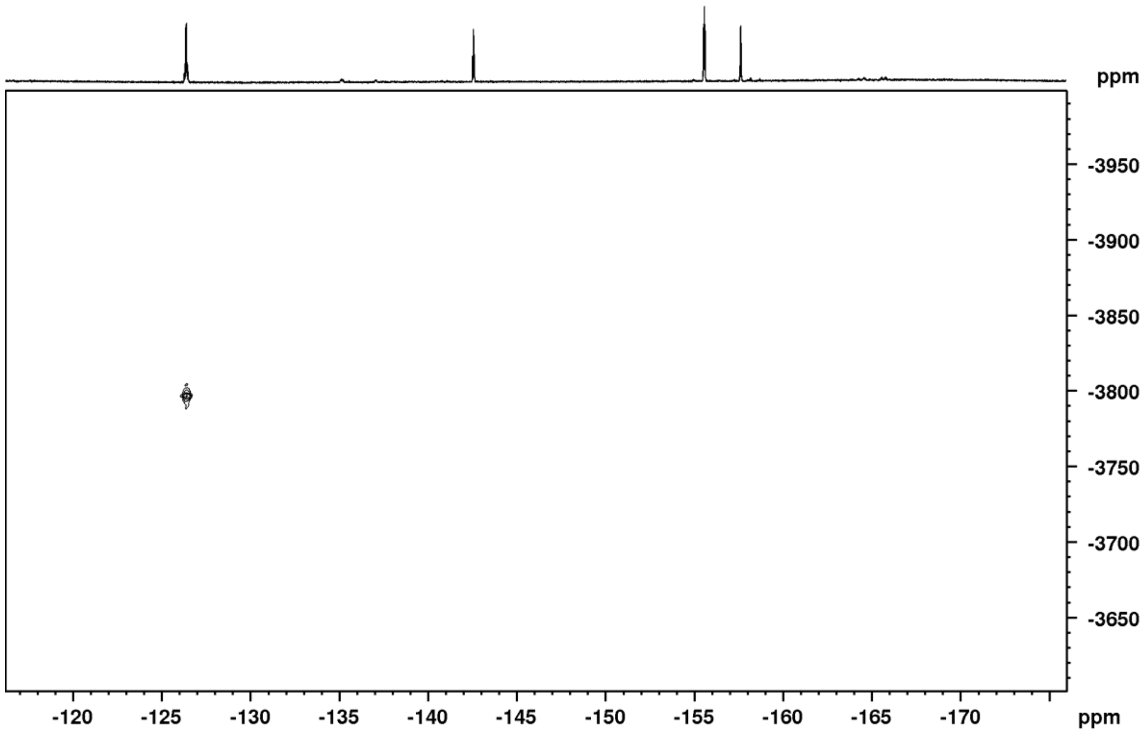

Figure S 41: ^{19}F-^{129}Xe NMR spectrum of the isolated crystals from the reaction XeF_2 + $B(C_6F_5)_3$ + 2 Me_3SiF-$Al(OR^F)_3$ (376.54 MHz, $NCCD_3$, 298 K, optimized for J = 80 Hz).

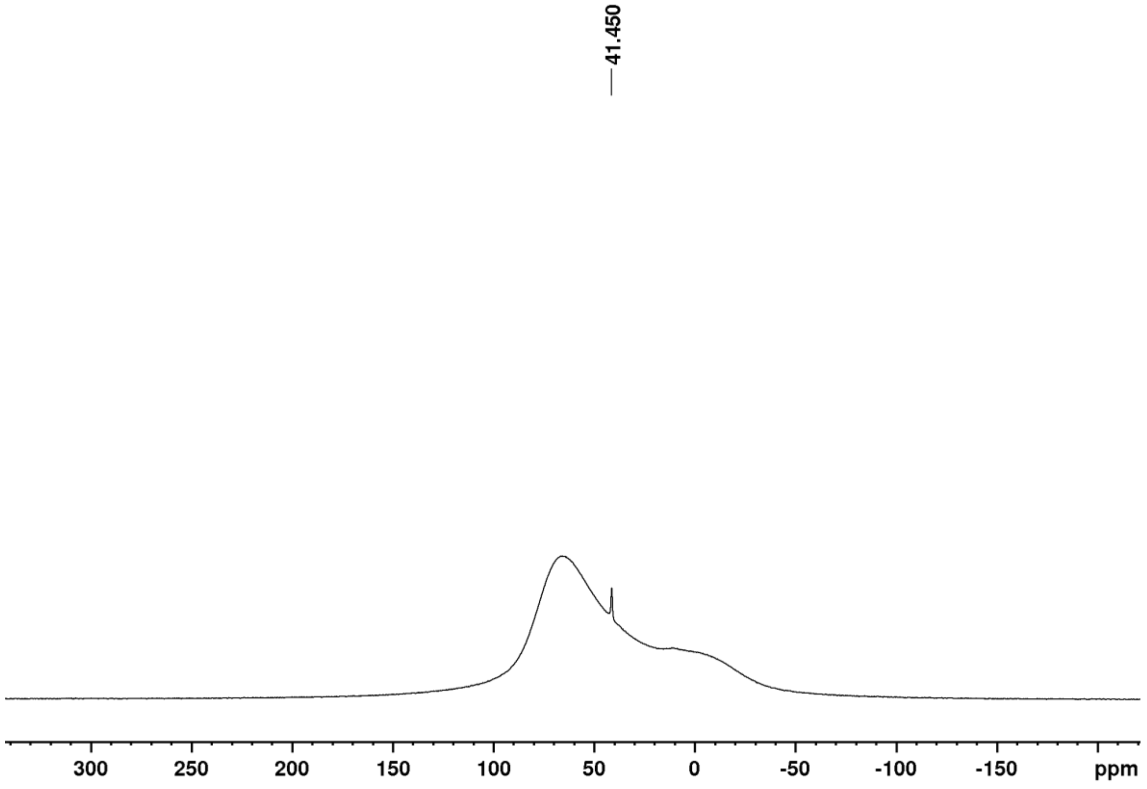

Figure S 42: ^{27}Al NMR spectrum of the isolated crystals from the reaction XeF_2 + $B(C_6F_5)_3$ + 2 Me_3SiF-$Al(OR^F)_3$ (104.27 MHz, $NCCD_3$, 298 K).

6. EPR Spectroscopy

6.1. Biphenyl – Reaction of XeF_2/2Li[Al(OR^F)$_4$] with Benzene

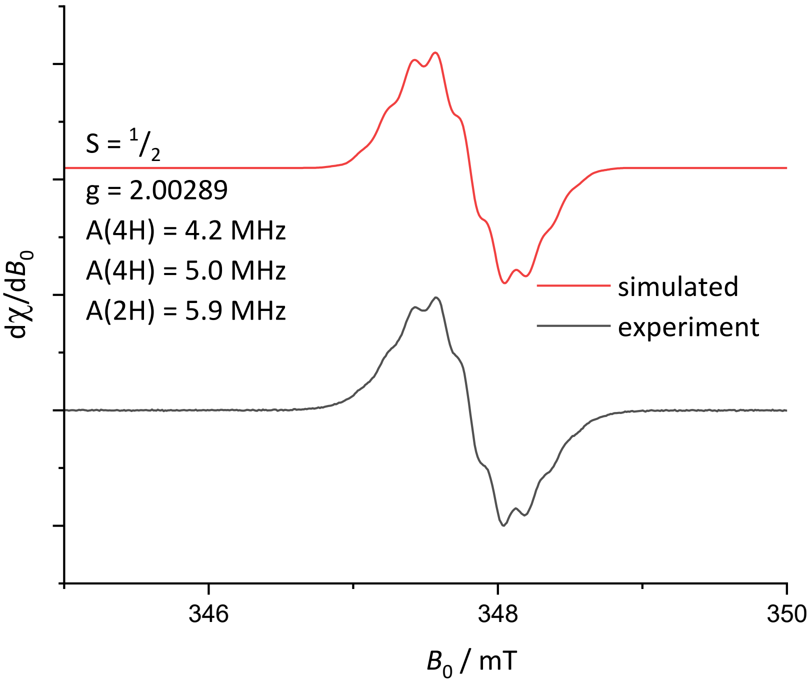

Figure S 43: Comparison of the measured EPR Spectrum of the reaction XeF_2/2Li[Al(OR^F)$_4$] in 2FB/benzene (black) with the calculated EPR spectrum of biphenyl (red).

6.2. 4,4'-Difluorobiphenyl – Reaction of XeF_2/2Li[Al(OR^F)$_4$] with Fluorobenzene

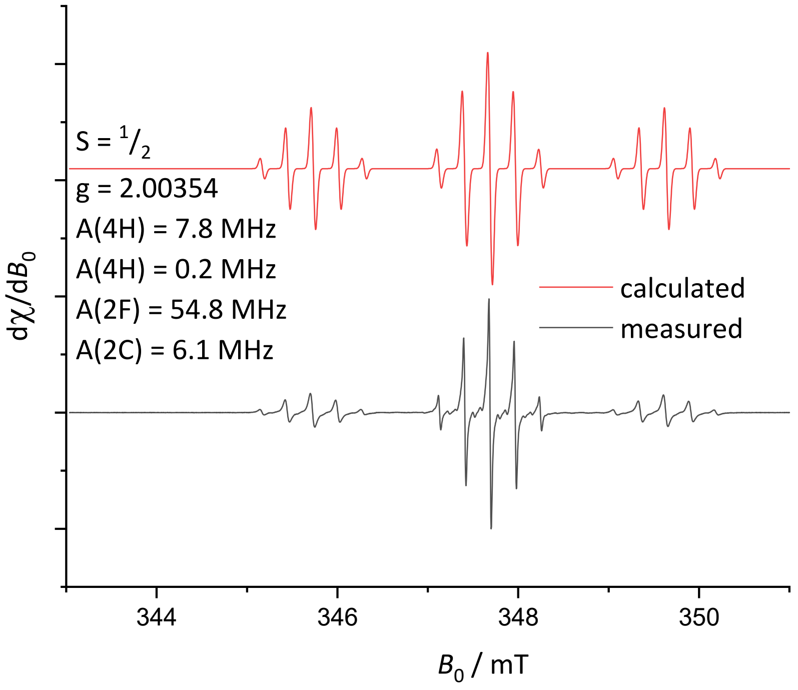

Figure S 44: Comparison of the measured EPR Spectrum of the reaction XeF_2/2Li[Al(OR^F)$_4$] in FB (black) with the calculated EPR spectrum of 4,4'-difluorobiphenyl (red).

6.3. 3,3',4,4'-Tetrafluorobiphenyl – Reaction of XeF_2/2Li[Al(OR^F)$_4$] with 1,2-Difluorobenzene

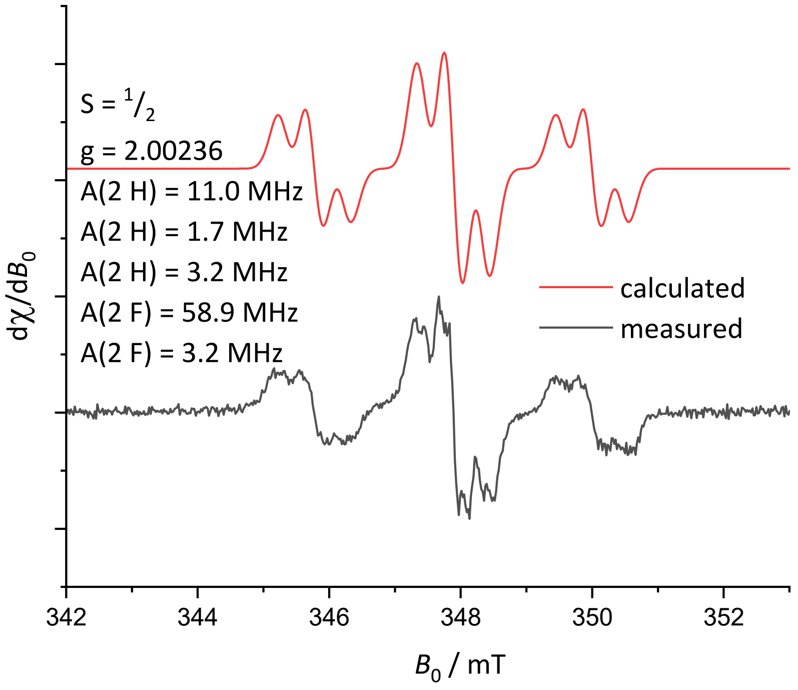

Figure S 45: Comparison of the measured EPR Spectrum of the reaction XeF_2/2Li[Al(OR^F)$_4$] in 2FB (black) with the calculated EPR spectrum of 3,3',4,4'-tetrafluorobiphenyl (red).

7. Crystal Structures

7.1. [4,4'-difluorobiphenyl][Al(OR^F)$_4$]

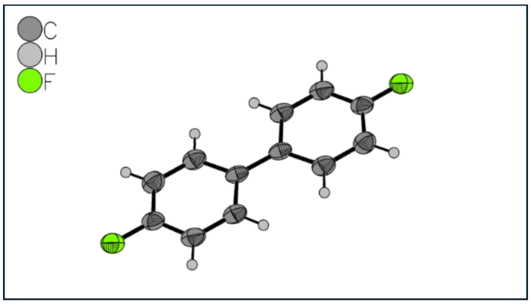

Figure S 46: Molecular structure of [fbfb][Al(OR^F)$_4$]. Thermal displacement ellipsoids are shown at 50% probability. Counter ions are omitted for clarity. The measurement was carried out at 100 K.

Table S 1: Crystal data and summary of the data collection and refinement for [fbfb][Al(OR^F)$_4$].

Compound	**[1][Al(OR^F)$_4$]**
CCDC	2531335
Empirical formula	$C_{28}H_8AlF_{38}O_4$
Formula weight	1157.32
Temperature [K]	100(2)
Crystal system	monoclinic
Space group (number)	$C2/c$ (15)

a [Å]	19.009(7)
b [Å]	10.125(3)
c [Å]	20.210(5)
α [°]	90
β [°]	109.554(7)
γ [°]	90
Volume [$Å^3$]	3665(2)
Z	4
ρ_{calc} [gcm^{-3}]	2.097
μ [mm^{-1}]	0.288
F(000)	2252
Crystal size [mm^3]	0.10×0.08×0.03
Crystal colour	blue
Crystal shape	block
Radiation	MoK_{α} (λ=0.71073 Å)
2θ range [°]	4.28 to 59.37 (0.72 Å)
Index ranges	−26 ≤ h ≤ 26<br>−13 ≤ k ≤ 14<br>−28 ≤ l ≤ 28
Reflections collected	86412
Independent reflections	5141<br>R_{int} = 0.0773<br>R_{sigma} = 0.0399
Completeness to θ = 25.242°	100.0 %
Data / Restraints / Parameters	5141 / 2159 / 448
Absorption correction T_{min}/T_{max} (method)	1.034
Goodness-of-fit on F^2	
Final *R* indexes [$I≥2σ(I)$]	R_1 = 0.0407<br>wR_2 = 0.0866
Final *R* indexes [all data]	R_1 = 0.0809<br>wR_2 = 0.1017
Largest peak/hole [$eÅ^{-3}$]	0.24/-0.27

7.2. [Xe(C_6F_5)][FAl(OR^F)$_3$] and [Xe(C_6F_5)][F(Al(OR^F)$_3$)$_2$]

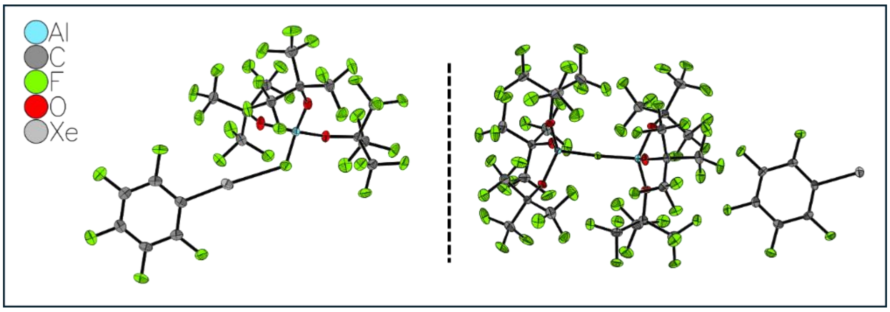

Figure S 47: Molecular structure of [Xe(C_6F_5)][FAl(OR^F)$_3$] (left) and [Xe(C_6F_5)][F(Al(OR^F)$_3$)$_2$] (right). Thermal displacement ellipsoids are shown at 50% probability. The measurements were carried out at 100 K.

Table S 2: Crystal data and summary of the data collection and refinement for [Xe(C_6F_5)][FAl(OR^F)$_3$] and [Xe(C_6F_5)][F(Al(OR^F)$_3$)$_2$].

Compound	**[Xe(C_6F_5)][FAl(OR^F)$_3$]**	**[Xe(C_6F_5)][F(Al(OR^F)$_3$)$_2$]**
CCDC	2529885	2529886
Empirical formula	$C_{18}AlF_{33}O_3Xe$	$C_{30}Al_2F_{60}O_6Xe$

Formula weight	1049.46	1781.56
Temperature [K]	100(2)	100(2)
Crystal system	orthorhombic	monoclinic
Space group (number)	$Pbca$ (61)	$P2_1/n$ (14)
a [Å]	17.8570(18)	13.2484(7)
b [Å]	17.785(4)	16.7851(11)
c [Å]	18.0695(17)	44.788(3)
α [°]	90	90
β [°]	90	95.969(2)
γ [°]	90	90
Volume [Å^3]	5738.5(15)	9905.8(10)
Z	8	8
ρ_{calc} [gcm^{-3}]	2.429	2.389
μ [mm^{-1}]	1.474	1.004
F(000)	3968	6784
Crystal size [mm^3]	0.220×0.195×0.173	0.276×0.230×0.175
Crystal colour	colourless	colourless
Crystal shape	block	block
Radiation	MoK_α (λ=0.71073 Å)	MoK_α (λ=0.71073 Å)
2θ range [°]	3.94 to 56.67 (0.75 Å)	2.59 to 54.32 (0.78 Å)
Index ranges	−23 ≤ h ≤ 23<br>−23 ≤ k ≤ 23<br>−24 ≤ l ≤ 24	−16 ≤ h ≤ 16<br>−21 ≤ k ≤ 21<br>−57 ≤ l ≤ 57
Reflections collected	201923	122388
Independent reflections	7143<br>R_{int} = 0.0524<br>R_{sigma} = 0.0119	21824<br>R_{int} = 0.0289<br>R_{sigma} = 0.0201
Completeness to θ = 25.242°	99.9 %	99.5 %
Data / Restraints / Parameters	7143/1731/505	21824/29515/2037
Goodness-of-fit on F^2	1.030	1.216
Final R indexes [I≥2σ(I)]	R_1 = 0.0195<br>wR_2 = 0.0474	R_1 = 0.0537<br>wR_2 = 0.1205
Final R indexes [all data]	R_1 = 0.0231<br>wR_2 = 0.0493	R_1 = 0.0559<br>wR_2 = 0.1214
Largest peak/hole [eÅ^{-3}]	0.53/-0.58	1.39/-1.10

7.3. [$(H_2O)Xe(C_6F_5)$][$Al(OR^F)_4$]

During a procedure analogous to that used for preparation of [C_6F_5Xe][WCA] (with [WCA]$^-$ = [F-Al$(OR^F)_3$]$^-$, [F(Al$(OR^F)_3)_2$]$^-$), Li[Al$(OR^F)_4$] was employed instead of Me_3SiF-Al$(OR^F)_3$. Crystals suitable for scXRD were obtained and refined as [$C_6F_5Xe(H_2O)$][Al$(OR^F)_4$]. Notably, structural refinement with HF in place of H_2O resulted in slightly increased R-values. Additionally, the experimentally observed Xe···X distance of 2.624(3) Å is closer to the calculated Xe···OH_2 distance (2.68 Å) than to the calculated Xe···FH distance (2.71 Å).

For comparison, recently characterized Xe(VI) hydrate complexes exhibit a similarly short Xe···O(H_2O) contact, e.g. in [(18-crown-6)(H_2O)XeO_3]·H_2O the Xe···O(H_2O) the corresponding distance is 2.702(10) Å. However, the bonding situation in the present organoxenonium species is clearly different from that in XeO_3.[24]

While the differences of the distances in the calculated structures are small, this trend is consistent with the assignment of the coordinated fragment as H_2O rather than HF. However, as only scXRD data are available, independent verification by ^{1}H and ^{19}F NMR was not possible.

Interactions of xenon with water have been previously reported in the gas phase or in noble-gas matrices.[25,26] The present study therefore represents an rare example of xenon-water complex observed at room temperature in the solid state.

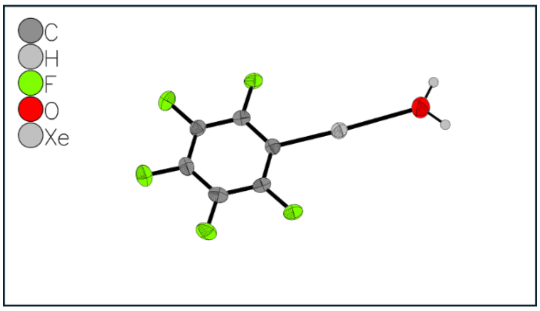

Figure S 48: Molecular structure of the cation in $[(H_2O)Xe(C_6F_5)][Al(OR^F)_4]$. Thermal displacement ellipsoids are shown at 50% probability. The counter ion is omitted for clarity. The measurement was carried out at 100 K.

Table S 3: Crystal data and summary of the data collection and refinement for $[(H_2O)Xe(C_6F_5)][Al(OR^F)_4]$.

Compound	$\mathbf{[(H_2O)Xe(C_6F_5)][Al(OR^F)_4]}$
CCDC	2529884
Empirical formula	$C_{22}H_2AlF_{41}O_5Xe$
Formula weight	1283.52
Temperature [K]	100(2)
Crystal system	monoclinic
Space group (number)	$P2_1/n$ (14)
a [Å]	10.541(3)
b [Å]	20.426(6)
c [Å]	17.295(6)
α [°]	90
β [°]	97.644(10)
γ [°]	90
Volume [Å^3]	3690.6(19)
Z	4
ρ_{calc} [gcm^{-3}]	2.310
μ [mm^{-1}]	1.203
F(000)	2440
Crystal size [mm^3]	0.075×0.098×0.116
Crystal colour	colourless
Crystal shape	block
Radiation	MoK_α (λ=0.71073 Å)
2θ range [°]	3.10 to 52.81 (0.80 Å)
Index ranges	−13 ≤ h ≤ 13<br>−25 ≤ k ≤ 25<br>−21 ≤ l ≤ 21
Reflections collected	117085
Independent reflections	7580<br>R_{int} = 0.0827<br>R_{sigma} = 0.0311
Completeness to θ = 25.242°	100.0 %

Data / Restraints / Parameters	7580 / 3093 / 641
Absorption correction T_{min}/T_{max} (method)	0.5620 / 0.7454 (multi-scan)
Goodness-of-fit on F^2	1.115
Final *R* indexes [$I \geq 2\sigma(I)$]	$R_1 = 0.0432$<br>$wR_2 = 0.1097$
Final *R* indexes [all data]	$R_1 = 0.0512$<br>$wR_2 = 0.1158$
Largest peak/hole [eÅ^{-3}]	1.04/−1.29

8. IR Spectroscopy

8.1. C_6F_5Xe-F-Al(OR^F)$_3$

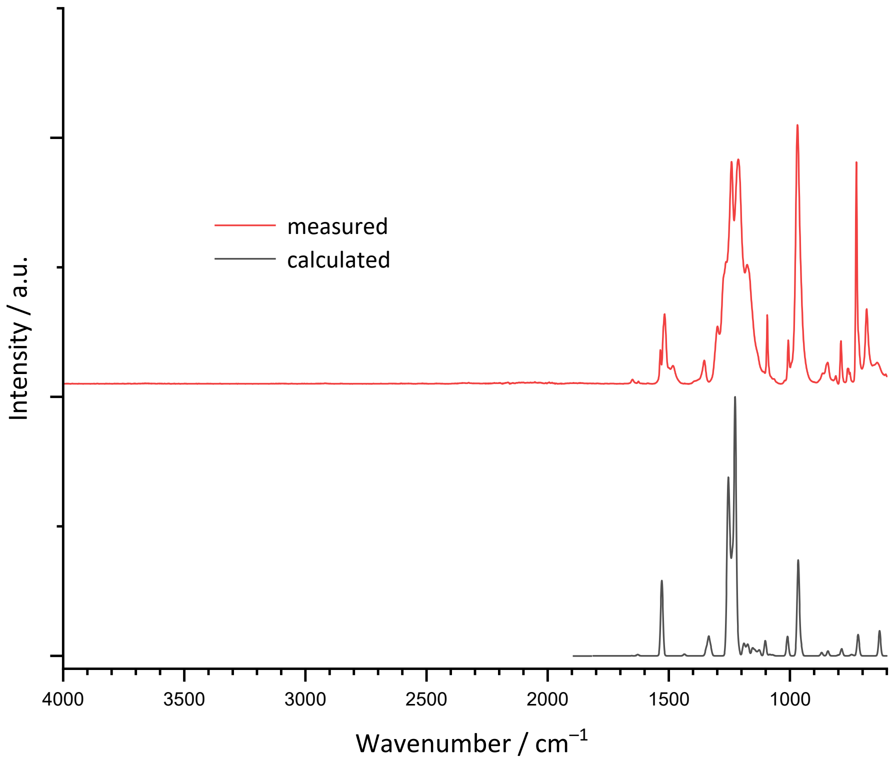

Figure S 49: Comparison of the experimental (red, C_6F_5Xe-F-Al(OR^F)$_3$) and the calculated (black, C_6F_5Xe-F-Al(OR^F)$_3$, RI-r2scan-3c(D4)/def2-mTZVPP) IR spectra.

FT-IR (diamond, ATR): 1650 (vw), 1625 (vw), 1535 (vw), 1517 (w), 1483 (vw), 1354 (vw), 1299 (w), 1241 (vs), 1213 (vs), 1177 (m), 1093 (w), 1007 (w), 969 (vs), 864 (vw), 844 (vw), 789 (w), 762 (vw), 726 (vs), 683 (w), 640 (vw), 574 (vw), 566 (w), 536 (w), 491 (vw), 450 (w), 377 (w).

8.2. $[C_6F_5Xe][F(Al(OR^F)_3)_2]$

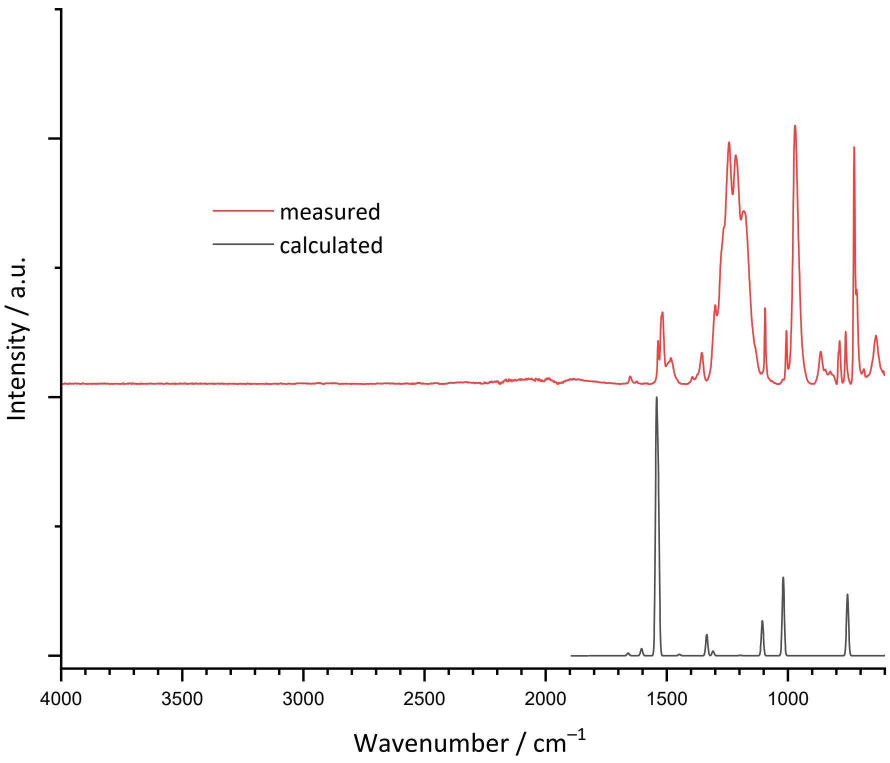

Figure S 50: Comparison of the experimental (red, $[C_6F_5Xe][F(Al(OR^F)_3)_2]$) and the calculated (black, $[C_6F_5Xe]^+$, RI-r2scan-3c(D4)/def2-mTZVPP) IR spectra. The IR bands of the anion can be compared with the anion bands from the IR spectrum of NO[al-f-al] (see ESI 8.3).

FT-IR (diamond, ATR): 1651 (vw), 1624 (vw), 1536 (vw), 1517 (w), 1490 (vw), 1394 (vw), 1355 (vw), 1300 (w), 1243 (vs), 1216 (vs), 1182 (s), 1094 (w), 1006 (w), 970 (vs), 864 (vw), 824 (vw), 786 (vw), 762 (w), 726 (vs), 716 (w), 687 (vw), 636 (vw), 571 (vw), 537 (w), 449 (w).

8.3. [NO][F(Al(OR^F)$_3$)$_2$]

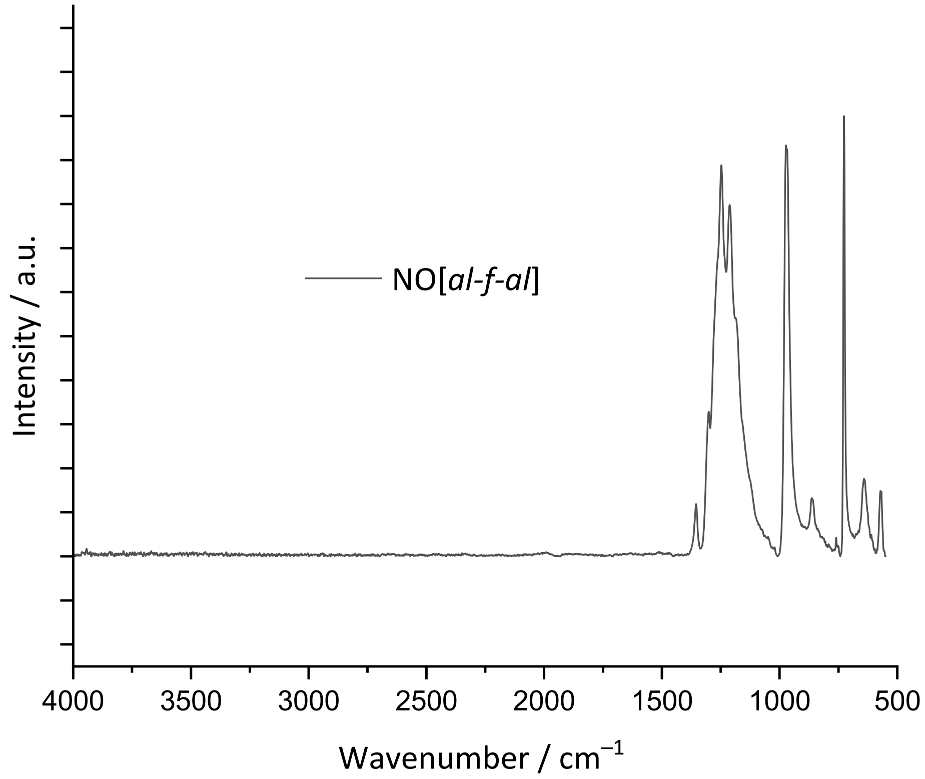

Figure S 51: IR spectrum of NO[F(Al(OR^F)$_3$)$_2$].

FT-IR [ZnSe, ATR] = 1355 (vw), 1301 (w), 1247 (vs), 1212 (s), 1187 (m), 968 (vs), 863 (vw), 760 (vw), 726 (vs), 641 (vw), 572 (vw)

9. Quantum chemical Calculations

9.1. Benzene

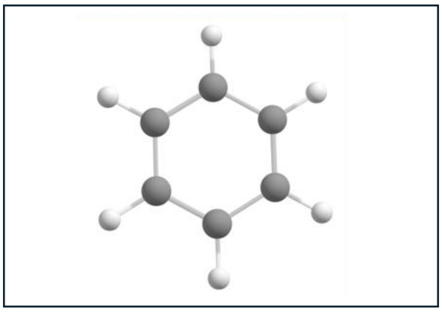

Total enthalpy: -232.07277391 Eh

Total entropy: 0.03283248 Eh

Final Gibbs free energy: -232.10560638 Eh

Cartesian coordinates in Ångström:

Benzene

atom	x-value	y-value	z-value
H	-0.95896	-0.81915	1.94909
C	-1.47411	-0.37314	1.10416
C	-2.86046	-0.24532	1.12254
C	-3.52126	0.32662	0.03867
C	-2.79577	0.77080	-1.06356
H	-3.31098	1.21676	-1.90849
C	-1.40944	0.64302	-1.08197
H	-0.84382	0.98929	-1.94130
C	-0.74861	0.07104	0.00193
H	0.33215	-0.02866	-0.01253
H	-4.60203	0.42636	0.05302
H	-3.42597	-0.59155	1.98193

9.2. $[C_6H_5]^+$

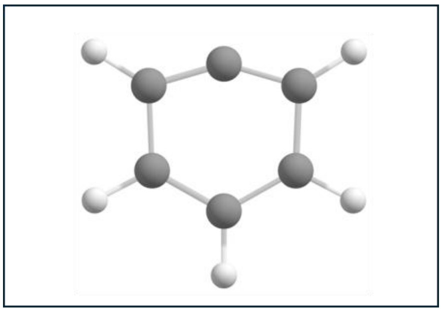

Total enthalpy: -231.10136330 Eh

Total entropy: 0.03343621 Eh

Final Gibbs free energy: -231.13479952 Eh

Cartesian coordinates in Ångström:

$[C_6H_5]^+$

atom	x-value	y-value	z-value
H	-1.00191	-0.82728	1.97461
C	-1.47591	-0.37411	1.10661
C	-2.90420	-0.26537	1.17763
C	-3.23654	0.30209	0.03557
C	-2.83614	0.80106	-1.11669
H	-3.37973	1.24132	-1.94276
C	-1.41093	0.64436	-1.08466
H	-0.88515	1.00446	-1.96637
C	-0.76425	0.07194	0.00175
H	0.31545	-0.02855	-0.01293
H	-3.49717	-0.59998	2.01918

9.3. Biphenyl

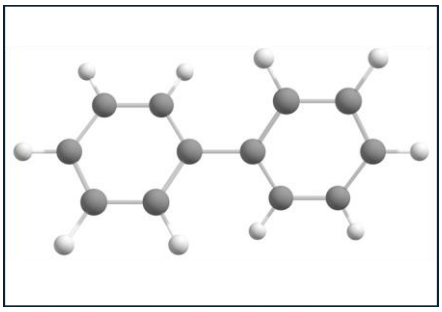

Total enthalpy: -462.98441400 Eh

Total entropy: 0.04327367 Eh

Final Gibbs free energy: -463.02768768 Eh

Cartesian coordinates in Ångström:

Biphenyl

atom	x-value	y-value	z-value
H	3.55105	1.69730	0.75563
C	2.93593	0.86776	0.42124
C	1.55189	0.99364	0.41975
H	1.09277	1.91309	0.76968
C	0.73558	-0.06662	0.00586
C	1.34853	-1.25634	-0.40722
H	0.73240	-2.07819	-0.75873
C	2.73267	-1.38127	-0.40654
H	3.18912	-2.30793	-0.74014
C	3.53247	-0.32011	0.00802
H	-1.09277	-1.91309	0.76968
C	-1.55189	-0.99364	0.41975
C	-2.93593	-0.86776	0.42124
H	-3.55105	-1.69730	0.75563
C	-3.53247	0.32010	0.00802
C	-2.73267	1.38127	-0.40654
H	-3.18912	2.30793	-0.74014
C	-1.34853	1.25634	-0.40722
H	-0.73240	2.07819	-0.75873
C	-0.73558	0.06662	0.00586
H	-4.61311	0.41814	0.00896
H	4.61310	-0.41814	0.00896

9.4. [Biphenyl]$^{+}$

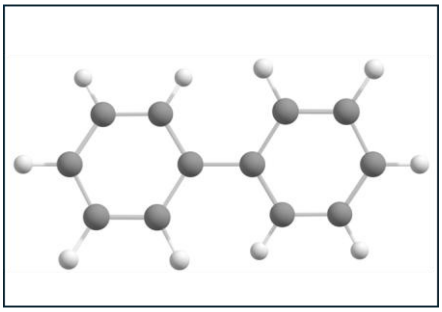

Total enthalpy: -462.69680360 Eh

Total entropy: 0.04442741 Eh

Final Gibbs free energy: -462.74123101 Eh

Cartesian coordinates in Ångström:

[Biphenyl]$^+$

atom	x-value	y-value	z-value
H	3.54987	1.78786	0.48519
C	2.92205	0.93101	0.26822
C	1.55368	1.06279	0.25720
H	1.11074	2.02290	0.49163
C	0.71712	-0.06499	0.00842
C	1.33717	-1.32474	-0.24125
H	0.72872	-2.19001	-0.47378
C	2.70697	-1.44054	-0.25523
H	3.17043	-2.39612	-0.47319
C	3.50908	-0.31753	0.00542
H	-1.11074	-2.02290	0.49163
C	-1.55368	-1.06279	0.25720
C	-2.92206	-0.93101	0.26822
H	-3.54987	-1.78786	0.48519
C	-3.50908	0.31753	0.00542
C	-2.70697	1.44054	-0.25523
H	-3.17043	2.39612	-0.47318
C	-1.33717	1.32474	-0.24125
H	-0.72872	2.19001	-0.47378
C	-0.71712	0.06499	0.00842
H	-4.58981	0.41524	0.00403
H	4.58981	-0.41524	0.00402

9.5. $[C_6H_6\text{-}C_6H_5]^+$

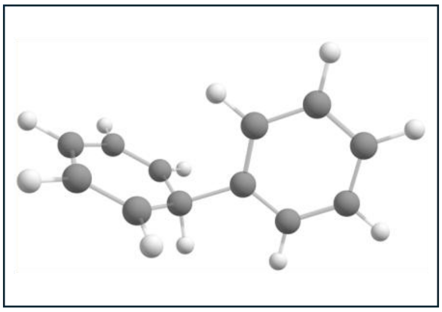

Total enthalpy: -463.26944713 Eh

Total entropy: 0.04556198 Eh

Final Gibbs free energy: -463.31500911 Eh

Cartesian coordinates in Ångström:

$[C_6H_6\text{-}C_6H_5]^+$

atom	x-value	y-value	z-value
C	-3.79234	2.04070	1.42748
C	-4.40165	2.42627	0.21999
C	-2.96514	0.95065	1.43468

C	-4.18692	1.74802	-0.99327
C	-2.71562	0.17042	0.21772
C	-3.36288	0.65565	-1.00611
C	-3.01029	-1.33952	0.44959
H	-4.67856	2.08935	-1.89722
H	-3.17876	0.09930	-1.92093
C	-4.32483	-1.72506	0.71061
C	-1.98517	-2.27844	0.39913
C	-2.28376	-3.62137	0.61358
C	-4.61085	-3.06873	0.92311
C	-3.59168	-4.01572	0.87483
H	-5.63172	-3.37351	1.12668
H	-0.96204	-1.97744	0.19583
H	-1.48787	-4.35723	0.57530
H	-5.12053	-0.98614	0.74867
H	-3.98865	2.60095	2.33458
H	-2.48291	0.61514	2.34859
H	-1.62146	0.19958	0.03599
H	-3.81792	-5.06316	1.04129
H	-5.06651	3.28643	0.22427

9.6. Fluorobenzene

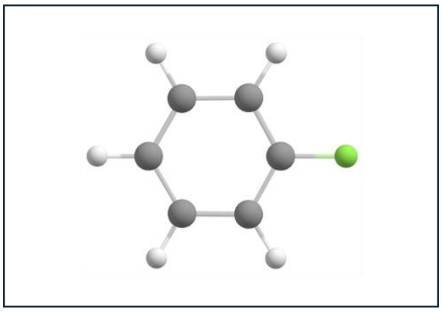

Total enthalpy: -331.32508168 Eh

Total entropy: 0.03496424 Eh

Final Gibbs free energy: -331.36004592 Eh

Cartesian coordinates in Ångström:

Fluorobenzene

atom	x-value	y-value	z-value
H	-0.96509	-0.81908	1.95000
C	-1.47759	-0.37276	1.10411
C	-2.86394	-0.24973	1.13300
C	-3.49418	0.32325	0.04065
F	-4.84121	0.44716	0.05966
C	-2.80082	0.77458	-1.07034
H	-3.34147	1.21586	-1.89987
C	-1.41500	0.64292	-1.08053
H	-0.85362	0.99047	-1.94164
C	-0.75150	0.07126	0.00205
H	-3.45236	-0.58713	1.97857
H	0.32827	-0.02803	-0.01322

9.7. $[C_6H_4F]^+$

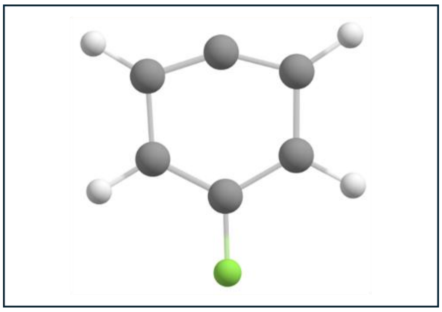

Total enthalpy: -330.33789001 Eh

Total entropy: 0.03586418 Eh

Final Gibbs free energy: -330.37375418 Eh

Cartesian coordinates in Ångström:

Name			
atom	x-value	y-value	z-value
H	-0.99647	-0.82650	1.97286
C	-1.50385	-0.37842	1.12171
C	-2.92141	-0.26566	1.18130
C	-3.25167	0.30566	0.03677
C	-2.85306	0.80405	-1.12011
H	-3.40795	1.24229	-1.94087
C	-1.43790	0.65376	-1.09911
H	-0.87983	1.00352	-1.96452
C	-0.80675	0.07559	0.00217
F	0.50772	-0.04731	-0.01576
H	-3.52529	-0.59704	2.01748

9.8. 4,4’-Difluorobiphenyl

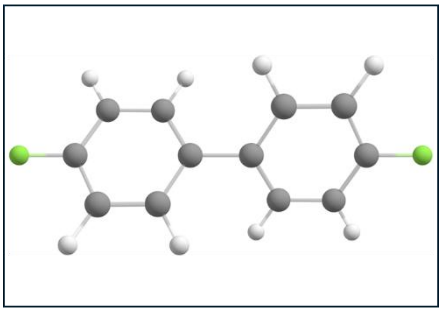

Total enthalpy: -661.48799310 Eh

Total entropy: 0.04705489 Eh

Final Gibbs free energy: -661.53504798 Eh

Cartesian coordinates in Ångström:

4,4‘-Difluorobiphenyl			
atom	x-value	y-value	z-value

H	3.57531	1.68818	0.75623
C	2.93703	0.87682	0.42520
C	1.55239	0.99234	0.42208
H	1.09669	1.91190	0.77517
C	0.73523	-0.06659	0.00594
C	1.34923	-1.25515	-0.40945
H	0.73642	-2.07769	-0.76412
C	2.73211	-1.39037	-0.41052
H	3.21455	-2.30329	-0.74086
C	3.50081	-0.31722	0.00795
F	4.84696	-0.43931	0.00902
H	-1.09669	-1.91190	0.77517
C	-1.55239	-0.99234	0.42208
C	-2.93703	-0.87682	0.42520
H	-3.57531	-1.68818	0.75623
C	-3.50081	0.31722	0.00795
F	-4.84696	0.43931	0.00902
C	-2.73211	1.39037	-0.41051
H	-3.21455	2.30329	-0.74086
C	-1.34923	1.25515	-0.40945
H	-0.73642	2.07769	-0.76412
C	-0.73523	0.06659	0.00594

9.9. [4,4’-Difluorobiphenyl]$^+$

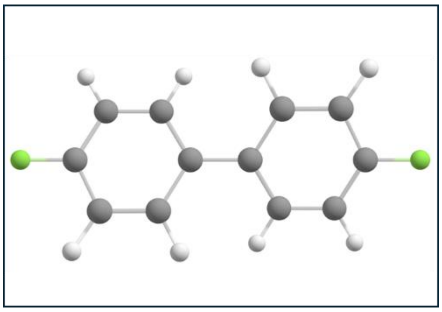

Total enthalpy: -661.20091933 Eh

Total entropy: 0.04810768 Eh

Final Gibbs free energy: -661.24902702 Eh

Cartesian coordinates in Ångström:

[4,4’-Difluorobiphenyl]$^+$

atom	x-value	y-value	z-value
H	3.57173	1.78228	0.48499
C	2.92126	0.94216	0.26987
C	1.55408	1.06107	0.25854
H	1.11372	2.02195	0.49482
C	0.71739	-0.06502	0.00805
C	1.33794	-1.32312	-0.24311
H	0.73196	-2.18958	-0.47784
C	2.70425	-1.45145	-0.25663
H	3.19309	-2.39467	-0.47241
C	3.48054	-0.31500	0.00581
F	4.79384	-0.43379	0.00455
H	-1.11372	-2.02195	0.49482

C	-1.55408	-1.06107	0.25854
C	-2.92126	-0.94216	0.26987
H	-3.57173	-1.78228	0.48499
C	-3.48054	0.31500	0.00581
F	-4.79384	0.43378	0.00455
C	-2.70425	1.45145	-0.25663
H	-3.19309	2.39467	-0.47241
C	-1.33794	1.32312	-0.24311
H	-0.73196	2.18958	-0.47784
C	-0.71739	0.06502	0.00805

9.10. $[C_6H_5F\text{-}C_6H_4F]^+$

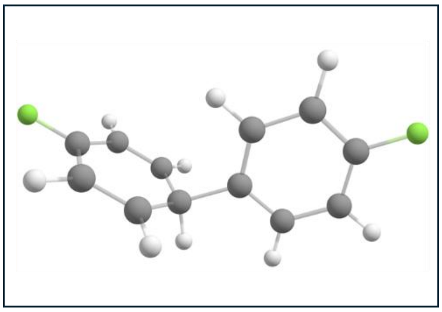

Total enthalpy: -661.77346756 Eh

Total entropy: 0.04917045 Eh

Final Gibbs free energy: -661.82263801 Eh

Cartesian coordinates in Ångström:

$[C_6H_5F\text{-}C_6H_4F]^+$

atom	x-value	y-value	z-value
C	-3.79764	2.02486	1.44446
C	-4.39713	2.39019	0.22323
C	-2.96201	0.95075	1.43663
C	-4.19344	1.73097	-1.00497
C	-2.70024	0.16984	0.21498
C	-3.35758	0.65707	-1.01025
C	-3.00558	-1.32980	0.44678
H	-4.70391	2.09095	-1.89147
H	-3.16816	0.10709	-1.92772
F	-5.20250	3.41858	0.22989
C	-4.32167	-1.71507	0.70983
C	-1.98691	-2.27901	0.39708
C	-2.27961	-3.62016	0.61140
C	-4.62469	-3.05177	0.92538
C	-3.59382	-3.98067	0.87230
F	-3.87847	-5.26823	1.08121
H	-5.63690	-3.37951	1.13226
H	-0.96189	-1.98554	0.19278
H	-1.50744	-4.38018	0.57923
H	-5.11755	-0.97645	0.74884
H	-4.02036	2.59816	2.33759
H	-2.47627	0.62025	2.35039
H	-1.60805	0.20801	0.03409

9.11. 1,2-Difluorobenzene

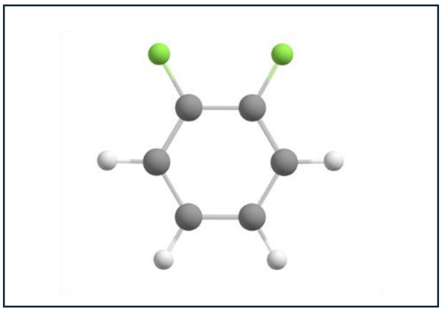

Method: (RI-)r2scan-3c(D4)/def2-mTZVPP

Total enthalpy: -430.56952670 Eh

Total entropy: 0.03711345 Eh

Final Gibbs free energy: -430.60664015 Eh

Cartesian coordinates in Ångström:

o-$C_6H_4F_2$

atom	x-value	y-value	z-value
H	-0.97132	-0.81761	1.94793
C	-1.46002	-0.36865	1.09038
C	-2.83740	-0.23638	1.09860
C	-3.49870	0.33444	0.01624
F	-4.83722	0.44898	0.05384
C	-2.78947	0.77995	-1.08528
H	-3.33198	1.22141	-1.91392
C	-1.40274	0.64986	-1.09842
H	-0.84242	0.99795	-1.95892
C	-0.74130	0.07805	-0.01606
F	-3.55409	-0.65620	2.15515
H	0.33816	-0.02301	-0.02711

9.12. $[C_6H_3F_2]^+$

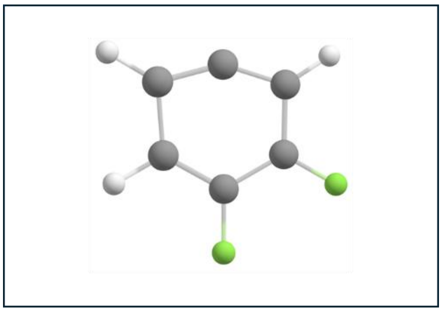

Method: (RI-)r2scan-3c(D4)/def2-mTZVPP

Total enthalpy: -429.57286920 Eh

Total entropy: 0.03939743 Eh

Final Gibbs free energy: -429.61226663 Eh

Cartesian coordinates in Ångström:

$[C_6H_3F_2]^+$

atom	x-value	y-value	z-value
F	-0.89097	-0.91619	2.13400
C	-1.50628	-0.37617	1.11824
C	-2.91863	-0.25018	1.15146
C	-3.26750	0.32045	0.00593
C	-2.88878	0.82179	-1.14884
H	-3.41178	1.26613	-1.98491
C	-1.46600	0.64838	-1.08262
H	-0.90912	0.99951	-1.95106
C	-0.79961	0.08041	-0.00928
F	0.51016	-0.04196	-0.02766
H	-3.52797	-0.58223	1.98667

9.13. 3,3’4,4’-Tetrafluorobiphenyl

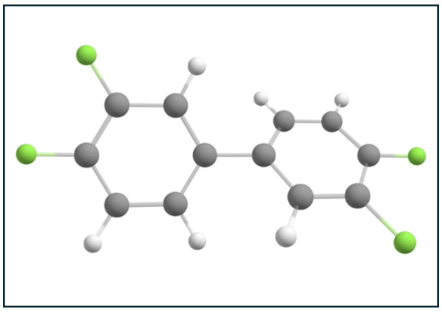

Method: (RI-)r2scan-3c(D4)/def2-mTZVPP

Total enthalpy: -859.97655579 Eh

Total entropy: 0.05166182 Eh

Final Gibbs free energy: -860.02821760 Eh

Cartesian coordinates in Ångström:

3,3’4,4’-Tetrafluorobiphenl

atom	x-value	y-value	z-value
H	3.46650	0.24262	2.02807
C	2.87371	0.05230	1.14031
C	1.48875	0.17094	1.15019
H	0.99013	0.47968	2.06236
C	0.73535	-0.06346	-0.00613
C	1.40366	-0.42178	-1.18416
H	0.86106	-0.64596	-2.09591
C	2.78050	-0.53401	-1.18870
F	3.42496	-0.88705	-2.31291
C	3.51709	-0.30072	-0.03236
F	4.85357	-0.42098	-0.06849
H	-0.99461	-0.84452	1.96111
C	-1.47466	-0.37021	1.11224
C	-2.84953	-0.23603	1.11927
F	-3.56320	-0.66144	2.17448
C	-3.51496	0.32505	0.03463
F	-4.85203	0.43881	0.07052

C	-2.80087	0.76124	-1.06698
H	-3.33667	1.20835	-1.89691
C	-1.41674	0.63321	-1.07804
H	-0.85694	1.00645	-1.92851
C	-0.73507	0.06752	0.00593

9.14. [3,3’4,4’-Tetrafluorobiphenl]$^{+}$

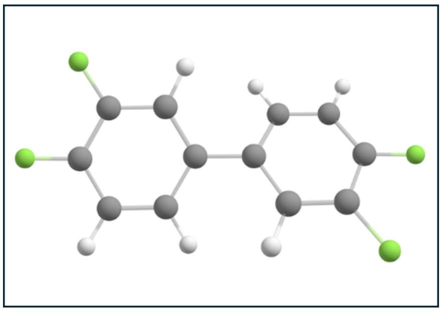

Method: (RI-)r2scan-3c(D4)/def2-mTZVPP

Total enthalpy: -859.68033455 Eh

Total entropy: 0.05266314 Eh

Final Gibbs free energy: -859.73299769 Eh

Cartesian coordinates in Ångström:

[3,3’4,4’-tetrafluorobiphenl]$^{+}$

atom	x-value	y-value	z-value
H	3.43683	0.02098	2.09105
C	2.84754	-0.07508	1.18597
C	1.47607	0.02651	1.19181
H	0.97387	0.23397	2.12850
C	0.71894	-0.07075	-0.01439
C	1.40252	-0.28468	-1.23440
H	0.87946	-0.41275	-2.17423
C	2.76723	-0.40544	-1.23418
F	3.43454	-0.63920	-2.34944
C	3.49847	-0.29757	-0.02709
F	4.80462	-0.41010	-0.07293
H	-0.97334	-0.62947	2.08047
C	-1.45031	-0.24622	1.18664
C	-2.81464	-0.12159	1.18668
F	-3.53073	-0.42705	2.25341
C	-3.49583	0.32613	0.02955
F	-4.80187	0.43989	0.07541
C	-2.79651	0.64157	-1.13501
H	-3.34500	0.99937	-1.99921
C	-1.42879	0.49844	-1.14451
H	-0.88273	0.77678	-2.03724
C	-0.72033	0.05626	0.01315

9.15. [$C_6F_2H_4$-$C_6H_3F_2$]$^+$

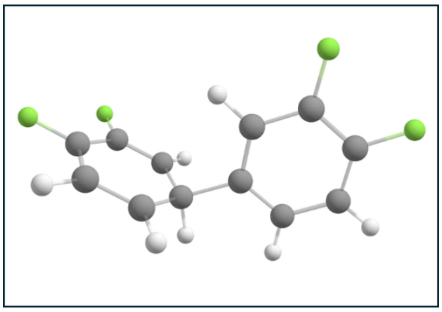

Method: (RI-)r2scan-3c(D4)/def2-mTZVPP

Total enthalpy: -860.24948305 Eh

Total entropy: 0.05313709 Eh

Final Gibbs free energy: -860.30262015 Eh

Cartesian coordinates in Ångström:

[$C_6F_2H_4$-$C_6H_3F_2$]$^+$

atom	x-value	y-value	z-value
C	-3.78386	2.03881	1.40963
C	-4.38231	2.42114	0.17848
C	-2.97152	0.95314	1.44602
C	-4.17116	1.73499	-1.02878
C	-2.71398	0.16438	0.22795
C	-3.35447	0.64407	-1.00806
C	-3.00065	-1.33924	0.45114
H	-4.66437	2.09217	-1.92668
H	-3.16400	0.07823	-1.91536
F	-5.16075	3.46091	0.18769
C	-4.31202	-1.72941	0.73112
C	-1.97738	-2.27776	0.37072
C	-2.26070	-3.62547	0.57541
C	-4.58121	-3.07088	0.93314
C	-3.55785	-4.01753	0.85576
F	-3.84870	-5.29738	1.05785
F	-5.82143	-3.46786	1.20474
H	-0.95789	-1.97676	0.15393
H	-1.48300	-4.37931	0.52292
H	-5.13125	-1.02047	0.80073
F	-4.06584	2.76138	2.47903
H	-2.51687	0.63948	2.38148
H	-1.62067	0.21371	0.04510

9.16. 1,2,3-Trifluorobenzene

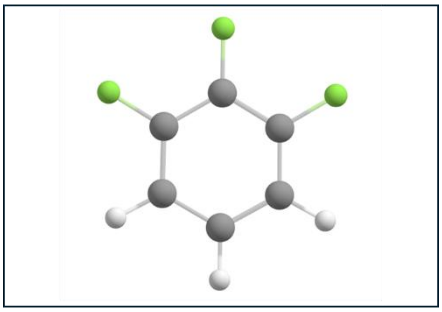

Method: (RI-)r2scan-3c(D4)/def2-mTZVPP

Total enthalpy: -529.81306281 Eh

Total entropy: 0.03926182 Eh

Final Gibbs free energy: -529.85232463 Eh

Cartesian coordinates in Ångström:

1,2,3-Trifluorobenzene

atom	x-value	y-value	z-value
F	-0.86149	-0.90691	2.10923
C	-1.47687	-0.35029	1.05485
C	-2.86167	-0.22896	1.08907
C	-3.50863	0.34387	-0.00041
F	-4.84505	0.45607	0.04121
C	-2.80046	0.79007	-1.10377
H	-3.34114	1.23094	-1.93289
C	-1.41511	0.65827	-1.11268
H	-0.85118	1.00463	-1.97101
C	-0.74374	0.08783	-0.03541
F	-3.55718	-0.65272	2.14817
H	0.33400	-0.02402	-0.02392

9.17. $[C_6H_2F_3]^+$

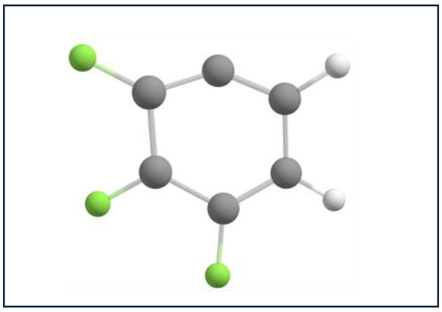

Total enthalpy: -528.78971400 Eh

Total entropy: 0.03959937 Eh

Final Gibbs free energy: -528.82931337 Eh

Cartesian coordinates in Ångström:

$[C_6H_2F_3]^+$

atom	x-value	y-value	z-value
F	-0.95264	-0.94947	1.99609
C	-1.54667	-0.21657	1.10057
C	-2.97162	-0.12796	1.19174
C	-3.35461	0.09470	-0.07793
C	-2.80086	0.70642	-1.12771
H	-3.34010	1.43928	-1.73101
C	-1.39327	0.55715	-1.23758
H	-0.83014	0.94283	-2.08056
C	-0.78955	0.14959	-0.06938
F	0.50896	0.03815	0.03929
F	-3.60597	-0.66416	2.18840

9.18. 2,2’,3,3’,4,4’-Hexafluorobiphenyl

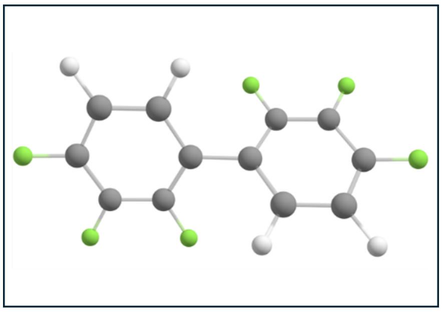

Total enthalpy: -1058.45988589 Eh

Total entropy: 0.05493615 Eh

Final Gibbs free energy: -1058.51482203 Eh

Cartesian coordinates in Ångström:

2,2’,3,3’,4,4’-Hexafluorobiphenyl

atom	x-value	y-value	z-value
C	2.85787	-1.09039	0.51863
C	1.46994	-1.09051	0.50311
H	0.93372	-1.94544	0.89696
C	0.73771	-0.00631	0.00399
C	1.45866	1.08237	-0.48882
F	0.82626	2.14411	-1.01196
C	2.84812	1.10350	-0.48184
F	3.50650	2.15931	-0.96727
C	3.53827	0.01004	0.02567
F	4.87801	0.04429	0.02885
F	-0.82626	-2.14411	-1.01196
C	-1.45866	-1.08237	-0.48882
C	-2.84812	-1.10350	-0.48184
F	-3.50650	-2.15931	-0.96727
C	-3.53827	-0.01004	0.02567
F	-4.87801	-0.04429	0.02886
C	-2.85787	1.09039	0.51863
C	-1.46994	1.09051	0.50311
H	-0.93372	1.94544	0.89697
C	-0.73771	0.00631	0.00399
H	-3.42217	1.92771	0.91180
H	3.42217	-1.92771	0.91179

9.19. [2,2’,3,3’,4,4’-Hexafluorobiphenyl]$^{+}$

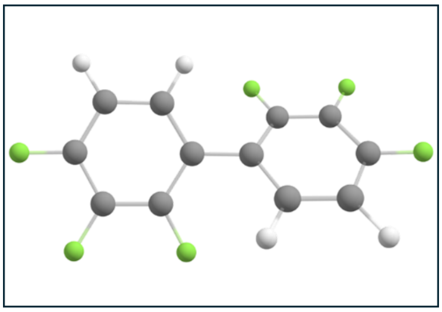

Total enthalpy: -1058.15431452 Eh

Total entropy: 0.05569348 Eh

Final Gibbs free energy: -1058.21000800 Eh

Cartesian coordinates in Ångström:

[2,2’,3,3’,4,4’-Hexafluorobiphenyl]$^{+}$

atom	x-value	y-value	z-value
C	2.83257	-1.15309	0.40237
C	1.46301	-1.16450	0.33456
H	0.93009	-2.05622	0.63434
C	0.72064	-0.00462	-0.04680
C	1.45839	1.16059	-0.38761
F	0.83832	2.25034	-0.82091
C	2.83702	1.17938	-0.34695
F	3.51192	2.25385	-0.69742
C	3.51949	0.01599	0.05375
F	4.82906	0.05172	0.08908
F	-0.83832	-2.25034	-0.82092
C	-1.45839	-1.16059	-0.38761
C	-2.83702	-1.17938	-0.34695
F	-3.51192	-2.25385	-0.69742
C	-3.51949	-0.01599	0.05376
F	-4.82906	-0.05171	0.08908
C	-2.83256	1.15309	0.40237
C	-1.46301	1.16449	0.33455
H	-0.93009	2.05621	0.63434
C	-0.72064	0.00461	-0.04680
H	-3.39940	2.01906	0.72471
H	3.39940	-2.01906	0.72471

9.20. $[C_6H_3F_3\text{-}C_6H_2F_3]^+$

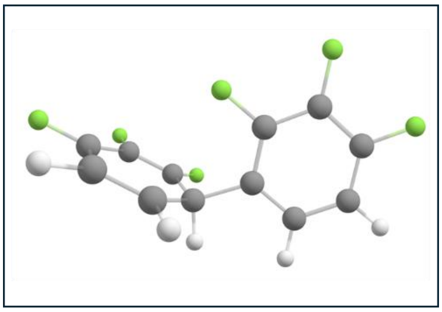

Total enthalpy: -1058.73706107 Eh

Total entropy: 0.05658792 Eh

Final Gibbs free energy: -1058.79364899 Eh

Cartesian coordinates in Ångström:

$[C_6H_3F_3\text{-}C_6H_2F_3]^+$

atom	x-value	y-value	z-value
C	-3.81068	2.06705	1.39724
C	-4.37631	2.44329	0.16415
C	-3.02232	0.94553	1.42078
C	-4.17137	1.74053	-1.04301
C	-2.72856	0.14419	0.21253
C	-3.38354	0.63762	-1.02367
C	-2.96849	-1.34962	0.43801
H	-4.65742	2.10495	-1.94162
H	-3.20136	0.06395	-1.92702
F	-5.13422	3.49950	0.15777
C	-4.26591	-1.76298	0.72667
C	-1.95398	-2.30053	0.35950
C	-2.23759	-3.64467	0.56736
C	-4.56948	-3.09881	0.94251
C	-3.53681	-4.03370	0.85574
F	-3.82942	-5.31034	1.06293
F	-5.80896	-3.46955	1.22311
H	-0.93551	-1.99751	0.14212
H	-1.46239	-4.40035	0.51672
F	-5.24234	-0.84221	0.81419
F	-4.07108	2.76570	2.48136
F	-2.49629	0.54848	2.54446
H	-1.63781	0.24980	0.04211

9.21. H_2

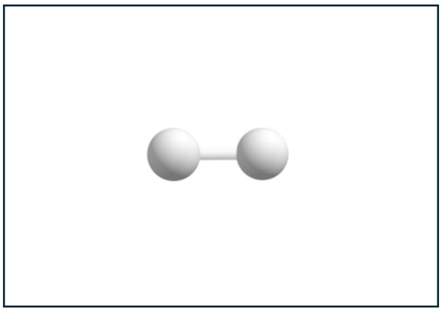

Total enthalpy: -1.15584039 Eh

Total entropy: 0.01544350 Eh

Final Gibbs free energy: -1.17128390 Eh

Cartesian coordinates in Ångström:

H_2

atom	x-value	y-value	z-value
H	-4.78279	0.43881	0.07052
H	-4.04127	0.43881	0.07052

9.22. 1,2,3-Trimethylbenzene

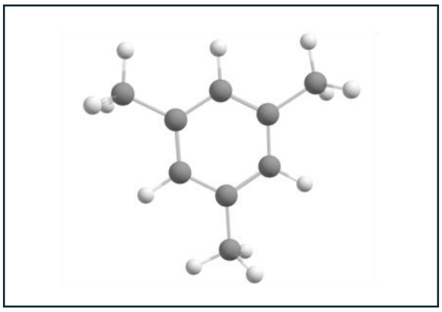

Total enthalpy: -349.89787712 Eh

Total entropy: 0.04009011 Eh

Final Gibbs free energy: -349.93796724 Eh

Cartesian coordinates in Ångström:

1,2,3-Trimethylbenzene

atom	x-value	y-value	z-value
C	-0.76795	-1.01540	2.28239
C	-1.46972	-0.38555	1.10912
C	-2.85985	-0.25038	1.11101
C	-3.53385	0.32713	0.03625
C	-2.78996	0.77514	-1.05772
H	-3.30488	1.22737	-1.90194
C	-1.40158	0.65459	-1.08936
C	-0.60513	1.14127	-2.26997
C	-0.75533	0.07117	0.00313
H	0.32792	-0.02833	-0.01039
C	-5.03310	0.46144	0.06175
H	-3.42809	-0.60356	1.96951
H	0.31477	-1.03306	2.13633
H	-1.10579	-2.04619	2.43495
H	-0.97665	-0.46603	3.20667
H	-5.40615	0.93241	-0.85093
H	-5.35909	1.06808	0.91348
H	-5.51398	-0.51791	0.15831
H	-1.25471	1.55503	-3.04509
H	-0.02292	0.32690	-2.71445
H	0.10408	1.92083	-1.97107

9.23. 1,3,5-trimethylcyclohexa-2,4-dien-1-ylium

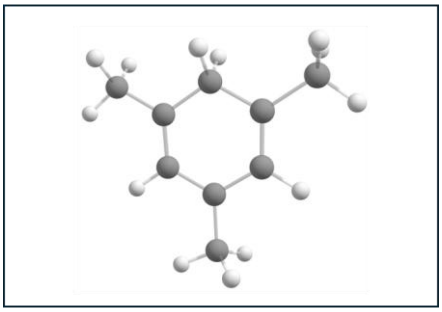

Total enthalpy: -350.22043949 Eh

Total entropy: 0.04395555 Eh

Final Gibbs free energy: -350.26439504 Eh

Cartesian coordinates in Ångström:

1,3,5-trimethylcyclohexa-2,4-dien-1-ylium

atom	x-value	y-value	z-value
C	-0.75113	-0.98453	2.30855
C	-1.43406	-0.37017	1.14200
C	-2.91101	-0.23776	1.18282
C	-3.59309	0.36763	0.01386
C	-2.84352	0.78495	-1.05191
H	-3.32918	1.23063	-1.91399
C	-1.43762	0.64793	-1.05904
C	-0.64880	1.10892	-2.22657
C	-0.75774	0.07196	0.04095
H	0.32367	-0.01650	-0.00351
C	-5.07090	0.49756	0.04992
H	0.33117	-1.00870	2.17930
H	-1.10872	-2.00980	2.46531
H	-0.98506	-0.43417	3.22781
H	-5.46096	0.96005	-0.85698
H	-5.38350	1.09642	0.91423
H	-5.54022	-0.48619	0.17433
H	-1.26975	1.52036	-3.02174
H	-0.05226	0.27820	-2.62468
H	0.07688	1.86801	-1.90640
H	-3.35532	-1.22766	1.38431
H	-3.19265	0.32677	2.08872

9.24. $Al(OR^F)_3$

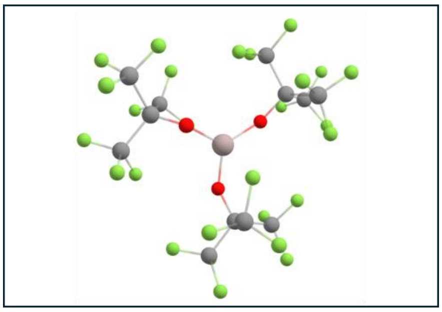

Total enthalpy: -3621.10359337 Eh

Total entropy: 0.10909156 Eh

Final Gibbs free energy: -3621.21268493 Eh

Cartesian coordinates in Ångström:

$Al(OR^F)_3$

atom	x-value	y-value	z-value
Al	-1.00593	-0.64302	0.16351
O	-2.09251	0.64797	-0.01758
O	0.35606	-0.64277	1.25079
O	-1.33391	-2.05313	-0.80598
C	-2.45215	1.95399	0.09777
C	0.37961	-1.35737	2.41613
C	-0.91373	-2.18458	-2.09917
C	-0.57146	-3.68509	-2.39490
C	0.38048	-1.32172	-2.30466
C	-2.04065	-1.68800	-3.07403
F	0.13153	-4.21319	-1.38813
F	0.15851	-3.78938	-3.52507
F	-1.69073	-4.40505	-2.54487
F	-3.22185	-2.18417	-2.70191
F	-1.80468	-2.03994	-4.34848
F	-2.12057	-0.34254	-3.01470
F	1.47825	-1.91355	-1.85896
F	0.21508	-0.17490	-1.46296
F	0.58322	-0.85764	-3.52137
C	0.94923	-2.79664	2.14261
C	-1.08263	-1.47878	2.97415
C	1.27326	-0.61998	3.47009
F	2.57165	-0.78654	3.18319
F	1.04457	-1.10665	4.70759
F	1.01174	0.68934	3.47444
F	-1.92784	-1.53047	1.81840
F	-1.48067	-0.41056	3.64487
F	-1.34794	-2.56567	3.67247
F	0.01968	-3.52498	1.48872
F	1.27185	-3.44409	3.27303
F	2.03175	-2.72523	1.36433
C	-3.95478	2.08046	-0.34433
C	-1.54737	2.84051	-0.82883
C	-2.30034	2.42645	1.58638
F	-1.41974	2.27752	-2.03910
F	-0.30768	2.93715	-0.30303
F	-2.03784	4.07752	-0.98741
F	-4.06243	1.99403	-1.68094
F	-4.49001	3.24974	0.04656
F	-4.67715	1.08596	0.18819
F	-3.26359	1.88273	2.35259
F	-2.35775	3.75870	1.71496
F	-1.11778	2.00805	2.07404

9.25. $[FAl(OR^F)_3]^-$

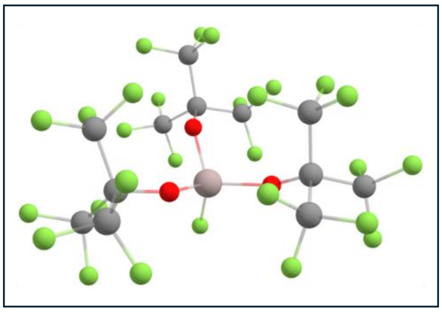

Total enthalpy: -3721.15451682 Eh

Total entropy: 0.11335414 Eh

Final Gibbs free energy: -3721.26787096 Eh

Cartesian coordinates in Ångström:

$[F\text{-}Al(OR^F)_3]^-$

atom	x-value	y-value	z-value
Al	-0.04553	-0.00640	0.00422
O	-1.58010	0.84380	-0.14211
O	0.22170	-0.52449	1.65993
O	-0.19436	-1.47054	-0.95401
C	-2.13949	2.05306	0.04554
C	-0.01638	-1.48155	2.57073
C	-0.50999	-1.93638	-2.17500
F	1.26242	0.93317	-0.44569
C	-0.07193	-3.44637	-2.24023
C	0.23900	-1.13780	-3.30226
C	-2.06259	-1.84623	-2.43336
F	1.14072	-3.61002	-1.69588
F	-0.01937	-3.92194	-3.50647
F	-0.93354	-4.22172	-1.55197
F	-2.74135	-2.17207	-1.32694
F	-2.46945	-2.67429	-3.42638
F	-2.42187	-0.59677	-2.78071
F	1.54765	-1.45864	-3.32394
F	0.14946	0.18000	-3.07485
F	-0.26059	-1.37790	-4.53650
C	0.67584	-2.84159	2.16834
C	-1.55679	-1.73438	2.75389
C	0.58908	-0.99177	3.93758
F	1.93490	-1.08734	3.91740
F	0.14585	-1.70619	4.99761
F	0.28213	0.29320	4.15821
F	-2.15890	-1.81401	1.55983
F	-2.13215	-0.72204	3.43160
F	-1.82030	-2.87682	3.42993
F	-0.07029	-3.52451	1.28179
F	0.87872	-3.65484	3.23354
F	1.86729	-2.60642	1.60307
C	-3.70037	1.86462	0.06918
C	-1.75857	3.02848	-1.13036
C	-1.68454	2.69634	1.40727
F	-1.80537	2.38674	-2.30496
F	-0.50815	3.49965	-0.97317

F	-2.58976	4.09570	-1.21163
F	-4.17639	1.66020	-1.17552
F	-4.34578	2.94039	0.57857
F	-4.03611	0.80021	0.80820
F	-2.30122	2.10386	2.44732
F	-1.94881	4.02076	1.47707
F	-0.36357	2.53265	1.57520

9.26. $[Al(OR^F)_4]^-$

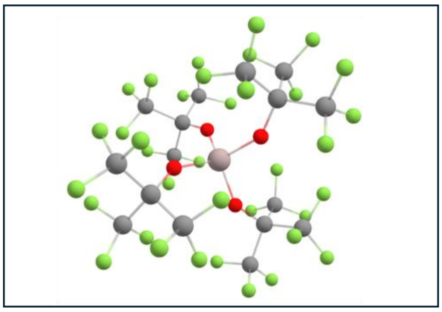

Total enthalpy: -4747.47333644 Eh

Total entropy: 0.13657055 Eh

Final Gibbs free energy: -4747.60990699 Eh

Cartesian coordinates in Ångström:

$[Al(OR^F)_4]^-$

atom	x-value	y-value	z-value
Al	-0.02784	-0.00248	0.01143
O	-1.53903	0.85747	-0.17139
O	0.30956	-0.51998	1.64556
O	-0.13442	-1.44715	-0.96667
C	-2.16051	2.04231	-0.01391
C	0.08484	-1.45724	2.58548
C	-0.52152	-1.94813	-2.15493
O	1.25644	1.08123	-0.46289
C	2.57258	1.35656	-0.40509
C	-0.06970	-3.45441	-2.21645
C	0.14804	-1.16859	-3.34378
C	-2.08717	-1.88131	-2.31643
F	1.16901	-3.59564	-1.72925
F	-0.07535	-3.94454	-3.47748
F	-0.88815	-4.22932	-1.47757
F	-2.68710	-2.16905	-1.15480
F	-2.54739	-2.75017	-3.24733
F	-2.48106	-0.64949	-2.68785
F	1.44967	-1.49489	-3.45439
F	0.07915	0.15322	-3.12578
F	-0.44053	-1.42245	-4.53346
C	0.56107	-2.88100	2.10652
C	-1.44125	-1.53174	2.95370
C	0.90118	-1.05213	3.86746
F	2.21217	-1.31285	3.69586
F	0.49414	-1.71681	4.97322
F	0.77940	0.25849	4.11135

F	-2.18716	-1.50064	1.83944
F	-1.80153	-0.47967	3.71359
F	-1.75938	-2.65359	3.63687
F	-0.35572	-3.44738	1.30082
F	0.77003	-3.73001	3.14013
F	1.70549	-2.78351	1.41962
C	-3.68518	1.83552	-0.34561
C	-1.57320	3.13111	-0.98901
C	-2.02756	2.55951	1.46358
F	-1.30920	2.59125	-2.18489
F	-0.42696	3.64258	-0.50565
F	-2.42864	4.16347	-1.17996
F	-3.87923	1.76833	-1.67748
F	-4.45798	2.84061	0.12694
F	-4.13329	0.69163	0.18564
F	-2.83600	1.86420	2.28600
F	-2.34165	3.86715	1.59184
F	-0.76899	2.39368	1.89815
C	3.00338	1.97143	-1.78828
C	3.44029	0.07131	-0.13019
C	2.85033	2.40582	0.73327
F	3.12240	1.00280	-2.71842
F	4.18754	2.62212	-1.72198
F	2.08533	2.84052	-2.22787
F	2.94390	-0.97344	-0.80837
F	3.42757	-0.24929	1.17633
F	4.73185	0.22915	-0.49731
F	2.43369	3.63326	0.36311
F	4.16471	2.49888	1.04072
F	2.18892	2.07329	1.84784

9.27. HOR^F

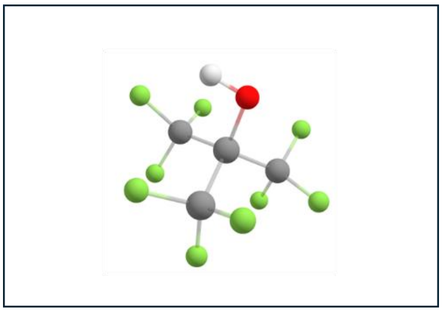

Total enthalpy: -1126.75365708 Eh

Total entropy: 0.05147337 Eh

Final Gibbs free energy: -1126.80513044 Eh

Cartesian coordinates in Ångström:

HOR^F

atom	x-value	y-value	z-value
O	-0.16012	-1.43057	-0.99518
C	-0.55056	-1.96558	-2.22176

C	-0.08885	-3.45761	-2.20332
C	0.15321	-1.19504	-3.39195
C	-2.10818	-1.88096	-2.37834
F	1.15264	-3.56284	-1.72104
F	-0.11174	-3.97335	-3.44501
F	-0.89821	-4.19530	-1.42675
F	-2.71371	-2.23173	-1.23849
F	-2.56753	-2.66156	-3.36533
F	-2.46375	-0.60294	-2.64543
F	1.44646	-1.53331	-3.48091
F	0.10039	0.13293	-3.14044
F	-0.42948	-1.41715	-4.57715
H	-0.37863	-0.48839	-0.97774

9.28. $R^F(OH)-Al(OR^F)_3$

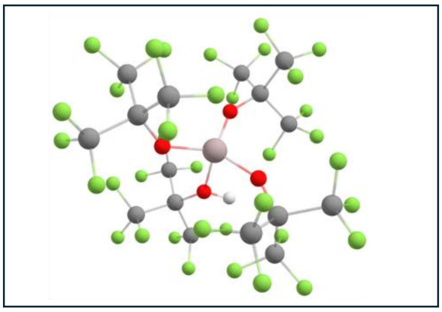

Total enthalpy: -4747.88242664 Eh

Total entropy: 0.13509387 Eh

Final Gibbs free energy: -4748.01752051 Eh

Cartesian coordinates in Ångström:

$R^F(OH)-Al(OR^F)_3$

atom	x-value	y-value	z-value
Al	-0.03418	0.23230	0.19012
O	-1.58847	0.78945	-0.28919
O	0.35401	-0.58792	1.64099
O	0.01605	-1.41330	-0.93247
C	-2.26819	1.96347	-0.11307
C	0.05837	-1.48080	2.62052
C	-0.44700	-1.90927	-2.19282
O	1.29257	1.10134	-0.44709
C	2.61589	1.39012	-0.35292
C	-0.02571	-3.42015	-2.26996
C	0.22373	-1.08583	-3.34288
C	-2.01319	-1.78976	-2.25059
F	1.22857	-3.54741	-1.77224
F	-0.03032	-3.86371	-3.52350
F	-0.83029	-4.18091	-1.52463
F	-2.53505	-2.03355	-1.04780
F	-2.48889	-2.69066	-3.12650
F	-2.39466	-0.57872	-2.65647
F	1.52023	-1.41908	-3.44435
F	0.14542	0.21835	-3.07172
F	-0.37929	-1.32947	-4.51162

C	0.20037	-2.94329	2.06851
C	-1.40441	-1.27000	3.14769
C	1.07787	-1.26816	3.79887
F	2.27681	-1.78791	3.47737
F	0.65727	-1.85769	4.93170
F	1.24482	0.03635	4.03469
F	-2.23607	-1.07522	2.10709
F	-1.47082	-0.18336	3.93145
F	-1.84674	-2.32677	3.84715
F	-0.87443	-3.27616	1.32500
F	0.34842	-3.85757	3.02895
F	1.27691	-3.02013	1.24603
C	-3.79884	1.68975	-0.31068
C	-1.77881	3.02801	-1.15939
C	-2.02053	2.50888	1.33721
F	-1.63733	2.46159	-2.36470
F	-0.57652	3.51207	-0.79238
F	-2.63243	4.05806	-1.27119
F	-4.09699	1.57018	-1.61582
F	-4.54118	2.69009	0.19806
F	-4.15027	0.54943	0.29573
F	-2.77730	1.87499	2.23944
F	-2.22235	3.82145	1.45739
F	-0.71662	2.25196	1.66451
C	3.07240	1.97222	-1.73965

9.29. Xenon

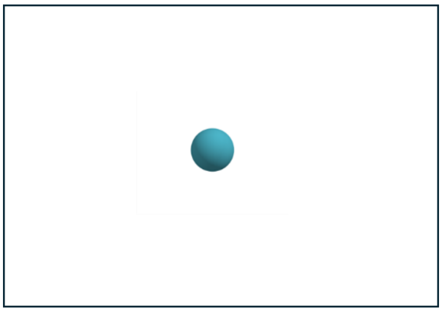

Final single point energy: -329.487151243127 Eh

Total enthalpy: -329.484792 Eh

Total entropy: 0.000064628 Eh

Final Gibbs free energy: -329.5040513 Eh

Cartesian coordinates in Ångström:

Xenon

atom	x-value	y-value	z-value
Xe	-2.58363	0.43881	0.30853

9.30. [$C_6H_3F_2Xe]^+$

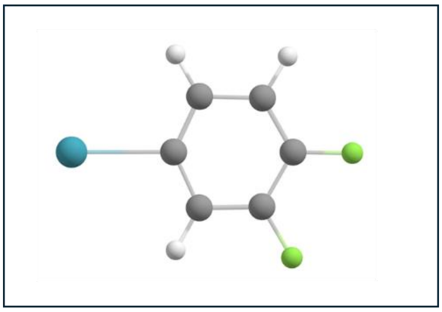

Total enthalpy: -759.09337025 Eh

Total entropy: 0.04353782 Eh

Final Gibbs free energy: -759.13690807 Eh

Cartesian coordinates in Ångström:

[$C_6H_3F_2Xe]^+$

atom	x-value	y-value	z-value
H	-0.96555	-0.83173	1.97649
C	-1.45960	-0.38434	1.12369
C	-2.84750	-0.24256	1.11343
C	-3.49889	0.33391	0.01701
F	-4.81458	0.44780	0.05009
C	-2.78452	0.77864	-1.08281
H	-3.31425	1.22297	-1.91966
C	-1.38976	0.66293	-1.12848
H	-0.82594	1.00868	-1.98397
C	-0.85363	0.08704	-0.01115
F	-3.54669	-0.65664	2.15224
Xe	1.40937	-0.12726	-0.02894

9.31. $C_6H_3F_2Xe$-F-Al($OR^F)_3$

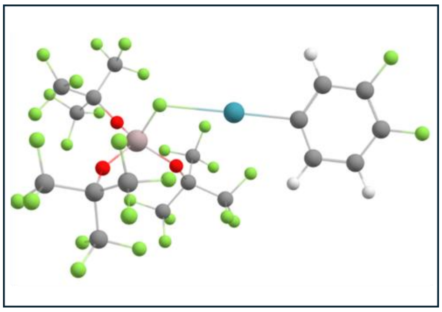

Total enthalpy: -4480.37074328 Eh

Total entropy: 0.13263091 Eh

Final Gibbs free energy: -4480.50337419 Eh

Cartesian coordinates in Ångström:

$C_6H_3F_2Xe$-F-Al($OR^F)_3$

atom	x-value	y-value	z-value
H	-3.38235	1.19002	3.28132

C	-3.18690	0.84725	2.27131
C	-4.23705	0.49218	1.42482
C	-3.89323	0.06520	0.16023
C	-2.60877	-0.03956	-0.33619
H	-2.36750	-0.37899	-1.33579
C	-1.58997	0.32226	0.53430
F	-0.32260	0.25106	0.13389
C	-1.87920	0.76214	1.82617
F	-0.87097	1.10051	2.62800
Xe	-5.52819	-0.48653	-1.16328
H	-5.26400	0.54508	1.76318
F	-7.43433	-1.06207	-2.70340
F	-8.72424	1.84674	-3.26071
F	-6.32676	-2.00470	1.75045
F	-7.36559	-3.71723	-4.66057
F	-8.37691	1.26142	-1.20127
F	-6.78527	-3.87488	-0.19988
F	-9.29895	-1.83358	-4.84487
C	-9.31097	1.75821	-2.05527
Al	-8.89982	-1.63375	-1.98052
F	-7.61052	-5.16694	-3.07050
C	-7.66321	-1.81103	1.94206
O	-9.32181	-3.20798	-2.55117
F	-7.54428	-4.55716	1.72474
F	-9.61080	2.99796	-1.64112
F	-10.87533	0.70419	-4.47011
C	-8.22204	-4.61768	-4.13033
F	-7.85489	-0.47973	1.91532
F	-7.96530	-2.25393	3.16706
C	-7.89103	-3.95700	0.56897
O	-10.17825	-0.49263	-2.21031
C	-10.04298	-2.96100	-4.84415
C	-9.55077	-3.90540	-3.69011
C	-10.55249	0.80098	-2.09998
O	-8.35412	-1.75180	-0.31121
C	-8.48044	-2.51304	0.80096
F	-9.94008	-3.52219	-6.05993
F	-8.42635	-5.58381	-5.04271
C	-11.43839	1.16814	-3.34740
F	-11.59889	2.50051	-3.48165
F	-8.76841	-4.74451	-0.06171
F	-11.32034	-2.59653	-4.65317
F	-10.58283	1.03363	0.29019
C	-11.39705	0.99546	-0.78652
F	-12.65508	0.60979	-3.23617
C	-10.65420	-4.98294	-3.39510
C	-9.98268	-2.63077	1.24271
F	-10.14705	-5.99282	-2.66384
F	-10.17628	-3.62739	2.12476
F	-11.65817	-4.43973	-2.69637
F	-10.39863	-1.48526	1.81152
F	-12.11292	2.13560	-0.80017
F	-12.24008	-0.02783	-0.61924
F	-11.16251	-5.50550	-4.52847
F	-10.75139	-2.85705	0.16744

9.32. $[C_6F_3H_2Xe]^+$

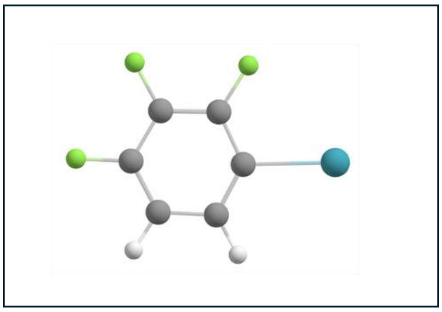

Total enthalpy: -858.32544837 Eh

Total entropy: 0.04543354 Eh

Final Gibbs free energy: -858.37088191 Eh

Cartesian coordinates in Ångström:

$[C_6F_3H_2Xe]^+$

atom	x-value	y-value	z-value
F	-0.83267	-0.90905	2.10793
C	-1.47582	-0.36653	1.09035
C	-2.86533	-0.23486	1.10302
C	-3.50511	0.34207	0.00185
F	-4.81989	0.45348	0.03879
C	-2.79546	0.78905	-1.10317
H	-3.33000	1.23138	-1.93709
C	-1.40527	0.67356	-1.14734
H	-0.84478	1.02050	-2.00576
C	-0.83038	0.10038	-0.04214
F	-3.54605	-0.65447	2.14860
Xe	1.35923	-0.14606	0.02289

9.33. $C_6H_2F_3Xe\text{-}F\text{-}Al(OR^F)_3$

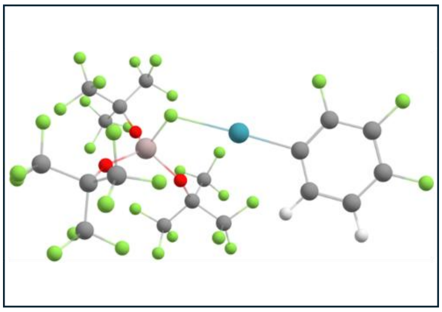

Total enthalpy: -4579.60812591 Eh

Total entropy: 0.13442499 Eh

Final Gibbs free energy: -4579.74255090 Eh

Cartesian coordinates in Ångström:

$C_6H_2F_3Xe\text{-}F\text{-}Al(OR^F)_3$

atom	x-value	y-value	z-value
H	-3.40252	1.18861	3.29532

C	-3.21443	0.84961	2.28335
C	-4.26493	0.49264	1.44252
C	-3.95255	0.06607	0.16719
C	-2.65742	-0.02167	-0.31444
F	-2.39493	-0.43382	-1.55028
C	-1.61342	0.33725	0.53355
F	-0.35921	0.26631	0.10731
C	-1.90901	0.76997	1.82517
F	-0.89488	1.10890	2.61762
Xe	-5.53707	-0.49544	-1.17278
H	-5.29021	0.54002	1.78814
F	-7.42026	-1.06767	-2.68893
F	-8.70705	1.84893	-3.25399
F	-6.32268	-2.00914	1.75492
F	-7.35509	-3.72933	-4.65097
F	-8.36385	1.26027	-1.19489
F	-6.78369	-3.87847	-0.19536
F	-9.27568	-1.83282	-4.83818
C	-9.29633	1.75984	-2.05002
Al	-8.89544	-1.63761	-1.97255
F	-7.61389	-5.17961	-3.06348
C	-7.65897	-1.81627	1.94891
O	-9.31211	-3.20975	-2.54789
F	-7.54307	-4.56229	1.72860
F	-9.59527	2.99882	-1.63431
F	-10.85776	0.71115	-4.46870
C	-8.21828	-4.62552	-4.12474
F	-7.85079	-0.48470	1.92453
F	-7.95963	-2.26122	3.17313
C	-7.88922	-3.96072	0.57373
O	-10.16388	-0.48962	-2.21130
C	-10.02636	-2.95622	-4.84233
C	-9.54374	-3.90521	-3.68806
C	-10.53849	0.80388	-2.09818
O	-8.34887	-1.75374	-0.30415
C	-8.47690	-2.51631	0.80759
F	-9.92223	-3.51595	-6.05821
F	-8.42619	-5.58899	-5.03871
C	-11.42239	1.17363	-3.34625
F	-11.58153	2.50622	-3.47797
F	-8.76785	-4.74601	-0.05780
F	-11.30214	-2.58424	-4.65528
F	-10.57147	1.03072	0.29233
C	-11.38457	0.99552	-0.78543
F	-12.63939	0.61576	-3.23747
C	-10.65527	-4.97492	-3.39620
C	-9.97951	-2.63199	1.24798
F	-10.15754	-5.98766	-2.66270
F	-10.17534	-3.62975	2.12773
F	-11.65783	-4.42426	-2.70099
F	-10.39395	-1.48680	1.81829
F	-12.09966	2.13584	-0.79755
F	-12.22791	-0.02804	-0.62170
F	-11.16308	-5.49407	-4.53111
F	-10.74744	-2.85479	0.17114

9.34. $[C_6H_5Xe]^+$

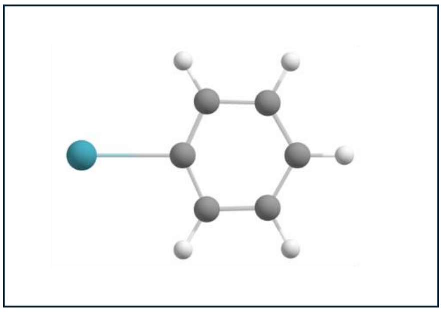

Total enthalpy: -560.61284856 Eh

Total entropy: 0.03956643 Eh

Final Gibbs free energy: -560.65241499 Eh

Cartesian coordinates in Ångström:

$[C_6H_5Xe]^+$

atom	x-value	y-value	z-value
H	-0.94471	-0.82754	1.96324
C	-1.46072	-0.38355	1.12296
C	-2.85765	-0.23842	1.10614
C	-3.50445	0.33437	0.01793
C	-2.78611	0.77981	-1.08479
H	-3.28393	1.22851	-1.93869
C	-1.38712	0.66407	-1.13163
H	-0.81578	1.00671	-1.98348
C	-0.87000	0.08865	-0.01028
Xe	1.44940	-0.12781	-0.03539
H	-3.41148	-0.58893	1.97143
H	-4.58387	0.43515	0.02946

9.35. $C_6H_5Xe\text{-}F\text{-}Al(OR^F)_3$

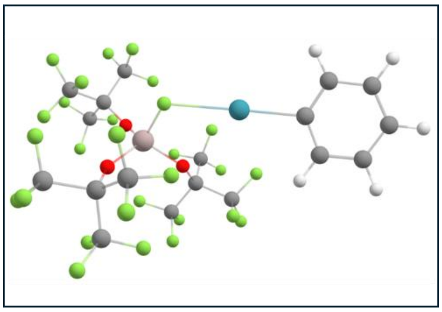

Total enthalpy: -4281.88229595 Eh

Total entropy: 0.12958946 Eh

Final Gibbs free energy: -4282.01188540 Eh

Cartesian coordinates in Ångström:

$C_6H_5Xe\text{-}F\text{-}Al(OR^F)_3$

atom	x-value	y-value	z-value
H	-3.39247	1.19274	3.28655

C	-3.16055	0.85297	2.28289
C	-4.21160	0.50160	1.43391
C	-3.85137	0.07607	0.17485
C	-2.56871	-0.03343	-0.31468
H	-2.35178	-0.37738	-1.31812
C	-1.54565	0.32665	0.56443
H	-0.51718	0.25690	0.22676
C	-1.84135	0.76575	1.85071
H	-1.03624	1.04122	2.52267
Xe	-5.49591	-0.47792	-1.16519
H	-5.24277	0.55553	1.76012
F	-7.43391	-1.06135	-2.72170
F	-8.72870	1.84613	-3.27660
F	-6.31765	-2.00317	1.74379
F	-7.37826	-3.71829	-4.67639
F	-8.36631	1.26465	-1.21859
F	-6.77683	-3.87629	-0.20634
F	-9.31522	-1.83673	-4.85319
C	-9.30601	1.75849	-2.06651
Al	-8.88857	-1.63063	-1.98867
F	-7.61123	-5.16428	-3.08114
C	-7.65341	-1.80969	1.93466
O	-9.32178	-3.20559	-2.55450
F	-7.53476	-4.55642	1.71931
F	-9.60458	2.99946	-1.65268
F	-10.88713	0.69875	-4.46863
C	-8.23037	-4.61749	-4.13790
F	-7.84655	-0.47893	1.90850
F	-7.95586	-2.25296	3.16041
C	-7.88188	-3.95658	0.56288
O	-10.17423	-0.49306	-2.21054
C	-10.05761	-2.96439	-4.84350
C	-9.55677	-3.90522	-3.69002
C	-10.54743	0.80040	-2.10073
O	-8.34535	-1.75220	-0.31836
C	-8.47116	-2.51229	0.79378
F	-9.96289	-3.52939	-6.05880
F	-8.43998	-5.58657	-5.04678
C	-11.44206	1.16477	-3.34273
F	-11.60456	2.49696	-3.47937
F	-8.76004	-4.74514	-0.06621
F	-11.33445	-2.60111	-4.64510
F	-10.56276	1.04158	0.28921
C	-11.38343	0.99862	-0.78220
F	-12.65815	0.60640	-3.22212
C	-10.65703	-4.98370	-3.38617
C	-9.97293	-2.63063	1.23733
F	-10.14401	-5.99229	-2.65687
F	-10.16528	-3.62676	2.12102
F	-11.65729	-4.44122	-2.68167
F	-10.38916	-1.48500	1.80616
F	-12.10107	2.13823	-0.79473
F	-12.22478	-0.02466	-0.60577
F	-11.17259	-5.50924	-4.51531
F	-10.74356	-2.85850	0.16404

9.36. $[XeF]^+$

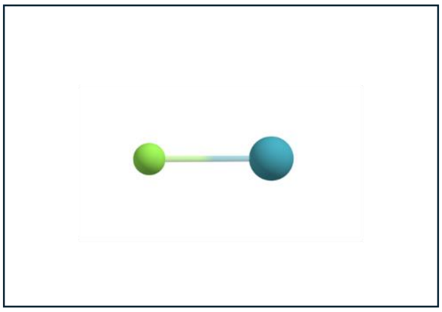

Total enthalpy: -428.82486979 Eh

Total entropy: 0.02681538 Eh

Final Gibbs free energy: -428.85168517 Eh

Cartesian coordinates in Ångström:

$[XeF]^+$

atom	x-value	y-value	z-value
Xe	-3.00693	-1.32785	0.44676
F	-1.92465	-2.89354	0.46060

9.37. $C_6H_3F_2XeF$

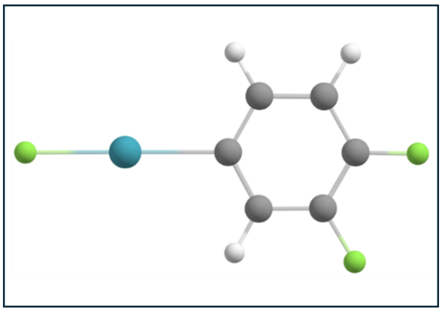

Total enthalpy: -859.17820763 Eh

Total entropy: 0.04650292 Eh

Final Gibbs free energy: -859.22471055 Eh

Cartesian coordinates in Ångström:

$C_6H_3F_2XeF$

atom	x-value	y-value	z-value
C	-3.86638	1.94285	1.39152
C	-4.64119	2.15370	0.25385
C	-2.67859	1.23190	1.31526
C	-4.24005	1.65775	-0.97398
C	-2.29359	0.74260	0.07611
C	-3.04797	0.93983	-1.06854
Xe	-0.39973	-0.41051	-0.06081
H	-4.86361	1.83688	-1.84314
H	-2.73058	0.54840	-2.02873
F	-5.78377	2.84427	0.36448
F	-4.28315	2.43350	2.56580

H	-2.09499	1.08139	2.21725
F	1.42850	-1.53253	-0.18399

9.38. $C_6H_4F_2XeF$

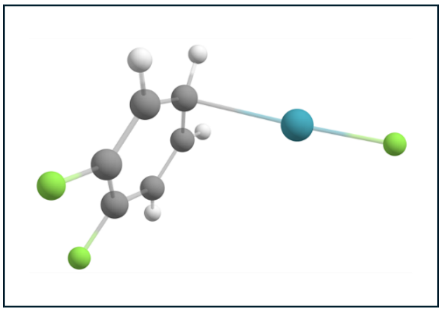

Total enthalpy: -859.47911347 Eh

Total entropy: 0.04746580 Eh

Final Gibbs free energy: -859.52657926 Eh

Cartesian coordinates in Ångström:

$C_6H_3F_2XeF$

atom	x-value	y-value	z-value
C	-3.82213	1.95425	1.42178
C	-4.62319	2.12325	0.26514
C	-2.62683	1.29299	1.33682
C	-4.24135	1.61965	-0.97937
C	-2.22798	0.74671	0.08157
C	-3.04046	0.95452	-1.07875
Xe	-2.81595	-1.74601	0.37107
H	-4.88616	1.78633	-1.83542
H	-2.69916	0.58383	-2.03994
F	-5.75227	2.76974	0.38996
F	-4.25371	2.46617	2.55782
H	-1.99985	1.19552	2.21747
H	-1.17592	0.50617	-0.05944
F	-3.20783	-3.72462	0.66993

9.39. $C_6H_2F_3XeF$

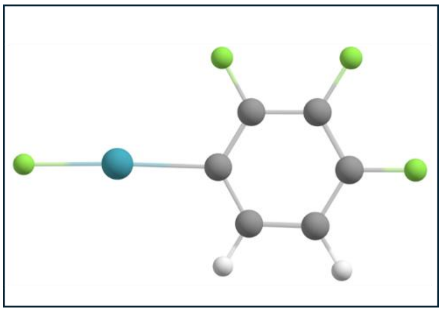

Total enthalpy: -958.42025675 Eh

Total entropy: 0.04848397 Eh

Final Gibbs free energy: -958.46874072 Eh

Cartesian coordinates in Ångström:

$C_6H_2F_3XeF$

atom	x-value	y-value	z-value
C	-3.91799	1.95406	1.38246
C	-4.68930	2.10279	0.23348
C	-2.67976	1.32682	1.28350
C	-4.24786	1.64040	-0.99510
C	-2.23222	0.86210	0.06150
C	-3.00624	1.01426	-1.07842
Xe	-0.24944	-0.12411	-0.05386
H	-4.87639	1.77503	-1.86779
H	-2.65268	0.64960	-2.03680
F	-5.87800	2.70764	0.34388
F	-4.35705	2.40484	2.55791
F	-1.94010	1.18778	2.39193
F	1.64992	-1.07618	-0.24662

9.40. $C_6H_3F_3XeF$

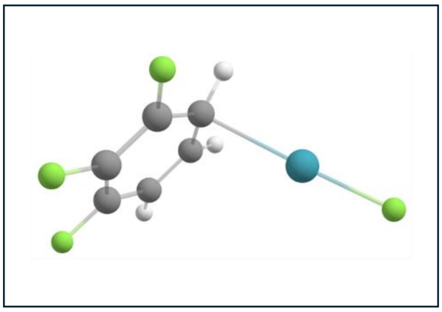

Total enthalpy: -958.71885347 Eh

Total entropy: 0.04923052 Eh

Final Gibbs free energy: -958.76808399 Eh

Cartesian coordinates in Ångström:

$C_6H_3F_3XeF$

atom	x-value	y-value	z-value
C	-3.86049	1.94444	1.41326
C	-4.63094	2.13599	0.25254
C	-2.66487	1.26572	1.29148
C	-4.24261	1.65094	-1.00744
C	-2.25265	0.71246	0.04093
C	-3.05753	0.97361	-1.11754
Xe	-2.79542	-1.74138	0.42369
H	-4.88041	1.85114	-1.86126
H	-2.71408	0.61736	-2.08293
F	-5.75503	2.79121	0.37429
F	-4.26678	2.42381	2.56826
F	-1.89575	1.09772	2.34452
H	-1.18777	0.51809	-0.08020

F	-3.16846	-3.71260	0.75905

9.41. C_6H_5XeF

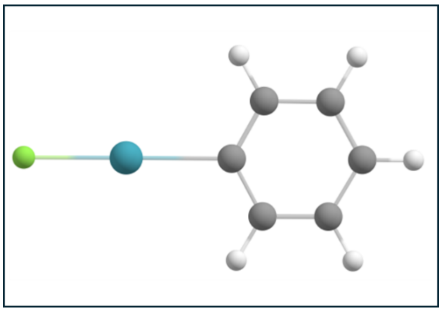

Total enthalpy: -660.68468455 Eh

Total entropy: 0.04248261 Eh

Final Gibbs free energy: -660.72716716 Eh

Cartesian coordinates in Ångström:

Name			
atom	x-value	y-value	z-value
C	-3.97842	1.99250	1.43972
C	-4.71843	2.14071	0.27044
C	-2.74081	1.34990	1.41107
C	-4.22743	1.64906	-0.93527
C	-2.27568	0.86956	0.19829
C	-2.99160	1.00418	-0.97968
Xe	-0.31008	-0.17014	0.13855
H	-4.80336	1.76402	-1.84801
H	-2.60780	0.61957	-1.91911
H	-4.36011	2.37539	2.38085
H	-2.16309	1.23277	2.32224
F	1.59403	-1.19093	0.07483
H	-5.68074	2.64064	0.29901

9.42. C_6H_6XeF

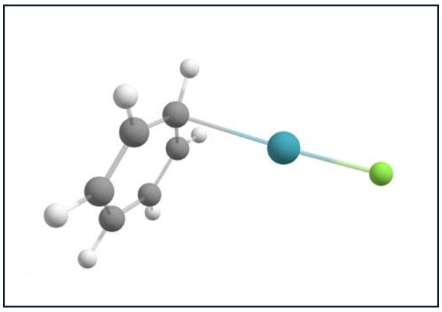

Total enthalpy: -660.98991841 Eh

Total entropy: 0.04361520 Eh

Final Gibbs free energy: -661.03353360 Eh

Cartesian coordinates in Ångström:

C_6H_6XeF

atom	x-value	y-value	z-value
C	-3.84631	1.92087	1.43874
C	-4.63727	2.10809	0.29604
C	-2.63628	1.27029	1.33135
C	-4.23372	1.63892	-0.96229
C	-2.21307	0.76294	0.06290
C	-3.02727	0.98558	-1.09150
Xe	-2.83221	-1.68819	0.45268
H	-4.86016	1.80974	-1.83051
H	-2.68095	0.63985	-2.06057
H	-4.17831	2.30576	2.39638
H	-1.99408	1.13967	2.19687
H	-1.16086	0.51987	-0.07846
F	-3.24547	-3.65582	0.75681
H	-5.58435	2.63110	0.38756

9.43. C_6H_4FXeF

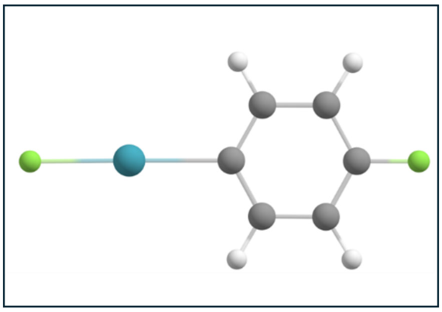

Total enthalpy: -759.93503199 Eh

Total entropy: 0.04440055 Eh

Final Gibbs free energy: -759.97943255 Eh

Cartesian coordinates in Ångström:

C_6H_4FXeF

atom	x-value	y-value	z-value
C	-3.86812	1.95654	1.42694
C	-4.63091	2.17813	0.29116
C	-2.69595	1.21295	1.30607
C	-4.27480	1.69093	-0.95665
C	-2.33422	0.72281	0.06088
C	-3.10146	0.94798	-1.07134
Xe	-0.46560	-0.46391	-0.11449
H	-4.90535	1.89149	-1.81548
H	-2.80529	0.55763	-2.03950
F	-5.76543	2.89665	0.40391
H	-4.18863	2.35861	2.38141
H	-2.08443	1.02772	2.18315
F	1.34948	-1.61637	-0.26397

9.44. C_6H_5FXeF

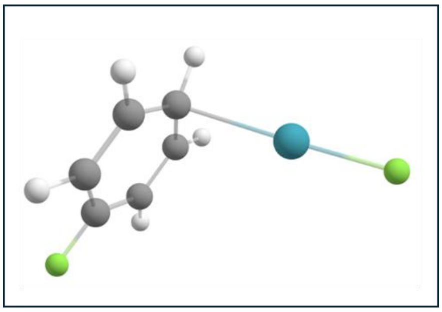

Total enthalpy: -760.24192799 Eh

Total entropy: 0.04545719 Eh

Final Gibbs free energy: -760.28738517 Eh

Cartesian coordinates in Ångström:

C_6H_5FXeF

atom	x-value	y-value	z-value
C	-3.84731	1.93759	1.45069
C	-4.61578	2.12109	0.29107
C	-2.65520	1.26877	1.33535
C	-4.23898	1.65254	-0.97679
C	-2.24056	0.73972	0.07010
C	-3.04644	0.98394	-1.08894
Xe	-2.80076	-1.71894	0.45100
H	-4.87841	1.84870	-1.83023
H	-2.70413	0.63306	-2.05755
F	-5.75207	2.76979	0.39816
H	-4.19720	2.34432	2.39297
H	-2.01683	1.13315	2.20289
H	-1.18540	0.51382	-0.07380
F	-3.19371	-3.69905	0.75374

9.45. $[C_6H_4FXe]^+$

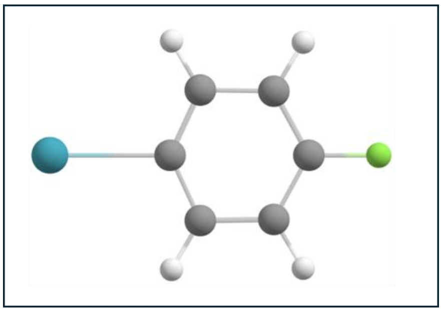

Total enthalpy: -659.85610450 Eh

Total entropy: 0.04141873 Eh

Final Gibbs free energy: -659.89752323 Eh

Cartesian coordinates in Ångström:

$[C_6H_4FXe]^+$

atom	x-value	y-value	z-value
H	-0.95864	-0.83445	1.98209
C	-1.47621	-0.39047	1.14212
C	-2.86604	-0.25255	1.13966
C	-3.49347	0.32321	0.04039
F	-4.81446	0.44566	0.05633
C	-2.79745	0.77639	-1.07480
H	-3.32641	1.22000	-1.91161
C	-1.40645	0.65638	-1.11115
H	-0.83652	0.99958	-1.96436
C	-0.86986	0.07998	0.00853
H	-3.44717	-0.59306	1.99007
Xe	1.40113	-0.13121	-0.01933

9.46. $C_6H_4FXe\text{-}F\text{-}Al(OR^F)_3$

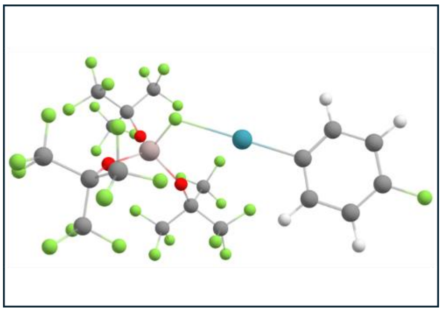

Total enthalpy: -4381.13028095 Eh

Total entropy: 0.13102446 Eh

Final Gibbs free energy: -4381.26130541 Eh

Cartesian coordinates in Ångström:

$C_6H_4FXe\text{-}F\text{-}Al(OR^F)_3$			
atom	x-value	y-value	z-value
H	-3.37376	1.18982	3.28223
C	-3.17651	0.84549	2.27360
C	-4.22103	0.49472	1.42202
C	-3.87399	0.06525	0.15732
C	-2.58799	-0.04617	-0.33054
H	-2.37311	-0.39125	-1.33423
C	-1.55527	0.30813	0.53399
H	-0.51962	0.24558	0.22059
C	-1.87162	0.74539	1.81252
F	-0.87237	1.08516	2.63617
Xe	-5.51380	-0.47981	-1.16841
H	-5.25035	0.55273	1.75404
F	-7.43597	-1.05788	-2.71400
F	-8.72993	1.84838	-3.26690
F	-6.31926	-1.99728	1.74408
F	-7.37040	-3.71436	-4.66916
F	-8.37655	1.26432	-1.20808
F	-6.77785	-3.87032	-0.20451
F	-9.30817	-1.83465	-4.84955
C	-9.31306	1.75925	-2.05972
Al	-8.89514	-1.63026	-1.98535

F	-7.60684	-5.16240	-3.07630
C	-7.65558	-1.80495	1.93656
O	-9.32023	-3.20576	-2.55295
F	-7.53362	-4.55132	1.72173
F	-9.61370	2.99912	-1.64545
F	-10.88237	0.70187	-4.47027
C	-8.22334	-4.61529	-4.13442
F	-7.84917	-0.47408	1.90919
F	-7.95604	-2.24738	3.16247
C	-7.88246	-3.95247	0.56564
O	-10.17873	-0.49273	-2.21088
C	-10.05003	-2.96310	-4.84371
C	-9.55172	-3.90481	-3.68998
C	-10.55367	0.80049	-2.10085
O	-8.34943	-1.74878	-0.31588
C	-8.47335	-2.50894	0.79682
F	-9.95112	-3.52632	-6.05919
F	-8.42929	-5.58323	-5.04492
C	-11.44295	1.16596	-3.34635
F	-11.60538	2.49816	-3.48142
F	-8.75982	-4.74194	-0.06299
F	-11.32745	-2.60078	-4.64840
F	-10.57915	1.03595	0.28927
C	-11.39547	0.99554	-0.78565
F	-12.65900	0.60656	-3.23197
C	-10.65192	-4.98436	-3.38993
C	-9.97479	-2.62856	1.24102
F	-10.14001	-5.99289	-2.65995
F	-10.16571	-3.62456	2.12471
F	-11.65437	-4.44289	-2.68776
F	-10.39175	-1.48311	1.80940
F	-12.11293	2.13494	-0.79886
F	-12.23720	-0.02840	-0.61528
F	-11.16375	-5.50910	-4.52091
F	-10.74510	-2.85732	0.16754

9.47. $[C_6F_5Xe]^+$

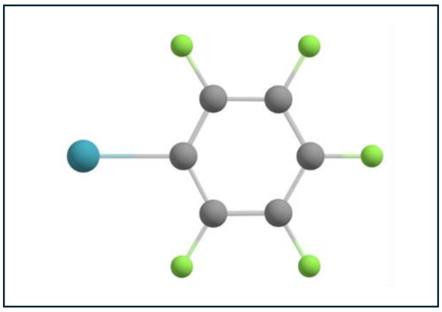

Total enthalpy: -1056.78965958 Eh

Total entropy: 0.04978027 Eh

Final Gibbs free energy: -1056.83943985 Eh

Cartesian coordinates in Ångström:

$[C_6F_5Xe]^+$

atom	x-value	y-value	z-value
F	-0.83853	-0.92711	2.15017
C	-1.47850	-0.38445	1.13001
C	-2.86639	-0.25056	1.13498
C	-3.51601	0.32442	0.04013
F	-4.82402	0.44522	0.05571
C	-2.79799	0.77437	-1.07036
F	-3.42329	1.31748	-2.09481
C	-1.40942	0.65121	-1.09856
F	-0.70510	1.06484	-2.13671
C	-0.80565	0.07557	0.00865
F	-3.55639	-0.66759	2.17680
Xe	1.32976	-0.12397	-0.01807

Vibrational analysis:

$[C_6F_5Xe]^+$							
Mode	freq (cm-1)	eps (L/(mol*cm))	Int (km/mol)	T2 (a.u.)	TX (a.u.)	TY (a.u.)	TZ (a.u.)
6	72.42	0.000046	0.23	0.000199	0.001902	0.012647	0.005988
7	120.10	0.000018	0.09	0.000046	-0.000222	-0.002803	0.006191
8	127.95	0.000000	0.00	0.000000	-0.000005	-0.000610	-0.000280
9	152.95	0.000000	0.00	0.000000	-0.000129	0.000303	0.000092
10	176.37	0.000131	0.66	0.000232	0.000677	0.013718	0.006576
11	182.49	0.000015	0.08	0.000026	0.005029	-0.000810	-0.000240
12	270.25	0.000713	3.60	0.000823	-0.003561	-0.025745	-0.012160
13	275.64	0.000018	0.09	0.000021	-0.000036	0.001315	-0.004334
14	277.28	0.000001	0.01	0.000001	-0.000019	0.001190	0.000282
15	288.02	0.000000	0.00	0.000000	0.000080	0.000069	0.000009
16	306.20	0.000179	0.90	0.000182	0.000828	0.005697	-0.012201
17	346.05	0.000906	4.58	0.000817	0.028448	-0.002762	-0.000146
18	432.90	0.000000	0.00	0.000000	0.000038	0.000218	-0.000516
19	489.65	0.001770	8.94	0.001128	-0.033443	0.003037	0.000348
20	543.48	0.000113	0.57	0.000065	-0.001089	-0.007206	-0.003426
21	588.72	0.000598	3.02	0.000317	-0.017726	0.001650	0.000290
22	637.92	0.000000	0.00	0.000000	0.000013	0.000043	-0.000163
23	658.19	0.000000	0.00	0.000000	-0.000102	0.000242	0.000073
24	753.92	0.022956	116.01	0.009502	-0.097050	0.009036	0.001254
25	758.92	0.000029	0.15	0.000012	0.002683	-0.001126	0.001843
26	1019.27	0.029356	148.35	0.008988	-0.002690	-0.039925	0.085945
27	1105.48	0.013095	66.18	0.003697	0.060532	-0.005668	-0.000606
28	1196.28	0.000140	0.71	0.000037	-0.000242	-0.002536	0.005487
29	1309.08	0.001702	8.60	0.000406	0.020069	-0.001651	-0.000451
30	1334.99	0.007884	39.84	0.001843	0.001169	0.018072	-0.038923
31	1447.85	0.000485	2.45	0.000105	0.010167	-0.001029	-0.000358
32	1534.69	0.056068	283.35	0.011401	0.106341	-0.009147	-0.002964
33	1542.96	0.086282	436.04	0.017451	-0.002195	-0.055783	0.119724
34	1604.12	0.002604	13.16	0.000507	-0.022408	0.002126	0.000107
35	1659.49	0.000963	4.86	0.000181	0.000430	0.005670	-0.012193

9.48. $[C_6F_5Xe(H_2O)]^+$

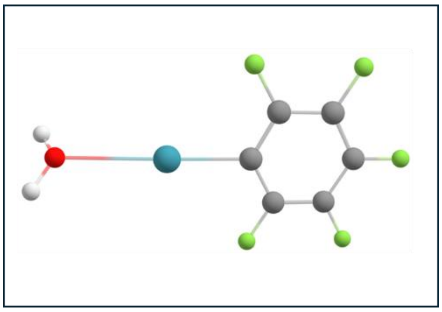

Total enthalpy: -1157.33100193 Eh

Total entropy: 0.05612451 Eh

Final Gibbs free energy: -1157.38712644 Eh

Cartesian coordinates in Ångström:

$[C_6F_5Xe(H_2O)]^+$

atom	x-value	y-value	z-value
F	-0.89490	-0.98000	2.17680
C	-1.49242	-0.41487	1.13895
C	-2.87058	-0.23472	1.13109
C	-3.48488	0.36068	0.03372
F	-4.79830	0.52895	0.03145
C	-2.73712	0.77883	-1.06289
F	-3.33703	1.34359	-2.10274
C	-1.35791	0.60410	-1.06884
F	-0.63639	0.99795	-2.10687
C	-0.77058	0.00964	0.03565
F	-3.59571	-0.63140	2.16875
Xe	1.33124	-0.25274	0.05084
F	4.00666	-0.66715	0.15014
H	4.55954	0.01323	-0.17354

9.49. $[C_6F_5Xe(HF)]^+$

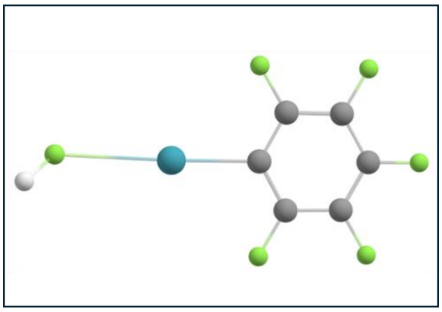

Total enthalpy: -1133.21881605 Eh

Total entropy: 0.05778754 Eh

Final Gibbs free energy: -1133.27660359 Eh

Cartesian coordinates in Ångström:

$[C_6F_5Xe(HF)]^+$

atom	x-value	y-value	z-value
F	-0.89156	-0.90368	2.19790
C	-1.50798	-0.37182	1.15133
C	-2.89192	-0.22257	1.14098
C	-3.51915	0.34037	0.02833
F	-4.82786	0.47989	0.02285
C	-2.77812	0.75722	-1.07864
F	-3.38351	1.28768	-2.12451
C	-1.39365	0.61160	-1.07741
F	-0.67052	1.00087	-2.11809
C	-0.79476	0.04945	0.03957
F	-3.60411	-0.61271	2.18141
Xe	1.32917	-0.17786	0.04778
O	3.98385	-0.51550	-0.04629
H	4.59079	0.22688	-0.15945
H	4.44174	-1.13652	0.53421

9.50. $C_6F_5Xe\text{-}F\text{-}Al(OR^F)_3$

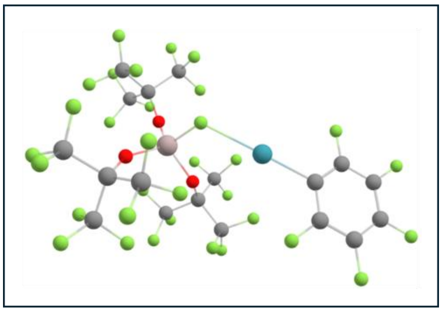

Total enthalpy: -4778.07999955 Eh

Total entropy: 0.13903510 Eh

Final Gibbs free energy: -4778.21903465 Eh

Cartesian coordinates in Ångström:

$C_6F_5Xe\text{-}F\text{-}Al(OR^F)_3$

atom	x-value	y-value	z-value
F	-3.30672	1.90051	3.03954
C	-3.14413	1.07079	2.01828
C	-4.18360	0.85428	1.11920
C	-3.98071	-0.00943	0.05427
C	-2.78082	-0.67647	-0.13121
C	-1.74181	-0.45875	0.76794
F	-0.57912	-1.07700	0.60807
C	-1.92971	0.41158	1.83962
F	-0.94162	0.61719	2.69626
Xe	-5.60956	-0.40069	-1.25706
F	-5.34497	1.47299	1.28634
F	-7.49569	-0.89068	-2.71479
F	-8.92576	1.95594	-3.19075
F	-6.34775	-1.58152	1.75809

F	-7.28765	-3.48383	-4.73312
F	-8.52845	1.31877	-1.15630
F	-6.50554	-3.52250	-0.16221
F	-9.34267	-1.73387	-4.84624
C	-9.49391	1.80299	-1.98222
Al	-8.93313	-1.55459	-1.97584
F	-7.40055	-4.96385	-3.15656
C	-7.70252	-1.59433	1.94791
O	-9.23374	-3.14228	-2.57511
F	-7.22647	-4.32485	1.72889
F	-9.84341	3.01237	-1.52392
F	-11.03319	0.75537	-4.41572
C	-8.06988	-4.44701	-4.19789
F	-8.09933	-0.31779	1.93492
F	-7.93271	-2.10329	3.16371
C	-7.61758	-3.75769	0.57393
O	-10.26161	-0.47896	-2.20603
C	-10.01323	-2.90694	-4.84942
C	-9.43616	-3.83492	-3.72279
C	-10.69313	0.79377	-2.04723
O	-8.35169	-1.64074	-0.32068
C	-8.39561	-2.40606	0.79722
F	-9.90155	-3.44136	-6.07569
F	-8.22680	-5.41376	-5.11792
C	-11.60591	1.16020	-3.27491
F	-11.82332	2.48765	-3.36501
F	-8.36404	-4.63959	-0.09831
F	-11.30631	-2.62895	-4.62338
F	-10.70802	0.93013	0.34843
C	-11.53111	0.90283	-0.72088
F	-12.79645	0.54727	-3.17219
C	-10.45611	-4.99211	-3.42724
C	-9.87723	-2.72571	1.21355
F	-9.86152	-5.97997	-2.73262
F	-9.94910	-3.75916	2.07042
F	-11.47673	-4.53570	-2.69179
F	-10.44955	-1.65983	1.79705
F	-12.29148	2.01250	-0.68561
F	-12.33070	-0.16027	-0.58828
F	-10.95538	-5.52282	-4.56021
F	-10.59437	-3.02779	0.12000
F	-2.61387	-1.51470	-1.14861

Vibrational analysis:

$C_6F_5Xe\text{-}F\text{-}Al(OR^F)_3$

Mode	freq (cm-1)	eps (L/(mol*cm))	Int (km/mol)	T2 (a.u.)	TX (a.u.)	TY (a.u.)	TZ (a.u.)
6	6.15	0.000017	0.08	0.000849	0.006221	0.026974	0.009114
7	9.96	0.000008	0.04	0.000248	0.013434	0.000410	0.008182
8	10.85	0.000000	0.00	0.000008	-0.002314	0.001465	0.000394
9	14.78	0.000009	0.05	0.000196	0.002873	-0.009722	-0.009666
10	16.82	0.000006	0.03	0.000112	0.008045	0.001097	0.006762
11	21.68	0.000015	0.08	0.000222	0.002380	-0.003162	0.014376
12	22.80	0.000052	0.26	0.000712	-0.011980	-0.009088	-0.022040
13	28.00	0.000027	0.14	0.000303	-0.011128	-0.002277	-0.013173
14	30.72	0.000097	0.49	0.000988	-0.022368	0.022026	0.001504
15	39.53	0.000038	0.19	0.000300	0.002075	-0.012529	0.011762
16	39.99	0.000116	0.59	0.000904	0.016503	-0.019870	-0.015393
17	50.12	0.000188	0.95	0.001170	0.002531	0.014695	-0.030781

18	54.50	0.000095	0.48	0.000546	0.015019	0.011974	-0.013297
19	61.88	0.000967	4.89	0.004878	-0.061607	-0.029019	-0.015519
20	64.61	0.000030	0.15	0.000144	-0.010544	-0.005434	-0.001758
21	66.54	0.000204	1.03	0.000955	-0.027281	-0.000459	0.014501
22	72.22	0.000166	0.84	0.000719	-0.026623	-0.002116	0.002352
23	73.80	0.000019	0.10	0.000082	-0.007998	0.003540	-0.002405
24	77.69	0.000089	0.45	0.000357	0.002664	-0.017233	0.007289
25	81.43	0.000032	0.16	0.000123	0.008859	-0.005232	0.004082
26	86.99	0.000026	0.13	0.000092	-0.004140	-0.006694	-0.005453
27	89.49	0.000067	0.34	0.000233	0.011240	0.001791	0.010155
28	91.45	0.000004	0.02	0.000012	-0.000020	-0.003468	-0.000086
29	93.82	0.000051	0.26	0.000171	-0.008130	-0.009731	0.003238
30	95.80	0.000181	0.92	0.000590	-0.011587	-0.014970	-0.015233
31	98.97	0.000737	3.72	0.002324	-0.038264	-0.022127	0.019234
32	99.59	0.000109	0.55	0.000341	0.003143	-0.017577	-0.004745
33	107.61	0.000036	0.18	0.000106	-0.000519	0.002122	0.010040
34	130.19	0.000021	0.11	0.000050	-0.004702	-0.004704	0.002446
35	132.01	0.000117	0.59	0.000278	-0.010882	-0.012151	0.003397
36	154.82	0.000227	1.15	0.000457	0.017748	-0.011896	-0.000639
37	158.52	0.000050	0.25	0.000099	-0.000464	0.009395	-0.003294
38	162.69	0.000064	0.33	0.000124	-0.009546	-0.002728	0.004994
39	165.86	0.000033	0.16	0.000061	-0.001541	0.005577	0.005262
40	167.17	0.000123	0.62	0.000230	-0.003831	-0.000785	0.014636
41	168.33	0.000165	0.84	0.000306	0.000479	0.014582	0.009670
42	173.52	0.000036	0.18	0.000065	0.003471	0.003579	-0.006336
43	182.74	0.000382	1.93	0.000652	0.004914	-0.024969	0.001986
44	186.74	0.000371	1.88	0.000620	-0.001563	0.005823	-0.024163
45	188.70	0.002003	10.12	0.003312	-0.054534	-0.016339	-0.008437
46	201.31	0.000661	3.34	0.001024	-0.009325	0.027816	-0.012788
47	203.00	0.001222	6.18	0.001879	0.019295	-0.035572	-0.015533
48	213.87	0.000012	0.06	0.000018	-0.002539	-0.003129	0.001264
49	248.23	0.000023	0.11	0.000028	0.001226	0.000444	0.005169
50	271.19	0.004771	24.11	0.005490	-0.063520	-0.029296	-0.024426
51	277.26	0.000766	3.87	0.000862	0.027619	0.005812	0.008074
52	278.10	0.001444	7.30	0.001621	0.035901	0.010203	0.015090
53	278.89	0.001337	6.76	0.001497	0.035239	0.008338	0.013610
54	281.56	0.002890	14.61	0.003203	0.051087	0.022767	0.008671
55	282.86	0.000308	1.55	0.000339	0.016321	-0.006773	-0.005201
56	286.11	0.000554	2.80	0.000604	-0.022172	0.010612	0.000226
57	286.99	0.000111	0.56	0.000120	-0.001003	-0.003779	-0.010243
58	287.69	0.000352	1.78	0.000382	-0.012785	-0.005567	-0.013708
59	289.32	0.000313	1.58	0.000338	0.016717	-0.004984	0.005805
60	292.76	0.000337	1.70	0.000359	-0.008421	-0.007443	-0.015246
61	303.56	0.004042	20.43	0.004155	-0.048100	-0.009661	-0.041814
62	309.49	0.000252	1.27	0.000254	0.007803	-0.010401	-0.009199
63	311.76	0.001313	6.64	0.001315	0.008842	-0.034584	-0.006352
64	313.61	0.003858	19.50	0.003839	-0.036121	-0.011512	-0.049010
65	317.26	0.000102	0.51	0.000100	-0.007620	0.001828	-0.006230
66	321.56	0.000613	3.10	0.000595	-0.018195	-0.013502	-0.009009
67	323.35	0.000358	1.81	0.000346	-0.001025	0.010028	-0.015624
68	324.98	0.000948	4.79	0.000910	-0.013015	-0.013436	-0.023665
69	326.93	0.000314	1.59	0.000300	-0.012868	0.009261	-0.006965
70	328.27	0.000277	1.40	0.000264	-0.011779	0.008907	0.006741
71	330.20	0.001802	9.11	0.001703	0.028421	-0.003876	0.029675
72	333.85	0.000624	3.16	0.000584	-0.013510	-0.005624	-0.019222
73	350.55	0.000053	0.27	0.000048	-0.006011	-0.002746	0.001969
74	354.82	0.000006	0.03	0.000005	0.000749	0.002136	0.000066
75	355.75	0.000227	1.15	0.000199	-0.002595	-0.011349	0.007972
76	364.50	0.000283	1.43	0.000242	0.005920	-0.012757	0.006673
77	367.07	0.001837	9.28	0.001562	0.018951	0.028182	0.020205
78	374.31	0.006288	31.78	0.005243	-0.041334	0.035114	-0.047969
79	385.47	0.007751	39.17	0.006275	-0.015779	0.035519	0.069022
80	423.21	0.001576	7.97	0.001162	0.008281	0.010937	0.031209
81	441.30	0.000003	0.01	0.000002	-0.000403	-0.001113	-0.000678
82	458.53	0.011573	58.48	0.007876	0.054253	-0.050164	0.049155

83	463.64	0.008673	43.83	0.005838	-0.018103	-0.049675	-0.055158
84	494.36	0.000012	0.06	0.000008	-0.002178	0.000124	-0.001752
85	523.06	0.001105	5.58	0.000659	-0.014476	-0.017960	-0.011267
86	524.28	0.000428	2.16	0.000254	0.006047	-0.009909	-0.010941
87	525.61	0.000929	4.69	0.000551	0.013255	-0.018771	0.004827
88	526.03	0.000518	2.62	0.000307	-0.007021	-0.000373	0.016062
89	526.69	0.000178	0.90	0.000105	0.005427	-0.007496	0.004437
90	526.92	0.001736	8.77	0.001028	0.030105	0.010902	0.001743
91	527.07	0.000293	1.48	0.000174	0.004044	-0.001670	0.012427
92	531.11	0.000484	2.45	0.000285	-0.008865	0.011364	-0.008766
93	531.46	0.000192	0.97	0.000113	0.000287	-0.002503	-0.010324
94	535.40	0.000700	3.54	0.000408	0.017268	0.004586	0.009414
95	556.72	0.002024	10.23	0.001135	-0.008162	-0.003676	-0.032473
96	560.52	0.001037	5.24	0.000577	0.009695	-0.021949	-0.001156
97	561.91	0.000025	0.13	0.000014	0.000909	-0.002444	0.002689
98	562.81	0.000078	0.39	0.000043	-0.006145	0.000798	0.002216
99	562.92	0.000089	0.45	0.000049	-0.006940	-0.000136	0.000997
100	563.97	0.000108	0.55	0.000060	-0.003085	-0.003276	-0.006289
101	570.02	0.003888	19.65	0.002129	-0.019350	-0.001368	-0.041860
102	574.79	0.005785	29.23	0.003141	0.028144	-0.048439	-0.001504
103	579.38	0.000430	2.17	0.000232	-0.000910	-0.008110	0.012850
104	588.96	0.001025	5.18	0.000543	-0.019430	-0.006294	-0.011230
105	629.82	0.041486	209.65	0.020555	-0.133182	-0.052448	-0.008196
106	646.03	0.000018	0.09	0.000009	-0.002774	-0.000897	0.000172
107	649.02	0.000003	0.02	0.000002	0.000797	-0.000822	0.000573
108	714.80	0.003400	17.18	0.001484	-0.004467	0.031484	0.021753
109	716.78	0.002613	13.20	0.001137	0.015964	0.024526	-0.016766
110	717.05	0.005648	28.54	0.002458	0.037541	-0.029124	-0.014161
111	718.54	0.002185	11.04	0.000949	0.007085	0.028847	0.008165
112	719.13	0.013125	66.33	0.005695	0.062363	-0.003597	0.042346
113	719.68	0.010345	52.28	0.004486	0.044660	0.029603	-0.040185
114	737.07	0.000570	2.88	0.000241	0.009659	0.002153	0.011976
115	745.57	0.001852	9.36	0.000775	-0.022790	-0.014790	-0.006069
116	747.78	0.000219	1.11	0.000091	-0.001231	-0.009213	0.002247
117	760.72	0.000017	0.09	0.000007	-0.002592	-0.000283	0.000517
118	786.39	0.011622	58.73	0.004612	-0.054732	-0.012458	-0.038225
119	799.35	0.001316	6.65	0.000514	-0.015595	-0.006999	0.014884
120	843.11	0.007883	39.84	0.002918	-0.039075	-0.022431	-0.029794
121	868.93	0.005344	27.01	0.001919	-0.019709	0.038840	-0.004714
122	953.83	0.015654	79.11	0.005122	0.070268	-0.013007	0.003839
123	958.22	0.005346	27.02	0.001741	0.034152	0.002813	0.023804
124	962.55	0.015709	79.39	0.005093	0.060559	0.033435	0.017543
125	964.73	0.050661	256.02	0.016387	-0.084713	-0.028598	-0.091615
126	965.10	0.053841	272.09	0.017409	0.119209	-0.047990	-0.029926
127	968.51	0.053364	269.68	0.017195	0.052394	0.100609	-0.065782
128	1009.87	0.032103	162.24	0.009920	0.056893	-0.063461	-0.051538
129	1071.14	0.001628	8.23	0.000474	0.017850	-0.011475	-0.004897
130	1082.18	0.001866	9.43	0.000538	-0.008148	0.013183	-0.017261
131	1088.29	0.000801	4.05	0.000230	-0.001761	-0.001166	0.015012
132	1101.72	0.025206	127.38	0.007140	0.064679	0.015340	0.052163
133	1125.25	0.007795	39.39	0.002162	-0.045915	0.001611	0.007141
134	1130.46	0.003595	18.17	0.000993	0.011178	0.006289	0.028775
135	1137.42	0.004782	24.16	0.001312	-0.000607	-0.024131	0.027004
136	1145.23	0.003885	19.63	0.001059	0.020073	-0.022138	0.012867
137	1146.13	0.004924	24.88	0.001341	0.032115	-0.006870	-0.016192
138	1154.82	0.012182	61.56	0.003292	0.031426	0.002097	0.047957
139	1169.29	0.005433	27.46	0.001450	0.025638	-0.026315	-0.010011
140	1173.66	0.012586	63.60	0.003346	-0.029797	0.030956	-0.038733
141	1178.89	0.004533	22.91	0.001200	-0.012162	-0.032199	0.003905
142	1179.88	0.004007	20.25	0.001060	-0.005753	0.032043	0.000180
143	1187.13	0.009193	46.46	0.002416	-0.043059	-0.023322	0.004301
144	1188.88	0.004353	22.00	0.001143	-0.032822	-0.004171	-0.006925
145	1192.91	0.011196	56.58	0.002929	-0.016691	-0.033602	0.039004
146	1211.00	0.007999	40.42	0.002061	-0.014481	0.042797	0.004460
147	1213.45	0.001758	8.88	0.000452	-0.002418	-0.015291	-0.014573

148	1218.39	0.039299	198.60	0.010066	0.095032	-0.021465	0.023956
149	1225.75	0.174831	883.52	0.044510	0.025904	-0.160104	0.134929
150	1226.53	0.240693	1216.36	0.061239	0.039196	-0.185992	-0.158460
151	1235.48	0.084412	426.59	0.021321	-0.063054	0.012327	-0.131124
152	1238.96	0.077987	394.12	0.019643	0.075532	-0.086209	0.080660
153	1245.11	0.067059	338.89	0.016807	-0.040517	0.062703	0.105989
154	1248.78	0.066981	338.49	0.016738	0.055781	0.008214	0.116444
155	1252.30	0.078141	394.89	0.019472	-0.033866	-0.134249	0.017392
156	1255.55	0.177628	897.66	0.044149	0.199773	0.029077	-0.058262
157	1260.18	0.054860	277.24	0.013585	-0.052067	-0.093964	-0.045223
158	1300.26	0.000011	0.05	0.000003	-0.000463	0.000962	-0.001175
159	1327.07	0.013402	67.73	0.003151	-0.013484	0.027545	-0.047020
160	1335.30	0.029238	147.75	0.006833	0.010436	-0.071582	-0.040001
161	1343.83	0.010085	50.97	0.002342	0.041730	-0.018122	0.016498
162	1347.01	0.003405	17.21	0.000789	-0.016359	0.018164	0.013835
163	1435.55	0.002784	14.07	0.000605	0.021019	0.005251	0.011653
164	1526.59	0.060694	306.72	0.012407	0.096175	0.006756	0.055783
165	1530.48	0.077651	392.42	0.015833	0.062194	-0.080354	-0.074217
166	1628.12	0.002072	10.47	0.000397	-0.014190	-0.002077	-0.013839
167	1652.89	0.000080	0.40	0.000015	0.003827	-0.000292	-0.000532

9.51. $[F(Al(OR^F)_3)_2]^-$

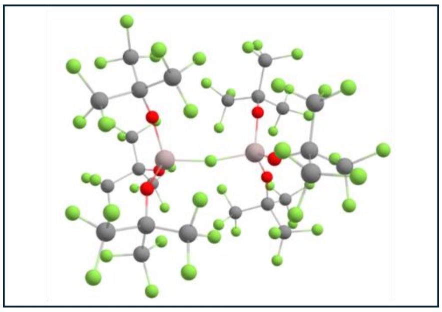

Total enthalpy: -7342.32859654 Eh

Total entropy: 0.19315274 Eh

Final Gibbs free energy: -7342.52174928 Eh

Cartesian coordinates in Ångström:

$[F(Al(OR^F)_3)_2]^-$

atom	x-value	y-value	z-value
Al	0.11980	0.27533	0.04595
O	-1.37673	0.92675	-0.50660
O	0.33888	0.06918	1.75175
O	0.50143	-1.18459	-0.79921
C	-2.24052	1.96397	-0.59868
C	0.03496	-0.77599	2.76775
C	0.27010	-1.86846	-1.94748
F	1.42942	1.45255	-0.30447
C	0.96167	-3.27502	-1.82270
C	0.88060	-1.09407	-3.16673
C	-1.26954	-2.08730	-2.19434
F	2.16971	-3.16229	-1.26512
F	1.11083	-3.87254	-3.02436
F	0.22705	-4.09450	-1.04447

F	-1.89467	-2.32421	-1.03294
F	-1.51088	-3.12809	-3.01946
F	-1.82959	-0.99794	-2.74851
F	2.21688	-1.21169	-3.18032
F	0.58854	0.21569	-3.05982
F	0.41008	-1.53222	-4.35013
C	0.70651	-2.18389	2.54950
C	-1.51931	-0.97130	2.89285
C	0.57960	-0.14506	4.09811
F	1.91709	-0.26722	4.16493
F	0.05447	-0.73924	5.19185
F	0.28121	1.15857	4.15861
F	-2.05735	-1.11961	1.66856
F	-2.09851	0.09978	3.46221
F	-1.84844	-2.05291	3.62570
F	-0.00509	-2.91384	1.66936
F	0.79322	-2.89431	3.69379
F	1.94099	-2.04019	2.05624
C	-3.53515	1.48237	-1.34946
C	-1.57766	3.12943	-1.41097
C	-2.64970	2.47961	0.82902
F	-0.94393	2.63553	-2.48041
F	-0.66290	3.75559	-0.64417
F	-2.46744	4.04704	-1.83401
F	-3.32316	1.39758	-2.67473
F	-4.56626	2.33453	-1.15780
F	-3.91034	0.27296	-0.91604
F	-3.55534	1.65717	1.39134
F	-3.17894	3.71802	0.80031
F	-1.57500	2.50609	1.63074
F	6.61814	-1.36395	-1.31061
F	5.67872	-0.94750	1.27589

10. References

1 I. Krossing, *Chem. Eur. J.*, 2001, **7**, 490–502.

2 A. Martens, P. Weis, M. C. Krummer, M. Kreuzer, A. Meierhöfer, S. C. Meier, J. Bohnenberger, H. Scherer, I. Riddlestone and I. Krossing, *Chem. Sci.*, 2018, **9**, 7058–7068.

3 J. Cosier and A. M. Glazer, *J. Appl. Crystallogr.*, 1986, **19**, 105–107.

4 Bruker, *SAINT, V8.40B*, Bruker AXS Inc., Madison, Wisconsin, USA.

5 L. Krause, R. Herbst-Irmer, G. M. Sheldrick and D. Stalke, *J. Appl. Crystallogr.*, 2015, **48**, 3–10.

6 G. M. Sheldrick, *Acta Crystallogr. C Struct. Chem.*, 2015, **71**, 3–8.

7 C. B. Hübschle, G. M. Sheldrick and B. Dittrich, *J. Appl. Crystallogr.*, 2011, **44**, 1281–1284.

8 D. Kratzert, J. J. Holstein and I. Krossing, *J. Appl. Crystallogr.*, 2015, **48**, 933–938.

9 O. V. Dolomanov, L. J. Bourhis, R. J. Gildea, J. A. K. Howard and H. Puschmann, *J. Appl. Crystallogr.*, 2009, **42**, 339–341.

10 C. R. Groom, I. J. Bruno, M. P. Lightfoot and S. C. Ward, *Acta Crystallogr. B Struct. Sci. Cryst. Eng. Mater.*, 2016, **72**, 171–179.

11 D. Kratzert, *FinalCif, V139*, 2025, https://dkratzert.de/finalcif.html.

12 P. R. Spackman, M. J. Turner, J. J. McKinnon, S. K. Wolff, D. J. Grimwood, D. Jayatilaka and M. A. Spackman, *J. Appl. Crystallogr.*, 2021, **54**, 1006–1011.

13 R. K. Harris, E. D. Becker, S. M. Cabral De Menezes, R. Goodfellow and P. Granger, *Magn. Reson. Chem.*, 2002, **40**, 489–505.
14 K. Herb, R. Tschaggelar, G. Denninger and G. Jeschke, *J. Magn. Reson.*, 2018, **289**, 100–106.
15 S. Stoll and A. Schweiger, *J. Magn. Reson.*, 2006, **178**, 42–55.
16 F. Neese, *WIREs Comput. Mol. Sci.*, 2022, **12**, e1606.
17 S. Grimme, A. Hansen, S. Ehlert and J.-M. Mewes, *J. Chem. Phys.*, 2021, **154**, 064103.
18 H. Kruse and S. Grimme, *J. Chem. Phys.*, 2012, **136**, 154101.
19 E. Caldeweyher, C. Bannwarth and S. Grimme, *J. Chem. Phys.*, 2017, **147**, 034112.
20 E. Caldeweyher, S. Ehlert, A. Hansen, H. Neugebauer, S. Spicher, C. Bannwarth and S. Grimme, *J. Chem. Phys.*, 2019, **150**, 154122.
21 W. Grimus, *arXiv*, 2011, preprint, DOI: 10.48550/ARXIV.1112.3748.
22 M. A. Tius, *Tetrahedron*, 1995, **51**, 6605–6634.
23 M. Sellin, C. Friedmann, M. Mayländer, S. Richert and I. Krossing, *Chem. Sci.*, 2022, **13**, 9147–9158.
24 K. M. Marczenko, H. P. A. Mercier, J. T. Goettel and G. J. Schrobilgen, *Angew. Chem. Int. Ed.*, 2025, **64**, e202425216.
25 Q. Wen and W. Jäger, *J. Phys. Chem. A*, 2007, **111**, 2093–2097.
26 S. Hirabayashi and K. M. T. Yamada, *Chem. Phys. Lett.*, 2006, **418**, 323–327.
